# Supplementary material for: Effects of paediatric schistosomiasis control programmes in sub-Saharan Africa: A systematic review
Source: PLoS One. 2024 May 2;19(5):e0301464. doi: 10.1371/journal.pone.0301464 (PMC11065241; doi:10.1371/journal.pone.0301464)
Supplement: S1 File — (PDF) [file pone.0301464.s003.pdf]

Database	Title	doi
Pubmed	**Association between water, sanitation, and hygiene access and the prevalence of soil-transmitted helminth and schistosome infections in Wolayita, Ethiopia**	10.1186/s13071-022-05465-7
	**School Water, Sanitation, and Hygiene, Soil-Transmitted Helminths, and Schistosomes: National Mapping in Ethiopia**	: 10.1371/journal.pntd.0004515
	**Does mass drug administration affect Schistosoma mansoni infection trends in West Dembia district, Northwest Ethiopia?**	10.3855/jidc.11727

**Urogenital schistosomiasis in three different water access in the Senegal river basin: prevalence and monitoring praziquantel efficacy and re-infection levels**	10.1186/s12879-022-07813-5
Efficacy of Praziquantel Treatment and Schistosoma Mansoni Infection among Primary School Children in Kemisse Town, Northeast Ethiopia	10.4314/ejhs.v32i3.20

**Reduction of urogenital schistosomiasis with an integrated control project in Sudan**	10.1371/journal.pntd.0003423
**Effectiveness of Mass Drug Administration on Neglected Tropical Diseases in Schoolchildren in Zanzibar, Tanzania**	

**Integrated community-based intervention for urinary schistosomiasis and soil-transmitted helminthiasis in children from Caxito, Angola**	10.1093/inthealth/ihz055
**Integrated Schistosomiasis and Soil-Transmitted Helminthiasis Control over Five Years on Kome Island, Tanzania**	10.3347/kjp.2015.53.5.535

**Impact of community-based integrated mass drug administration on schistosomiasis and soil-transmitted helminth prevalence in Togo**	10.1371/journal.pntd.0006551
**Schistosoma haematobium infection and morbidity before and after large-scale administration of praziquantel in Burkina Faso**	10.1086/520515
Prevalence and factors associated with persistent transmission of Schistosoma haematobium amongprimary school children after five rounds of mass drug administration using praziquantel: A cross sectional study in Mkuranga district, Tanzania	10.1177/00494755221103088

**Impact of a Novel, Low-Cost and Sustainable Health Education Program on the Knowledge, Attitudes, and Practices Related to Intestinal Schistosomiasis in School Children in a Hard-to-Reach District of Madagascar**	10.4269/ajtmh.21-0220
***Schistosoma mansoni* Mass Drug Administration Regimens and Their Effect on Morbidity among Schoolchildren over a 5-Year Period-Kenya, 2010-2015**	10.4269/ajtmh.18-0067

**Schistosomiasis in school-age children in Burkina Faso after a decade of preventive chemotherapy**	10.2471/BLT.15.161885
**Study and implementation of urogenital schistosomiasis elimination in Zanzibar (Unguja and Pemba islands) using an integrated multidisciplinary approach**	10.1186/1471-2458-12-930

**Urogenital schistosomiasis among pre-school and school aged children in four districts of north western Tanzania after 15 years of mass drug administration: Geographical prevalence, risk factors and performance of haematuria reagent strips**	10.1371/journal.pntd.0010834
**Evaluation of a urogenital schistosomiasis behavioural intervention among students from rural schools in Unguja and Pemba islands, Zanzibar**	10.1016/j.actatropica.2021.105960

**Pre- and post-intervention perceptions and water contact behaviour related to schistosomiasis in north-western Tanzania**	10.1016/j.actatropica.2012.09.017
**Impact of seven years of mass drug administration and recrudescence of Schistosoma haematobium infections after one year of treatment gap in Zanzibar: Repeated cross-sectional studies**	10.1371/journal.pntd.0009127

**Mass drug administration with praziquantel reduces the prevalence of Schistosoma mansoni and improves liver morbidity in untreated preschool children**	10.1093/trstmh/tru097
**Evaluation of Praziquantel Effectivenss After Decades of Prolonged Use in an Endemic Area in Egypt**	10.1007/s11686-020-00242-x
**Mass drug administration significantly reduces infection of Schistosoma mansoni and hookworm in school children in the national control program in Sierra Leone**	10.1186/1471-2334-12-16

**Evaluating the impact of biannual school-based and community-wide treatment on urogenital schistosomiasis in Niger**	10.1186/s13071-020-04411-9
**Benefits of annual chemotherapeutic control of schistosomiasis on the development of protective immunity**	10.1186/s12879-019-3811-z

**Effectiveness of Four Different Interventions Against Schistosoma haematobium in a Seasonal Transmission Setting of Côte d'Ivoire: A Cluster Randomized Trial**	10.1093/cid/ciab787
**Urogenital schistosomiasis in Nigeria post receipt of the largest single praziquantel donation in Africa**	10.1016/j.actatropica.2021.105916
**Effective control of Schistosoma haematobium infection in a Ghanaian community following installation of a water recreation area**	10.1371/journal.pntd.0001709

**A 5-Year intervention study on elimination of urogenital schistosomiasis in Zanzibar: Parasitological results of annual cross-sectional surveys**	10.1371/journal.pntd.0007268
**Impact of praziquantel mass drug administration campaign on prevalence and intensity of Schistosoma haemamtobium among school children in Bahi district, Tanzania**	10.4314/thrb.v16i1.1

**Prevalence of Schistosoma mansoni infection and the therapeutic efficacy of praziquantel among school children in Manna District, Jimma Zone, southwest Ethiopia**	10.1186/s13071-016-1833-6
**Evaluation of integrated interventions layered on mass drug administration for urogenital schistosomiasis elimination: a cluster-randomised trial**	10.1016/S2214-109X(19)30189-5

**Dynamics of Schistosoma haematobium egg output and associated infection parameters following treatment with praziquantel in school-aged children**	10.1186/1756-3305-5-298
**A 12-year follow-up of intestinal schistosomiasis in pre-school-aged children in Assoni Village, Eastern Senegal**	10.1186/s40249-021-00867-8

**Safety, efficacy and acceptability of praziquantel in the treatment of Schistosoma haematobium in pre-school children of Kwale County, Kenya**	10.1371/journal.pntd.0006852
Transmission Dynamics of Schistosoma haematobium among School-Aged Children: A Cohort Study on Prevalence, Reinfection and Incidence after Mass Drug Administration in the White Nile State of Sudan	10.3390/ijerph182111537

**Safety and efficacy of praziquantel syrup (Epiquantel®) against Schistosoma haematobium and Schistosoma mansoni in preschool-aged children in Niger**	10.1016/j.actatropica.2012.12.003
**Schistosoma haematobium infections among schoolchildren in central Sudan one year after treatment with praziquantel**	10.1186/1756-3305-5-108

Five-Year Impact of Different Multi-Year Mass Drug Administration Strategies on Childhood Schistosoma mansoni-Associated Morbidity: A Combined Analysis from the Schistosomiasis Consortium for Operational Research and Evaluation Cohort Studies in the Lake Victoria Regions of Kenya and Tanzania	10.4269/ajtmh.19-0273
Comparison of School-Based and Community-Wide Mass Drug Administration for Schistosomiasis Control in an Area of Western Kenya with High Initial Schistosoma mansoni Infection Prevalence: A Cluster Randomized Trial	10.4269/ajtmh.19-0626

**Population Pharmacokinetics and Pharmacodynamics of Praziquantel in Ugandan Children with Intestinal Schistosomiasis: Higher Dosages Are Required for Maximal Efficacy**	10.1128/mBio.00227-16
**Evaluation of morbidity in Schistosoma mansoni-positive primary and secondary school children after four years of mass drug administration of praziquantel in western Kenya**	10.1186/s40249-020-00690-7

	**Prevalence of urinary schistosomiasis in school-aged children in Langai, Plateau State: pre- and post-intervention**	
Ebsco Host	**Effect of an integrated intervention package of preventive chemotherapy, community-led total sanitation and health education on the prevalence of helminth and intestinal protozoa infections in Côte d'Ivoire**	10.1186/s13071-018-2642-x

**Cluster randomized trial comparing school-based mass drug administration schedules in areas of western Kenya with moderate initial prevalence of Schistosoma mansoni infections**	10.1371/journal.pntd.0006033
**Efficacy and safety of praziquantel in preschool-aged children in an area co-endemic for Schistosoma mansoni and S. haematobium**	10.1371/journal.pntd.0001917

**Ultrasonographic evaluation of urinary tract morbidity in school-aged and preschool-aged children infected with Schistosoma haematobium and its evolution after praziquantel treatment: A randomized controlled trial**	10.1371/journal.pntd.0005400

Comparison of the Impact of Different Mass Drug Administration Strategies on Infection with Schistosoma mansoni in Mwanza Region, Tanzania-A Cluster-Randomized Controlled Trial	10.4269/ajtmh.18-0671
**Impact of two rounds of praziquantel mass drug administration on Schistosoma mansoni infection prevalence and intensity: a comparison between community wide treatment and school based treatment in western Kenya**	10.1016/j.ijpara.2016.01.006

**Impact of Annual Praziquantel Treatment on Urogenital Schistosomiasis in a Seasonal Transmission Focus in Central Senegal**	10.1371/journal.pntd.0004557
**Urinary schistosomiasis in school children of a southern nigerian community 8 years after the provision of potable water**	10.4103/npmj.npmj_136_17

**Reinfection of urogenital schistosomiasis in pre-school children in a highly endemic district in Northern Zimbabwe: a 12 months compliance study**	10.1186/s40249-018-0483-7
**The influence of transmission season on parasitological cure rates and intensity of infection after praziquantel treatment of Schistosoma haematobium-infected schoolchildren in Mozambique**	10.1017/S0031182009006210

**Impact of iron supplementation on schistosomiasis control in Zambian school children in a highly endemic area**	10.4314/mmj.v21i1.10982
**Factors affecting infection or reinfection with Schistosoma haematobium in coastal Kenya: survival analysis during a nine-year, school-based treatment program**	
**Detection of duo-schistosome infection from filtered urine samples from school children in Zambia after MDA**	10.1371/journal.pone.0189400

**Impact of Single Dose Praziquantel Treatment on Schistosoma haematobium Infection among School Children in an Endemic Nigerian Community**	10.3347/kjp.2018.56.6.577
**Efficacy of praziquantel against Schistosoma mansoni with particular consideration for intensity of infection**	10.1046/j.1365-3156.2000.00646.x

Reinfection with Schistosoma haematobium and mansoni despite repeated praziquantel office treatment in Niger, Mali

Assessment of schistosomiasis prevalence among children 5 to 14 years old after several years of mass drug administration in the Senegal River basin

**Six rounds of annual praziquantel treatment during a national helminth control program significantly reduced schistosome infection and morbidity levels in a cohort of schoolchildren in Zimbabwe**	10.1371/journal.pntd.0008388
Dynamics of Egg Excretion of Schistosoma haematobium in a Longitudinal Cohort Under Treatment with Praziquantel over a Five-Year Period in Kalifabougou, Mali	10.3166/bspe-2018-0018

**Significantly reduced intensity of infection but persistent prevalence of schistosomiasis in a highly endemic region in Mali after repeated treatment**	10.1371/journal.pntd.0001774
**Two-year impact of single praziquantel treatment on infection in the national control programme on schistosomiasis in Burkina Faso**	10.2471/blt.07.048694

**Impact of community piped water coverage on re-infection with urogenital schistosomiasis in rural South Africa**	10.7554/eLife.54012
**Randomized comparison of low-dose versus standard-dose praziquantel therapy in treatment of urinary tract morbidity due to Schistosoma haema tobium infection**	10.4269/ajtmh.2002.66.725

**Efficacy of two praziquantel treatments among primary school children in an area of high Schistosoma mansoni endemicity, Nile Delta, Egypt**	10.1017/S003118201000154X
**Repeated doses of Praziquantel in Schistosomiasis Treatment (RePST) - single versus multiple praziquantel treatments in school-aged children in Côte d'Ivoire: a study protocol for an open-label, randomised controlled trial**	10.1186/s12879-018-3554-2

**Patterns of Schistosoma haematobium infection, impact of praziquantel treatment and re-infection after treatment in a cohort of schoolchildren from rural KwaZulu-Natal/South Africa**	10.1186/1471-2334-4-40
**Efficacy of praziquantel against urinary schistosomiasis and reinfection in Senegalese school children where there is a single well-defined transmission period**	10.1186/s13071-015-0980-5

**Parasitological impact of 2-year preventive chemotherapy on schistosomiasis and soil-transmitted helminthiasis in Uganda**	10.1186/1741-7015-5-27
**Effectiveness of school-based preventive chemotherapy strategies for sustaining the control of schistosomiasis in Côte d'Ivoire: Results of a 5-year cluster randomized trial**	10.1371/journal.pntd.0008845

**A randomised controlled clinical trial on the safety of co-administration of albendazole, ivermectin and praziquantel in infected schoolchildren in Uganda**	10.1016/j.trstmh.2010.11.012
**Morbidity due to Schistosoma mansoni: an epidemiological assessment of distended abdomen syndrome in Ugandan school children with observations before and 1-year after anthelminthic chemotherapy**	10.1016/j.trstmh.2005.12.013

**Effect of four rounds of annual school-wide mass praziquantel treatment for schistosoma mansoni control on schistosome-specific immune responses**	10.1111/pim.12530
**Praziquantel, mefloquine-praziquantel, and mefloquine-artesunate-praziquantel against Schistosoma haematobium: a randomized, exploratory, open-label trial**	10.1371/journal.pntd.0002975

**Controlling schistosomiasis: significant decrease of anaemia prevalence one year after a single dose of praziquantel in Nigerian schoolchildren**	10.1371/journal.pntd.0000241
**Regression of hepatosplenomegaly in Kenyan school-aged children after praziquantel treatment and three years of greatly reduced exposure to Schistosoma mansoni**	10.1016/j.trstmh.2004.06.009

**Prophylactic effect of artemether on human schistosomiasis mansoni among Egyptian children: A randomized controlled trial**	10.1016/j.actatropica.2016.02.015
**Uptake of mass drug administration programme for schistosomiasis control in Koome Islands, Central Uganda**	10.1371/journal.pone.0123673

**The impact of a school health programme on the prevalence and morbidity of urinary schistosomiasis in Mwera Division, Pangani District, Tanzania**	10.1016/s0035-9203(01)90333-5
**Drug efficacy of praziquantel and albendazole in school children in Mwea Division, Central Province, Kenya**	10.1016/j.actatropica.2007.04.017

**Control of Schistosoma mansoni by the soapberry Endod (Phytolacca dodecandra) in Wollo, northeastern Ethiopia: post-intervention prevalence**	10.4314/eamj.v79i4.8878
**Praziquantel treatment coverage among school age children against Schistosomiasis and associated factors in Ethiopia: a cross-sectional survey, 2019**	10.1186/s12879-020-05519-0

**Efficacy of single versus four repeated doses of praziquantel against Schistosoma mansoni infection in school-aged children from Côte d'Ivoire based on Kato-Katz and POC-CCA: An open-label, randomised controlled trial (RePST)**	[10.1371/journal.pntd.0008189]
**No apparent reduction in schistosome burden or genetic diversity following four years of school-based mass drug administration in mwea, central kenya, a heavy transmission area**	[10.1371/journal.pntd.0003221]

**Phenotypic and genotypic monitoring of Schistosoma mansoni in Tanzanian schoolchildren five years into a preventative chemotherapy national control programme**	10.1186/s13071-017-2533-6
**Defining Persistent Hotspots: Areas That Fail to Decrease Meaningfully in Prevalence after Multiple Years of Mass Drug Administration with Praziquantel for Control of Schistosomiasis**	10.4269/ajtmh.17-0368

**Effectiveness of a pre-treatment snack on the uptake of mass treatment for schistosomiasis in Uganda: a cluster randomized trial**	10.1371/journal.pmed.1001640
**Impact of water resource installations on the distribution of schistosomiasis and its intermediary hosts in Burkina Faso**	

**Schistosomiasis Sustained Control Program in Ethnic Groups Around Ninefescha (Eastern Senegal)**	10.4269/ajtmh.15-0125
**Effect of intensive treatment for schistosomiasis on immune responses to vaccines among rural Ugandan island adolescents: randomised controlled trial protocol A for the ' POP ulation differences in VACcine responses' (POPVAC) programme**	10.1136/bmjopen-2020-040426

**HEALTH EDUCATION AND THE CONTROL OF UROGENITAL SCHISTOSOMIASIS: ASSESSING THE IMPACT OF THE JUMA NA KICHOCHO COMIC-STRIP MEDICAL BOOKLET IN ZANZIBAR**	10.1017/S0021932016000122
**Schistosoma haematobium treatment in 1-5 year old children: safety and efficacy of the antihelminthic drug praziquantel**	10.1371/journal.pntd.0001143
**Impact of school health programme on urinary schistosomiasis control in schoolchildren in Kilosa, Tanzania**	10.4314/thrb.v7i3.14260

**Efficacy and safety of praziquantel in preschool-aged and school-aged children infected with Schistosoma mansoni: a randomised controlled, parallel-group, dose-ranging, phase 2 trial**	10.1016/S2214-109X(17)30187-0
**Control of urinary schistosomiasis on Zanzibar (Unguja Island): a pilot evaluation of the educational impact of the Juma na Kichocho health booklet within primary schools**	10.1590/s0074-02762006000900019

**Impact of health education programs on the control of urinary bilharziasis in Niger**	
**Schisto and Ladders version 2: a health educational board game to support compliance with school-based mass drug administration with praziquantel - a pilot study**	10.1093/inthealth/ihaa057
Pubmed	2018

2018

Ebscohost 04/03/2023

medline

medline

Methods

A population-based, cross-sectional census and parasitological mapping activity was conducted between 2018 and 2019. Individuals in the census were identified using either a registered study ID card or biometric fingerprint to enable linkage of their household WaSH data with baseline STH and schistosome prevalence for risk analysis.
In the 2013-2014 Ethiopian national mapping of infections with these parasites, school WASH was assessed alongside infection intensity in children, mostly between 10 and 15 years of age. Scores were constructed reflecting exposure to schistosomes arising from water collection for schools, from freshwater sources, and the adequacy of school sanitation and hygiene facilities. Kendall's τb was used to test the WASH scores against the school-level arithmetic mean intensity of infection with each

Data were extracted from routine laboratory logbooks a

The baseline prevalence of S. haematobium was determined in August 2020 in 777 children between 5 and 11 years old and a single dose of praziquantel (40 mg/kg) was administered to those positive. The efficacy of praziquantel and the re-infection rates were monitored 4 weeks and 7 months after treatment, respectively, in 226 children with a high intensity of infection at baseline.

A comparative cross-sectional study was conducted among 499 children of two preschool children. Stool specimens were collected and microscopically examined using Kato-Katz (41.7 gram) methods. Positive children were treated with a single oral dose of praziquantel at 40 mg/kg body weight. Egg reduction and cure rates were assessed 4 weeks post-treatment to evaluate the therapeutic efficacy of praziquantel against S. mansoni infection.

The total population of the project sites was 482,902, and the major target group for intervention among them was 78,615 primary school students. For the cross-sectional study of the prevalence, urine and stool specimens were examined using the urine sedimentation method and the Kato cellophane thick smear method, respectively. To assess the impacts of health education for students and a drinking water supply facility at Al Hidaib village, questionnaire survey was done.

Soil-transmitted helminths and Schistosoma haematobium affect more than 3 billion people globally and mainly occur in sub-Saharan Africa. The present study assessed the overall infection status of a 1716-student cohort of school-children in Zanzibar and applied mass drug administration (MDA) to the cohort from 2007 to 2009. Schools in Pemba, Zanzibar, had a much higher prevalence of soil-transmitted helminth infections than those in Unguja, and the Chaani, Ghana, and Machui schools of Unguja exhibited high S. haematobium infection rates. The MDA program only partially controlled parasite infections, owing to high rates of re-infection.

We examined children (aged 2-15 y) from one hamlet, who provided urine and faeces samples at baseline (n=197), 1 mo (n=102) and 6 mo (n=92); 67 completed the protocol.

With the support of Good Neighbors International in collaboration with National Institute of Medical Research, Mwanza, Tanzania, integrated control applying mass drug administration (MDA), health education using PHAST, and improved safe water supply has been implemented on Kome Island over 5 years for controlling schistosomiasis and soil-transmitted helminths (STHs). Baseline surveys for schistosomiasis and STHs was conducted before implementation of any integrated control strategies, followed by 4 cross-sectional follow-up surveys on randomly selected samples of schoolchildren and adults in 10 primary schools and 8 villages, respectively, on Kome islands. Those follow-up surveys were conducted for impact evaluation after introduction of control strategies interventions in the study area.

In every sub-district in the country outside the capital, the same schools were visited as at baseline and a sample of fifteen children age 6 to 9 years old was drawn. Each child submitted urine and a stool sample. Urine samples were tested by dipstick for the presence of blood as a proxy measure of Schistosoma haematobium infection. Stool samples were analyzed by the Kato-Katz method for STH and Schistosoma mansoni. At baseline, 17,100 children were enrolled at 1,129 schools in 562 sub-districts; in 2015, 16,890 children were enrolled at the same schools.

We describe a longitudinal epidemiological study that evaluates the relationship between S. haematobium infection and associated morbidity in children before and after the large-scale administration of praziquantel for schistosomiasis and albendazole for soil-transmitted helminths.

Despite a human schistosomiasis control programme through praziquantel mass drug administration (MDA) between 2011 and 2015,there was still persistent transmission among primary schoolchildren (PSC) in Mkuranga district, Tanzania. Our cross-sectional study was conducted among 396 PSC who provided urine for diagnosis of Schistosoma haematobium infection. Observations were conducted to determine PSC water contact activities.

Schistosomiasis control requires multisectoral approaches including praziquantel treatment, access to safe water, sanitation and hygiene, and health education. Community input can help ensure health education programs are culturally appropriate to effectively direct protective behavior change. This study reports on the three-stage development of an education program for Malagasy children, with an impact evaluation on their knowledge, attitudes, and practices (KAP) related to intestinal schistosomiasis. A cross-sectional study took place in 2017 with follow-up in 2018 in the hard-to-reach Marolambo district, Madagascar. A novel schistosomiasis education program (SEP) was designed in collaboration with researchers, stakeholders, and local community and included cartoon books, games, songs, puzzles, and blackboard lessons, costing $10 USD per school. KAP questionnaires were completed by 286 children pre-SEP and 273 children post-SEP in 2017, and by 385 and 337 children pre-SEP and post-SEP, respectively, in 2018.

Schistosomiasis control programs are designed to reduce morbidity by providing mass drug administration (MDA) of praziquantel to at-risk populations. We compared morbidity markers between two cohorts of Kenyan schoolchildren that initially had high prevalence of Schistosoma mansoni infections. One cohort (N = 416 at year 1) received four rounds of annual MDA in a community-wide treatment (CWT) strategy. The other cohort (N = 386 at year 1) received school-based treatment (SBT) every other year over the 4-year period. We measured infection with S. mansoni and soil-transmitted helminths (STH) as well as subtle morbidity markers at year 1, year 3, and year 5 and compared cohorts with mixed models after controlling for age and gender.

In 2013, in a national assessment based on 22 sentinel sites, 3514 school children aged 7-11 years were checked for Schistosoma haematobium and Schistosoma mansoni infection by the examination of urine and stool samples, respectively. We analysed the observed prevalence and intensity of infections and compared these with the relevant results of earlier surveys in Burkina Faso.

In this 5-year research study, on both Unguja and Pemba islands, urogenital schistosomiasis will be assessed in 45 communities with urine filtration and reagent strips in 4,500 schoolchildren aged 9-12 years annually, and in 4,500 first-year schoolchildren and 2,250 adults in years 1 and 5. Additionally, from first-year schoolchildren, a finger-prick blood sample will be collected and examined for Schistosoma haematobium infection biomarkers. Changes in prevalence and infection intensity will be assessed annually. Among the 45 communities, 15 were randomized for biannual snail control with niclosamide, in concordance with preventive chemotherapy campaigns.

A analytical cross-sectional study was conducted between October and November, 2019 among pre-school (3-5years old) and school aged children (6-17 years old) living in four (4) districts with low (<10%) and moderate (10%-<50%) endemicity for schistosomiasis as per WHO classification at the start of the national control programme in 2005/06, with mean prevalence of 20.7%. A total of 20,389 children from 88 randomly selected primary schools participated in the study. A questionnaire was used to record demographic information. A single urine sample was obtained from each participant and visually examined for macrohaematuria, tested with a dipstick for micro-haematuria, to determine blood in urine; a marker of schistosome related morbidity and a proxy of infection. Infection intensity was determined by parasitological examination of the urine sample for S. haematobium eggs

Urogenital schistosomiasis is a common experience among children in Zanzibar. There is a paucity of behavioural science-based, health education and behaviour change (HEBC) interventions for school-aged children, those at greatest risk for urogenital schistosomiasis. We assessed the influence of a HEBC intervention, guided by the Health Belief model, among rural schoolchildren on Pemba and Unguja islands in Zanzibar, Tanzania. From 2012 to 2016, a cluster-randomized trial to assess three different interventions against urogenital schistosomiasis was conducted in 90 schools and shehias across Zanzibar. The HEBC intervention was implemented in 15 schools per island. In 2017, at the trial conclusion, we administered written questionnaires to schoolchildren from 4 HEBC intervention schools and 4 not HEBC exposed schools on each island, respectively. Responses were compared between students that were exposed or not exposed to the HEBC intervention using a Fisher's exact test.

A study on schistosomiasis-related perceptions and water contact behaviour was undertaken in one community population of Hamuyebe village in Ukerewe district, north-western Tanzania, where intestinal schistosomiasis is endemic before and 2 years after implementation of a participatory hygiene and sanitation transformation (PHAST) intervention. Data were obtained from baseline and post-intervention knowledge, attitudes and practices (KAP) questionnaire surveys conducted between 2008 and 2010 among 157 individuals aged 15 years and above. The surveys were further complemented by structured observations of human-water contact activities. We found significant increases in respondents' knowledge of the cause, transmission, symptoms and health consequences of schistosomiasis after the intervention.

Repeated cross-sectional surveys were conducted from 2011/12 till 2020 in 90 communities and 90 schools in Zanzibar. Annually, around 4,500 adults and up to 20,000 schoolchildren were surveyed. The S. haematobium prevalence was detected by urine filtration and reagent strips. In 2020, risk factors for infection were investigated using generalized estimated equation models.

In April 2008, parasitological (using the Kato-Katz method) and morbidity (determined by portal vein score) data were collected from 263 schoolchildren aged 6 and 7 years. The children had never received praziquantel. In March 2010, following two annual rounds of mass drug administration, 207 children aged 8 and 9 years old were examined to determine the effect of treatment. In addition, 158 untreated 6-year-olds were assessed to compare with the untreated children from 2008.

A total of 342 children aged 5-15 years living in Kafr-El-Sheikh were screened for S. mansoni infection. Stool samples were examined microscopically using Kato-Katz (KK) technique. Among the screened children, 106 children had S. mansoni ova in stool, 100 of them received the first dose of PZQ (40 mg/kg). Four weeks later, 96 of 100 children received the second dose of PZQ. Stool samples, collected 4 weeks after each dose of PZQ, were examined using KK. The effectiveness of PZQ was assessed based on ERR and CR.

Fifteen sentinel schools from six highly endemic districts (according to data from national and pre-MDA surveys) with Schistosoma mansoni affecting over 50% of the population, and moderate to high prevalence of hookworms (> 20%). Approximately 30 children aged 9-14 years were selected from each school and stool samples (one per student) were examined by the Kato-Katz method.

This was a cluster-randomised trial investigating six possible combinations of annual or biannual community-wide treatment (CWT), school-based treatment (SBT), and holidays from mass treatment over four years. The most intense arm involved two years of annual CWT followed by 2 years of biannual CWT, while the least intensive arm involved one year of annual SBT followed by a year without treatment and two more years of annual SBT. The primary outcome of interest was prevalence and intensity of Schistosoma haematobium among 100 children aged 9-12 years sampled each year. In addition, 100 children aged 5-8 years in their first year of school and 50 adults (aged 20-55 years) were tested in the first and final fifth year of the study.

Urine specimens from 212 school children (7-13 years) were collected and examined to determine prevalence, intensity and reinfection of S.haematobium at baseline, 6 weeks and 2 years following annual rounds of praziquantel treatment. Blood samples from the participants were assayed for total and S. haematobium (Sh13)-specific antibodies before and 2 years after annual rounds of treatment.

Sixty-four localities with a S. haematobium prevalence in school children aged 13-14 years above 4% were randomly assigned to 1 of 4 intervention arms over a 3-year period: (1) the current standard strategy consisting of annual MDA before peak of transmission, (2) annual MDA after peak of transmission, (3) biannual MDA, and (4) standard MDA combined with snail control. The primary outcome was prevalence and intensity of S. haematobium infection in children aged 9-12 years 1 year after the final intervention, using urine filtration performed by experienced microscopists.

Schistosomiasis control efforts in Nigeria received a boost in 2016 when Merck Group made the largest single donation of praziquantel to an African country. We examined urine samples from 2,023 school age children from 15 locations in 10 states and an Internally Displaced Person's (IDP) camp in Nigeria. We recorded an overall Schistosoma haematobium prevalence of 10.4% in the 10 states that ranged between 6 - 37%, while prevalence in the IDP camp was 2.9%. The highest infection prevalence (37%) recorded was from the population in Wasai Dam area in Minjibir (Kano State), while five locations had no positive urine samples.

We designed a water recreation area (WRA) to prevent transmission to school-aged children. The WRA features a concrete pool supplied by a borehole well and a gravity-driven rainwater collection system; it is 30 m(2) and is split into shallow and deep sections to accommodate a variety of age groups. The WRA opened in 2009 and children were encouraged to use it for recreation as opposed to the local river. We screened children annually for S. haematobium eggs in their urine in 2008, 2009, and 2010 and established differences in infection rates before (2008-09) and after (2009-10) installation of the WRA. After each annual screening, children were treated with praziquantel and rescreened to confirm parasite clearance.

A repeated cross-sectional cluster-randomized trial was implemented from 2011/12 till 2017. On each island, 45 shehias were randomly assigned to receive one of three interventions: biannual mass drug administration (MDA) with praziquantel alone, or in combination with snail control or behavior change measures. In cross-sectional surveys, a single urine sample was collected from ~9,000 students aged 9- to 12-years and from ~4,500 adults aged 20- to 55-years annually, and from ~9,000 1st year students at baseline and the final survey. Each sample was examined for S. haematobium eggs by a single urine filtration. Prevalence and infection intensity were determined. Odds of infection were compared between the intervention arms.

The objectives of this study were to assess the impact of the two rounds of MDA on prevalence and intensity of Schistosoma haemamtobium and the impact of MDA campaigns on knowledge of urinary schistosomiasis, safe water use and contact with potentially unsafe water bodies. A quantitative cross-sectional study was carried out among schoolchildren in March and April, 2013. A structured questionnaire was used to collect information on MDA uptake, knowledge of schistosomiasis, sources of water for domestic and other uses. Urine samples were collected from each pupil to examine prevalence and intensity of S. haematobium. Transmission of schistosomiasis was assessed by sampling Bulinus spp snails for cercarial shedding.

A cross-sectional study was conducted among the school children aged between 6 and 18 years in three primary schools in Manna district from March to April 2014. For diagnosis of S. mansoni, a single stool sample was obtained from each child and processed using single Kato Katz and examined under light microscopy. A questionnaire was used to collect demographic information of the school children participated in the study. School children excreting eggs of S. mansoni were administered with 40 mg/kg of PZQ and re-examined after three weeks post-treatment. The therapeutic efficacy of PZQ against S. mansoni was evaluated by means of cure rate and egg reduction rate.

In a 5-year repeated cross-sectional cluster-randomised trial, 90 shehias (small administrative regions; clusters) in Zanzibar eligible owing to available natural open freshwater bodies and public primary schools were randomly allocated (ratio 1:1:1) to receive one of three interventions: biannual MDA with praziquantel alone (arm 1) or in combination with snail control (arm 2), or behaviour change activities (arm 3). Neither participants nor field or laboratory personnel were blinded to the intervention arms. From 2012 to 2017, annually, a single urine sample was collected from approximately 100 children aged 9-12 years in the main public primary school of each shehia. The primary outcome was S haematobium infection prevalence and intensity in 9-12-year-old children after 5 years of follow-up. This study is completed and was registered with the ISRCTN, number 48837681.

Ninety school-aged children from south Côte d'Ivoire with a parasitologically confirmed S. haematobium infection were treated with a single oral dose of praziquantel (40 mg/kg) and followed up for 62 days post-treatment. Urine samples were collected on 23 schooldays during this period and were subjected to visual examination (macrohaematuria), urine filtration and microscopy (S. haematobium eggs) and reagent strip testing (microhaematuria, proteinuria and leukocyturia).

From 2008 to 2020, we (i) monitored the prevalence of S. mansoni in PSAC in Assoni using double-stool smear preparation, (ii) treated the infected PSAC with a standard dose of praziquantel 40 mg/kg, (iii) ran educational campaigns each year in the village, and (iv) built latrines to improve sanitation and reduce schistosomiasis transmission. Linear regression was used to examine the trend in the annual schistosomiasis prevalence and a two-sided of Chi-squared test was used to compare prevalence between the different age groups of PSAC.

400 PSC were enrolled, from 10 randomly selected ECDE Centers in Kwale County, Kenya where children were treated with crushed PZQ tablets mixed with orange juice, at a single dose of 40 mg/kg. Adverse events were assessed 24 hours post-treatment through questionnaires administered to the parents or guardians. Acceptability was determined by observing if the child spat and/ or vomited all or part of the PZQ dose immediately after treatment. Efficacy was assessed by examining urine samples for Schistosoma haematobium eggs in the 5 weeks post-treatment follow-up. Children testing negative for S. haematobium during the follow-up were considered cured. Egg reduction rate (ERR) was calculated as the decrement in the infection intensity (group's geometric mean egg counts per 10 ml of urine) following treatment expressed as a proportion of the pre-treatment infection intensity.

The reinfection rate of schistosomiasis after mass drug administration (MDA) has not been documented in Sudan. We aimed to explore the transmission dynamics of urogenital schistosomiasis after MDA, targeting school-aged children in the White Nile State of Sudan, assessing the prevalence, reinfection rate, and incidence. A single dose of praziquantel (40 mg/kg) was administered to 1951 students in five primary schools from January to February 2018 immediately after a baseline survey, and follow-up surveys were performed at 2 weeks and 6 months after treatment. We examined Schistosoma haematobium eggs by centrifugation methods.

e assessed the safety and efficacy of praziquantel syrup (Epiquantel®) in preschool-aged children in three villages of Niger. Children aged ≤72 months provided multiple urine and stool samples that were microscopically examined using standard protocols. Schistosoma-positive children were treated with praziquantel syrup at a dose of 40 mg/kg after a meal of millet porridge. Children remained under medical supervision for 4h and adverse events were recorded. Additionally, a questionnaire was administrated to the mothers/guardians 24h post-treatment for further probing of adverse events. Treatment efficacy was evaluated 3 and 6 weeks post-treatment using multiple stool and urine samples. A third of the 243 treated children reported adverse events within 4h, whilst a further 6.2% reported adverse events upon probing 24h post-treatment.

A longitudinal study was conducted to evaluate the impact of PZQ for the treatment of Schistosoma haematobium infection among schoolchildren at Al Salamania, Central Sudan. Parasitological examinations for S. haematobium were performed in a cohort of schoolchildren (6-15 years of age) before and 1 year after treatment with a single dose of PZQ 40 mg/kg.

The WHO recommends mass treatment with praziquantel as the primary approach for Schistosoma mansoni-related morbidity control in endemic populations. The Schistosomiasis Consortium for Operational Research and Evaluation implemented multi-country, cluster-randomized trials to compare effectiveness of community-wide and school-based treatment (SBT) regimens on prevalence and intensity of schistosomiasis. To assess the impact of two different treatment schedules on S. mansoni-associated morbidity in children, cohort studies were nested within the randomized trials conducted in villages in Kenya and Tanzania having baseline prevalence ≥ 25%. Children aged 7-8 years were enrolled at baseline and followed to ages 11-12 years. Infection intensity and odds of infection were reduced both in villages receiving four years of annual community-wide treatment (CWT) and those who received biennial SBT over 4 years.

We conducted a cluster randomized trial comparing the target population and timing of mass drug administration (MDA) with praziquantel for control of schistosomiasis in villages in western Kenya with high initial prevalence (> 25%) according to a harmonized protocol developed by the Schistosomiasis Consortium for Operational Research and Evaluation. A total of 150 villages were randomized into six treatment arms (25 villages per arm), were assessed at baseline, and received two or four rounds of MDA using community-wide (CWT) or school-based (SBT) treatment over 4 years. In the fifth year, a final evaluation was conducted. The primary outcomes were prevalence and intensity of Schistosoma mansoni infections in children aged 9-12 years, each year their village received MDA.

e conducted the first PZQ pharmacokinetic (PK) and pharmacodynamic (PD) study in young children comparing dosing. Sixty Ugandan children aged 3 to 8 years old with egg patent Schistosoma mansoni received PZQ at either 40 mg/kg or 60 mg/kg. PK parameters of PZQ racemate and enantiomers (R and S) were quantified. PD outcomes were assessed by standard fecal egg counts and novel schistosome-specific serum (circulating anodic antigen [CAA]) and urine (circulating cathodic antigen [CCA]) antigen assays. Population PK and PD analyses were performed to estimate drug exposure in individual children, and the relationship between drug exposure and parasitological cure was estimated using logistic regression. Monte Carlo simulations were performed to identify better, future dosing regimens. There was marked PK variability between children, but the area under the concentration-time curve (AUC) of PZQ was strongly predictive of the parasitological cure rate (CR). Although no child achieved antigenic cure, which is suggestive of an important residual adult worm burden, higher AUC was associated with greater CAA antigenic decline at 24 days. To optimize the performance of PZQ, analysis of our simulations suggest that higher doses (>60 mg/kg) are needed, particularly in smaller children.

Between 2012 and 2016 all students in two primary and three secondary schools within three kilometers of Lake Victoria in western Kenya received annual mass praziquantel administration. To evaluate potential changes in morbidity we measured height, weight, mid-upper arm circumference, hemoglobin levels, abdominal ultrasound, and quality of life in children in these schools. This study compared two cross-sectional samples of Schistosoma mansoni egg-positive children: one at baseline and one at year five, 1 year after the fourth annual MDA. Data were analyzed for all ages (6-18 years old) and stratified by primary (6-12 years old) and secondary (12-18 years old) school groups.

Two hundred and eighteen children (218) subjects who were selected by multi-staged sampling methods were administered semi-structured questionnaires and their urine samples were assayed for S. haematobium ova using the sedimentation method before and after drug treatment with Praziquantel tablets

A cross-sectional survey was carried out in nine communities of south-central Côte d'Ivoire to assess people's infection with helminths and intestinal protozoa and KAPB. Subsequently, interventions were targeted to five communities, while the remaining communities served as control. The intervention encouraged latrine construction and an evaluation was done 6-7 months later to determine open defecation status of the respective communities. Anthelminthic treatment was provided to all community members. A follow-up cross-sectional survey was conducted approximately one year later, using the same procedures.

We performed a cluster randomized trial (ISRCTN 14849830) of 3 different MDA frequencies over a 5 year period in 75 villages with moderate (10%-24%) initial prevalence of S. mansoni in school children in western Kenya. Praziquantel was distributed by school teachers to students either annually, the first 2 years, or every other year over a 4 year period. Prevalence and intensity of infection were measured by stool examination in 9-12 year old students using the Kato-Katz method at baseline, each treatment year, and for the final evaluation at year 5. S. mansoni prevalence and intensity were also measured in first year students at baseline and year 5.

We assessed the efficacy and safety of crushed praziquantel tablets among preschool-aged children (<6 years) in the Azaguié district, south Côte d'Ivoire, where Schistosoma mansoni and S. haematobium coexist. Using a cross-sectional design, children provided two stool and two urine samples before and 3 weeks after treatment. Crushed praziquantel tablets, mixed with water, were administered at a dose of 40 mg/kg. Adverse events were assessed and graded 4 and 24 hours posttreatment by interviewing mothers/guardians.

Our study was embedded in a randomized, placebo-controlled, single-blind trial in Côte d'Ivoire, which evaluated the efficacy and safety of three doses (20, 40 and 60 mg/kg) of praziquantel in school-aged (SAC) and preschool-aged (PSAC) children infected with S. haematobium. Enrolled children were invited to participate in an ultrasound examination prior and six months after treatment. At these time points 3 urine samples were collected for parasitological and clinical examinations.

Annual school-based mass drug administration with praziquantel has been widely implemented to control schistosomiasis, but other treatment strategies could have a different impact. The aim of this study was to investigate the impact of six different treatment strategies on Schistosoma mansoni infection in a cluster-randomized controlled trial in schoolchildren, in a high transmission area of the Mwanza Region, Tanzania. A total of 150 villages were randomized into six arms with 25 villages in each arm. In each village, approximately 100 schoolchildren aged 9-12 years were randomly selected each year and investigated for S. mansoni prevalence and intensity based on three consecutive stool samples using the duplicate Kato-Katz technique. Four years of community-wide treatment (CWT) was the most intensive treatment strategy, whereas 2 years of school-based treatment (SBT) combined with 2 years without treatment (holiday) was the least intensive treatment.

This study compared the effectiveness of the community-wide treatment and school-based treatment approaches in the control of Schistosoma mansoni infections in villages with ⩾25% prevalence in western Kenya. Stool samples from first year students, 9-12year olds and adults (20-55years) were analyzed by the Kato-Katz technique for S. mansoni eggs. After two rounds of treatment, S. mansoni prevalence and intensity levels significantly declined in both treatment approaches.

The aim of this study was to determine the effect of annual treatment over 3 years on the seasonal transmission dynamics of S. haematobium in 9 villages in the Niakhar district. Adults and children aged between 5 and 60 years were surveyed from 2011 to 2014. Urine samples were collected door-to-door and examined for S. haematobium eggs at baseline in June 2011, and all participants were treated in August 2011 with PZQ (40 mg/kg). After this initial examination, evaluations were conducted at 3 successive time points from September 2011 to March 2014, to measure the efficacy of the annual treatments and the rates of reinfection. Each year, during the transmission period, from July to November-December, malacological surveys were also carried out in the fresh water bodies of each village to evaluate the infestation of the snail intermediate hosts.

A cross-sectional survey was carried out among school and children aged 5-14 years in Adim community in Cross River State using the polyamide millipore filter technique and ova detection and count compared with the situation that obtained 8 years earlier before the provision of potable water. The prevalence and intensity of haematuria and proteinuria by reagent strips were also compared between the two eras.

The study was conducted from February 2016-February 2017 in Madziwa area, Shamva district. Following community mobilisation, mothers brought their children aged 5 years and below for recruitment at baseline and also urine sample collection at baseline, 3, 6, 9 and 12 months follow up surveys. At each time point, urine was tested for urogenital schistosomiasis by urine filtration and children found positive received treatment. Schistosoma haematobium prevalence, reinfections as well as children participation, and urine sample submission at each visit were assessed at each time point for one year.

To examine this hypothesis, a study was carried out in southern Mozambique. Following demonstration of seasonal transmission, PZQ was administered separately to two cohorts of S. haematobium-infected schoolchildren in (1) the high and (2) the low transmission seasons and followed up after two months when levels of infection and intensities were measured. The prevalence of infection decreased from 54.2% and 51.7% in cohorts 1 and 2 to 30.3% and 1.8%, respectively. The geometric mean intensity of infection decreased from 23.3 eggs/10 ml of urine at baseline to 15.6 eggs/10 ml of urine in cohort 1 (treated during high transmission season), and from 23.5 eggs/10 ml urine to 7.3 eggs/10 ml of urine in cohort 2 (treated during low transmission season).

Pupils in the intervention group received once weekly dose of ferrous sulphate at 200 mg while those in the control received once weekly vitamin C at 100 mg for up to 9 months. Both study groups received a single dose of praziquantel at baseline.

Urinary schistosomiasis remains a significant burden for Africa and the Middle East. Success of regional control strategies will depend, in part, on what influence local environmental and behavioral factors have on individual risk for primary infection and/or reinfection. Based on experience in a multi-year (1984-1992), school-based Schistosoma haematobium control program in Coast Province, Kenya, we examined risk for infection outcomes as a function of age, sex, pretreatment morbidity, treatment regimen, water contact, and residence location, with the use of life tables and Cox proportional-hazards analysis.

For control strategies based on targeted mass drug administration (MDA) to succeed it is essential to have a simple and sensitive test for monitoring the success of these interventions. Current available diagnostic tests, such as egg detection in stool by Kato-Katz (KK) for S. mansoni and detection of eggs or blood (hematuria) in urine for S. haematobium have reduced sensitivity in low intensity settings. The objective of the study was to evaluate active single or duo schistosome infections in school children following MDA using molecular diagnostics (PCR) on filtered urine samples and comparing that against traditional diagnostic tests.

Schistosomiasis is prevalent in Nigeria, and the foremost pathogen is Schistosoma haematobium, which affects about 29 million people. Single dose of the drug praziquantel is often recommended for treatment but the efficacy has not been documented in certain regions. Therefore, this study was designed to assess the impact of single dose praziquantel treatment on S. haematobium infection among school children in an endemic community of South-Western Nigeria. Urine samples were collected from 434 school children and 10 ml was filtered through Nucleopore filter paper before examination for egg outputs by microscopy. The prevalence was 24.9% at pre-treatment.

Chemotherapy with praziquantel is the cornerstone of schistosomiasis control. In view of recent concern about tolerance or resistance to praziquantel, monitoring its efficacy in different epidemiological settings is required. We report a study among 253 schoolchildren in an area highly endemic for Schistosoma mansoni in western Côte d'Ivoire. After examining four consecutive stool specimens from each child, the first praziquantel treatment at 60 mg/kg divided into two doses was administered. Four weeks later, stool specimens were again screened over 4 consecutive days and revealed a cure rate of 71.6% and an egg reduction rate of 79.9%.

The dynamics of reinfection by Schistosoma haematobium and Schistosoma mansoni after repeated treatment with praziquantel (40 mg/kg body weight, single dose) was studied in a cohort of schoolchildren living in an endemic area. A total of 214 urine and 220 stool samples were collected and examined at three different times, i.e., February 1989, July 1989 and February 1990. Mass chemotherapy was administered at the beginning of study (February 89). Treatment was repeated in children with positive tests at each subsequent sampling. Prevalence rates were 55.1 p. 100, 3.7 p. 100, and 35.0 p. 100 for Schistosoma haematobium and 62.7 p. 100, 46.3 p. 100 and 73.1 p. 100 for Schistosoma mansoni in February 1989, July 1989 and February 1990 respectively ($p < 0.001$). From July 1989 to February 1990, reinfection was observed in 84.5 p. 100 of children by Schistosoma haematobium versus 57.8 p. 100 by Schistosoma mansoni.

ntroduction: The advent of Diama and Manantali dams in the eighties has altered the schistosomiasis profile in the Senegal River Basin, with the appearance of an intestinal form in the Delta and a high prevalence of the urinary form in all ecological areas of the basin. Methods: The present study was mainly designed to re-evaluate the prevalence of schistosomiasis after many years of mass drug administration with praziquantel 600 mg allowing analysis of the pertinence of World Health Organisation guidelines in terms of dosing frequency, particularly in the Senegal River Basin. Stools and urine from 1,215 public school children from 24 villagesidentified in three ecological areas of the Senegal River Basin (Delta, valley, upper basin), were examined.

A school-based longitudinal study was carried out in 35 sentinel sites across Zimbabwe from September 2012 to November 2017. The sentinel sites were selected following a countrywide survey conducted in 280 primary schools. Schistosoma haematobium was diagnosed using the urine filtration technique. Schistosoma mansoni was diagnosed using both the Kato-Katz and formol-ether concentration techniques. S. haematobium morbidity was determined through detection of macro and microhaematuria. A cohort of children aged 6-15 years old was surveyed annually before MDA and 6 weeks post treatment. Maximum treatment coverage reached 90% over the 6 rounds of MDA.

This study aim was to evaluate the dynamics of Schistosoma haematobium eggs excretion after the scaling up of "Mass Drug Administration" (MDA) with praziquantel (PZQ) from 2011 to 2016 in a cohort of volunteers living in the village of Kalifabougou, Mali. We conducted a cross-sectional study on 676 volunteers in May 2011 niched in cohort study from 696 volunteers aged three months to 25 years. The eggs of Schistosoma haematobium (Sh) were tested by urine filtration technique, Soil-transmitted helminth and Schistosoma mansoni by the Kato-Katz technique. Maximal MDA/ PZQ population coverage was 83% in 2015 and no MDA/PZQ n 2014. A total of 676 volunteers was included in this prospective cohort.

The survey was conducted in six sentinel schools in three highly-endemic districts, and 640 school children aged 7-14 years were examined. Infections with Schistosoma haematobium and S. mansoni were diagnosed with the urine filtration and the Kato-Katz method respectively. Overall prevalence of S. haematobium infection was 61.7%, a significant reduction of 30% from the baseline in 2004 ($p<0.01$), while overall prevalence of S. mansoni infection was 12.7% which was not significantly different from the baseline. Overall mean intensity of S. haematobium and S. mansoni infection was 180.4 eggs/10 ml of urine and 88.2 epg in 2004 respectively.

A cohort of 1727 schoolchildren (6-14 years old) was monitored at yearly intervals through a longitudinal survey. Additional groups of schoolchildren were monitored in cross-sectional surveys. Parasitological examinations for Schistosoma haematobium and Schistosoma mansoni were performed, and prevalence and intensity of infection before and after treatment were analysed.

Previously, we demonstrated that coverage of piped water in the seven years preceding a parasitological survey was strongly predictive of Schistosomiasis haematobium infection in a nested cohort of 1976 primary school children (Tanser, 2018). Here, we report on the prospective follow up of infected members of this nested cohort (N = 333) for two successive rounds following treatment.

At present, anthelmintic therapy with praziquantel at a dose of 40 mg/kg of body weight is the recommended treatment for control of urinary tract morbidity caused by Schistosoma haematobium. Although this standard regimen is effective, drug cost may represent a significant barrier to implementation of large-scale schistosomiasis control programs in developing areas. Previous comparison trials have established that low-dose (20-30 mg/kg) praziquantel regimens can effectively suppress the intensity of S. haematobium infection in endemic settings. However, the efficacy of these low-dose regimens in controlling infection-related morbidity has not been determined in a randomized field trial.

Praziquantel is the cornerstone of schistosomiasis control. A number of reports from endemic areas suggest that resistance or tolerance to praziquantel might exist in Schistosoma mansoni. Several explanations were postulated. The present work was designed to test the hypothesis that a low praziquantel (pzq) cure rate in Egypt is due to survival and maturation of immature stages that escaped pzq, which is effective against mature S. mansoni worms only. The study sample included 1351 children attending El Rouse primary school located in El Rouse village, Nile Delta, Egypt. All children received 2 pzq doses (40 mg/kg) 4 weeks apart.

An open-label, randomised controlled trial will be conducted in school-aged children (5 to 18 years) from the region of Taabo, Côte d'Ivoire, an area endemic for S. mansoni. This 8-week trial includes four two-weekly standard doses of PZQ in the "intense treatment" intervention group and one standard dose of PZQ in the "standard treatment" control group. The efficacy of PZQ will be evaluated in stool samples using the KK technique and real-time PCR as well as in urine using the point-of-care circulating cathodic antigen test and the up-converting phosphor, lateral flow, circulating anodic antigen assay. The primary outcome of the study will be the difference in CR of intense versus standard treatment with PZQ on individuals with a confirmed S. mansoni infection measured by KK. Secondary outcomes include the difference in CR and intensity reduction rate between the intense and standard treatment groups as measured by the other diagnostic tests, as well as the accuracy of the different diagnostic tests, and the safety of PZQ.

Primary schoolchildren from Maputaland in northern KwaZulu-Natal were examined for Schistosoma haematobium infection, treated with praziquantel and re-examined four times over one year after treatment in order to assess the impact of treatment and patterns of infection and re-infection.

The current study was carried out in a cohort of 329 children aged five to 15 years enrolled from six villages in Niakhar to determine the efficacy of one dose of PZQ, as well as reinfection. Parasitological screening was performed in June 2011 to determine the baseline prevalence of S. haematobium, and then a single dose of PZQ was administered to all selected subjects in the transmission season in August 2011. The efficacy of PZQ treatment and reinfection were monitored respectively five weeks after in September 2011 and from February to March 2012.

The impact of the treatment program was monitored through cohorts of schoolchildren and adults. Their infection status with S. mansoni and STH was determined by parasitological examinations at baseline and at annual follow-ups. The prevalence and intensity of S. mansoni and STH before and after treatment were analyzed.

Seventy-five schools were randomly assigned to one of three intervention arms: (i) annual school-based preventive chemotherapy with praziquantel (40 mg/kg) over four years; (ii) praziquantel treatment only in the first two years, followed by two years whithout treatment; and (iii) praziquantel treatment in years 1 and 3 without treatment in-between. Cross-sectional parasitologic surveys were carried out prior to each round of preventive chemotherapy. The difference in S. mansoni prevalence and infection intensity was assessed by multiple Kato-Katz thick smears, among children aged 9-12 years at the time of each survey. First-grade children, aged 5-8 years who had never received praziquantel, were also tested at baseline and at the end of the study.

ntegrated chemotherapy of neglected tropical diseases (NTD) through mass drug administration given as a single dose would increase treatment coverage and cost-effectiveness. This study reports on the safety of a combination of albendazole, ivermectin and praziquantel in the treatment of lymphatic filariasis (LF), schistosomiasis and soil-transmitted helminthiasis (STH) in infected children. In this randomised, controlled, single-blinded clinical trial conducted in 235 primary school children aged 5-18 years in Yumbe District in Northern Uganda, the triple combination therapy was compared with the current NTD programme regimen.

The objectives of this study were to determine the prevalence and distribution of distended abdomens among Ugandan school children across a range of eco-epidemiological settings and to investigate the relationship between distended abdomens and helminth infections, in particular Schistosoma mansoni, before and 1-year after anthelminthic treatment. A cross-sectional survey was conducted on 4354 school children across eight districts, with a longitudinal 1-year follow-up of 2644 children (60.7%). On both occasions, parasitological, biometrical and clinical data were collected for each child. Baseline prevalence of S. mansoni and hookworms was 44.3% and 51.8%, respectively.

This study evaluated potential changes in antischistosome immune responses in children from schools that received 4 rounds of annual mass drug administration (MDA) of praziquantel (PZQ). In a repeated cross-sectional study design, 210 schistosome egg-positive children were recruited at baseline from schools in western Kenya (baseline group). Another 251 children of the same age range were recruited from the same schools and diagnosed with schistosome infection by microscopy (post-MDA group). In-vitro schistosome-specific cytokines and plasma antibody levels were measured by ELISA and compared between the 2 groups of children.

We comparatively assessed the efficacy and tolerability of the following treatments against Schistosoma haematobium in school-aged children in Côte d'Ivoire: (i) praziquantel (40 mg/kg; standard treatment); (ii) mefloquine (25 mg/kg) combined with praziquantel (40 mg/kg); and (iii) mefloquine-artesunate (3× (100 mg artesunate +250 mg mefloquine)) combined with praziquantel (40 mg/kg) (treatments administered on subsequent days). Two urine samples were collected before, and on days 21-22 and 78-79 after the first dosing.

Pre-treatment examination and follow-up at one year post-treatment of schoolchildren aged 7, 8, and 11 years, including interview, urine examination, ultrasound examination of the urinary tract, and measurement of haemoglobin. Before treatment, the overall prevalence of S. heamatobium infection was 75.4% of the 1,642 enrolled children, and 21.8% of children excreted more than 50 eggs/10 ml urine. Prevalence increased with age. The overall prevalence of anaemia (haemoglobin <11.5 g/dl) was 61.6%, decreasing significantly with increasing age. The mean haemoglobinemia was 11 g/dl. In bivariate analysis, anaemia was significantly more frequent in children infected with S. haematobium, although it was not correlated to the intensity of infection. Anaemia was also associated with micro-haematuria and to kidney distensions. In a sub-sample of 636 children tested for P. falciparum infection, anaemia was significantly more frequent in malaria-infected children. In multivariate analysis, significant predictors of anaemia were P. falciparum infection, kidney distension, and the village.

Evaluating regression of morbidity associated with parasitic infections is an important component of community-based control programmes. We performed an intervention against Schistosoma mansoni infection, focusing on hepatosplenomegaly in the absence of periportal fibrosis, in a cohort of 67 Kenyan children aged 7-18 years from Makueni District, selected on the basis of hepatosplenomegaly detected by ultrasonography. Clinical and ultrasound examinations were conducted annually for three years after treatment, and the source of infection (a river) was regularly treated with molluscicide, thereby severely reducing exposure to schistosomiasis. Malaria transmission was uninterrupted.

A double-blind, randomized controlled trial was conducted in an endemic focus for Schistosoma mansoni in Kafr El-Sheikh Governorate, Northern Nile Delta, Egypt, to evaluate the prophylactic effect of artemether (ART) given in conjunction with praziquantel (PZQ). The study encompassed 913 primary school children randomly assigned to two treatment groups PZQ/ART and PZQ/ART-placebo. At baseline, both groups received 40 mg/kg body weight of PZQ twice four weeks apart, after which one group received 6 mg/kg body weight of ART every 3 weeks in 5 cycles during the transmission season and the other group received ART-placebo.

In March 2013, we conducted a mixed methods cross sectional study in 15 randomly selected villages. We interviewed a total of 615 respondents aged 18 years and above using semi structured questionnaires and five key informants were also purposively selected. Univariate and multivariate analysis was done. MDA uptake was defined as self reported swallowing of praziquantel during the last (2012) MDA campaign. We conducted key informant interviews with Ministry of Health, district health personnel and community health workers.

The prevalence of urinary schistosomiasis among schoolchildren in Pangani District (Tanzania) was assessed rapidly by a questionnaire approach. Based on the results, a strategy of selective treatment with praziquantel was adopted. Eleven primary schools in Mwera Division, Pangani District, with about 2500 schoolchildren were included in a control programme for urinary schistosomiasis. Macro- and microscopic haematuria diagnosed visually and with urine reagent strips was used as an indirect indicator of Schistosoma haematobium infection. Intensity of infection among children was monitored in class 5 (median age 14 years, range 11-17) by urine filtration techniques. Treatment was administered as 40 mg/kg praziquantel in a single dose at the beginning of the school year.

The objective of this research was to assess drug efficacy in school children after mass chemotherapy with praziquantel and albendazole conducted in Mwea Division, Kirinyaga District, Central Kenya in 2004. In total 2300 children aged between 4 and 18 years in five primary schools were selected for the study. Before mass chemotherapy, prevalence of infection was 47.4% for Schistosoma mansoni, 16.7% for Necator americanus, 1.6% for Ascaris lumbricoides, and 0.8% for Trichuris trichiura.

In Kemise town, where suspension of ground Endod was sprayed on the stream containing infected snails, the prevalence of the disease was reduced from 59% to 53% and the mean intensity of infection was reduced from 239 eggs per gram (EPG) of faeces to 99 EPG ($p < 0.05$). In Bati town where Endod soap approach was used, the respective reduction in the prevalence and intensity of infection was from 51% to 43% and from 195 EPG to 162 EPG ($p < 0.05$). There was also a significant reduction of the disease in the control town probably due to the effects of praziquantel treatment and other factors.

Community based cross-sectional survey was conducted in April 2019 among households with school age children (SAC) 5-14 years in seven purposively selected districts of the country. Segments to be surveyed were randomly selected and households to be interviewed from each segment were determined using systematic sampling technique. A total of 3378 households visited and 5679 SAC (5-14 years) were interviewed.

An open-label, randomized controlled trial was conducted from October 2018 to January 2019. School-aged children with a confirmed S. mansoni infection based on Kato-Katz (KK) and point-of-care circulating cathodic antigen (POC-CCA) urine cassette test were randomly assigned to receive either a single or four repeated doses of PZQ, administered at two-week intervals. The primary outcome was the difference in CR between the two treatment arms, measured by triplicate KK thick smears 10 weeks after the first treatment. Secondary outcomes included CR estimated by POC-CCA, IRR by KK and POC-CCA, and safety of repeated PZQ administration.

For 4 years, we followed 67 children enrolled in a MDA program in Kenya. Infection status and egg counts were measured each year prior to treatment. For 15 of these children, for which there was no evidence of acquired resistance, meaning they became re-infected following each treatment, we collected microsatellite genotype data from schistosomes passed in fecal samples as a representation of the force of transmission between drug treatments. We genotyped a total of 4938 parasites from these children, with an average of 329.2 parasites per child for the entire study, and an average of 82.3 parasites per child per annual examination. We compared prevalence, egg counts, and genetic measures including allelic richness, gene diversity (expected heterozygosity), adult worm burdens and effective number of breeders among time points to search for evidence for a change in transmission or schistosome populations during the MDA program.

We conducted combined in vitro PZQ efficacy testing with population genetic analyses of S. mansoni collected from children from two schools in 2010, five years after the introduction of a National Control Programme. Children at one school had received four annual PZQ treatments and the other school had received two mass treatments in total. We compared genetic differentiation, indices of genetic diversity, and estimated adult worm burden from parasites collected in 2010 with samples collected in 2005 (before the control programme began) and in 2006 (six months after the first PZQ treatment). Using 2010 larval samples, we also compared the genetic similarity of those with high and low in vitro sensitivity to PZQ.

Multiple studies and programs now find that even within well-implemented, multiyear, annual MDA programs there often remain locations that do not decline in prevalence and/or intensity to expected levels. We term such locations "persistent hotspots." To study and address persistent hotspots, investigators and neglected tropical disease (NTD) program managers need to define them based on changes in prevalence and/or intensity. But how should the data be analyzed to define a persistent hotspot? We have analyzed a dataset from an operational research study in western Tanzania after three annual MDAs using four different approaches to define persistent hotspots.

In a cluster randomized trial carried out in Jinja district, Uganda, 12 primary schools were randomized into two groups; one received education messages for schistosomiasis prevention for two months prior to mass treatment, while the other, in addition to the education messages, received a pre-treatment snack shortly before mass treatment. Four weeks after mass treatment, uptake of praziquantel was assessed among a random sample of 595 children in the snack schools and 689 children in the non-snack schools as the primary outcome. The occurrence of side effects and the prevalence and mean intensity of Schistosoma mansoni infection were determined as the secondary outcomes.

A preliminary sound knowledge of the prevailing epidemiological situations is therefore necessary to define an efficient programme to fight these infections. The extension of schistosomiasis following the installation of water resource facilities is significative of the part played by these hosts. In the hydroagricultural complex of Sourou, the prevalence of urinary schistosomiasis increased from 19% in 1954 to more than 70% in 1998-1999 in Guiédougou, the most ancient site. As to digestive schistosomiasis, almost unheard of until 1987, its prevalence ranged from 8% to 69% in 1998 in the villages located alongside the areas thus equipped.

Schistosomiasis is the second most significant parasitic disease in children in several African countries. For this purpose, the "Programme National de Lutte contre les Bilharzioses" (PNLB) was developed in partnership with the World Health Organization (WHO) to control this disease in Senegal. However, geographic isolation of Bedik ethnic groups challenged implementation of the key elements of the schistosomiasis program in eastern Senegal, and therefore, a hospital was established in Ninefescha to improve access to health care as well as laboratory support for this population.

We have designed an individually randomised, parallel group trial of intensive versus standard praziquantel (PZQ) intervention against schistosomiasis, to determine effects on vaccine response outcomes among school-going adolescents (9-17 years) from rural Schistosoma mansoni-endemic Ugandan islands. Vaccines to be studied comprise BCG on day 'zero'; yellow fever, oral typhoid and human papilloma virus (HPV) vaccines at week 4; and HPV and tetanus/diphtheria booster vaccine at week 28. The intensive arm will receive PZQ doses three times, each 2 weeks apart, before BCG immunisation, followed by a dose at week 8 and quarterly thereafter.

Endeavours to control urogenital schistosomiasis on Unguja Island (Zanzibar) have focused on school-aged children. To assess the impact of an associated health education campaign, the supervised use of the comic-strip medical booklet Juma na Kichocho by Class V pupils attending eighteen primary schools was investigated. A validated knowledge and attitudes questionnaire was completed at baseline and repeated one year later following the regular use of the booklet during the calendar year. A scoring system (ranging from 0.0 to 5.0) measured children's understandings of schistosomiasis and malaria, with the latter being a neutral comparator against specific changes for schistosomiasis. In 2006, the average score from 751 children (328 boys and 423 girls) was 2.39 for schistosomiasis and 3.03 for malaria.

Zimbabwean children aged 1-5 years (n = 104) were treated with PZQ tablets and side effects were assessed by questionnaire administered to their caregivers within 24 hours of taking PZQ. Treatment efficacy was determined 6 weeks after PZQ administration through schistosome egg counts in urine. The change in infection levels in the children 1-5 years old (n = 100) was compared to that in 6-10 year old children (n = 435).

We did a randomised controlled, parallel-group, single-blind, dose-ranging, phase 2 trial in PSAC (2-5 years) and school-aged children (SAC; aged 6-15 years) as a comparator group in southern Côte d'Ivoire. Children were randomly assigned (1:1:1:1) to 20 mg/kg, 40 mg/kg, or 60 mg/kg praziquantel or placebo. Participants, investigators, and laboratory technicians were masked to group assignment, while the investigator providing treatment was aware of the treatment group. The primary objective was to estimate the nature of the dose-response relation in terms of cure rate using the Kato Katz technique. Dose-response curves were estimated using Emax models. Available case analysis was done including all participants with primary endpoint data. This trial is registered with International Standard Randomised Controlled Trial, number ISRCTN15280205.

To improve health education within primary schools, the health education booklet "Juma na kichocho" was evaluated during a study within 5 schools using key-informant questionnaires that recorded children's knowledge and attitude (KA) towards schistosomiasis before and after daily structured-use of booklets. A total of 229 schoolchildren (114 boys : 115 girls) of between 11 and 15 years of age were interviewed and re-assessed after a working school week. Existing and putative booklet-induced changes in KA scores for schistosomiasis were compared directly against equivalent KA scores for malaria.

The objective of this study was to assess the effect of health education in the control of bilharziasis, as a part of an investigation on anti-urinary bilharziasis campaign in Niger. We carried out a survey in two groups of endemic villages on the Niger, one group of villages where there are health education campaigns (target villages) and a control village (no education campaign). Five hundred and seventy-seven people were interviewed in the area. The bilharziasis project has been the main source of information on bilharziasis of people in the project zone. The awareness of measures to fight against bilharziasis has been moderate. Indeed, 46.6% of people interrogated in the project area couldn't cite any means for containing bilharziasis. In the area of intervention, 41.5% of people interrogated were unaware of the intervention of an intermediate host in the transmission of the urinary bilharziasis.

Two hundred and seventy-five children from six schools who rejected praziquantel treatment were divided into intervention and control groups. Before the intervention, preassessment interviews were conducted on their knowledge about praziquantel treatment and schistosomiasis. The Schisto and Ladders version 2 game as an intervention, and the Snakes and Ladders game as a control, were played for 6 mo. Postassessment interviews, including focus group discussions, were conducted.

Kabuyaya, M., Chimbari, M. J., & Mukaratirwa, S. (2018). Infection status and risk factors associated with urinary schistosomiasis among school-going children in the Ndumo area of uMkhanyakude District in KwaZulu-Natal, South Africa two years post-treatment. International journal of infectious diseases : IJID : official publication of the International Society for Infectious Diseases, 71, 100–106. https://doi.org/10.1016/j.ijid.2018.04.002

Coulibaly, J. T., Panic, G., Yapi, R. B., Kovač, J., Barda, B., N'Gbesso, Y. K., Hattendorf, J., & Keiser, J. (2018). Efficacy and safety of ascending doses of praziquantel against Schistosoma haematobium infection in preschool-aged and school-aged children: a single-blind randomised controlled trial. BMC medicine, 16(1), 81.

Adriko, M., Tinkitina, B., Tukahebw, E. M., Standley, C. J., Stothard, J. R., & Kabatereine, N. B. (2018). The epidemiology of schistosomiasis in Lango region Uganda 60 years after Schwetz 1951: Can schistosomiasis be eliminated through mass drug administration without other supportive control measures?. Acta tropica, 185, 412–418. https://doi.org/10.1016/j.actatropica.2018.06.009

Bocanegra, C., Pintar, Z., Mendioroz, J., Serres, X., Gallego, S., Nindia, A., Aznar, M. L., Soriano-Arandes, A., Salvador, F., Gil, E., Sikaleta, N., Moreno, M., & Molina, I. (2018). Ultrasound Evolution of Pediatric Urinary Schistosomiasis after Treatment with Praziquantel in a Highly Endemic Area. The American journal of tropical medicine and hygiene, 99(4), 1011–1017. https://doi.org/10.4269/ajtmh.18-0343

Haggag, A. A., Rabiee, A., Abd Elaziz, K. M., Gabrielli, A. F., Abdelhai, R., Hashish, A., Jabbour, J., & Ramzy, R. M. R. (2018). Elimination of schistosomiasis haematobia as a public health problem in five governorates in Upper Egypt. Acta tropica, 188, 9–15. https://doi.org/10.1016/j.actatropica.2018.08.024

Mutsaka-Makuvaza, M. J., Matsena-Zingoni, Z., Tshuma, C., Ray, S., Zhou, X. N., Webster, B., & Midzi, N. (2018). Reinfection of urogenital schistosomiasis in pre-school children in a highly endemic district in Northern Zimbabwe: a 12 months compliance study. Infectious diseases of poverty, 7(1), 102. https://doi.org/10.1186/s40249-018-0483-7

Sheehy, C., Lawson, H., Andriamasy, E. H., Russell, H. J., Reid, A., Raderalazasoa, G. U., Dodge, G., Kornitschky, R., Penney, J. M. S., Ranaivoson, T. N., Andrianiaina, A., Emmanoela, J. S., Bustinduy, A. L., Stothard, J. R., Andrianjaka, L., & Spencer, S. A. (2021). Prevalence of intestinal schistosomiasis in pre-school aged children: a pilot survey in Marolambo District, Madagascar. Infectious diseases of poverty, 10(1), 87. https://doi.org/10.1186/s40249-021-00871-y

Lund, A. J., Sam, M. M., Sy, A. B., Sow, O. W., Ali, S., Sokolow, S. H., Bereknyei Merrell, S., Bruce, J., Jouanard, N., Senghor, S., Riveau, G., Lopez-Carr, D., & De Leo, G. A. (2019). Unavoidable Risks: Local Perspectives on Water Contact Behavior and Implications for Schistosomiasis Control in an Agricultural Region of Northern Senegal. The American journal of tropical medicine and hygiene, 101(4), 837–847. https://doi.org/10.4269/ajtmh.19-0099

Nkurunungi, G., Zirimenya, L., Nassuuna, J., Natukunda, A., Kabuubi, P. N., Niwagaba, E., Oduru, G., Kabami, G., Amongin, R., Mutebe, A., Namutebi, M., Zziwa, C., Amongi, S., Ninsiima, C., Onen, C., Akello, F., Sewankambo, M., Kiwanuka, S., Kizindo, R., Kaweesa, J., … POPVAC trial team principal investigator (2021). Effect of intensive treatment for schistosomiasis on immune responses to vaccines among rural Ugandan island adolescents: randomised controlled trial protocol A for the 'POPulation differences in VACcine responses' (POPVAC) programme. BMJ open, 11(2), e040426. https://doi.org/10.1136/bmjopen-2020-040426

Olliaro, P. L., Vaillant, M., Hayes, D. J., Montresor, A., & Chitsulo, L. (2013). Practical dosing of praziquantel for schistosomiasis in preschool-aged children. Tropical medicine & international health : TM & IH, 18(9), 1085–1089. https://doi.org/10.1111/tmi.12152

Sircar, A. D., Mwinzi, P. N. M., Onkanga, I. O., Wiegand, R. E., Montgomery, S. P., & Secor, W. E. (2018). Schistosoma mansoni Mass Drug Administration Regimens and Their Effect on Morbidity among Schoolchildren over a 5-Year Period-Kenya, 2010-2015. The American journal of tropical medicine and hygiene, 99(2), 362–369. https://doi.org/10.4269/ajtmh.18-0067

Osakunor, D. N. M., Woolhouse, M. E. J., & Mutapi, F. (2018). Paediatric schistosomiasis: What we know and what we need to know. PLoS neglected tropical diseases, 12(2), e0006144. https://doi.org/10.1371/journal.pntd.0006144

Maïga, F. K., Sangare, M., Dolo, H., Dicko, I., Diabate, A. F., Keita, M., Diarra, L., Soumaoro, L., Thera, S., Diallo, O., Guindo, I., Traoré, M., Faye, O., Doumbia, S., & Coulibaly, Y. I. (2022). Knowledge and factors influencing schistosomiasis control interventions in the hyperendemic health district of Kalabancoro in Mali, 2020. The Pan African medical journal, 43, 48. https://doi.org/10.11604/pamj.2022.43.48.30512

Sturrock R. F. (2001). Schistosomiasis epidemiology and control: how did we get here and where should we go?. Memorias do Instituto Oswaldo Cruz, 96 Suppl, 17–27. https://doi.org/10.1590/s0074-02762001000900003

Ekpo, U. F., Oluwole, A. S., Abe, E. M., Etta, H. E., Olamiju, F., & Mafiana, C. F. (2012). Schistosomiasis in infants and pre-school-aged children in sub-Saharan Africa: implication for control. Parasitology, 139(7), 835–841. https://doi.org/10.1017/S0031182012000029

Kabatende, J., Barry, A., Mugisha, M., Ntirenganya, L., Bergman, U., Bienvenu, E., & Aklillu, E. (2022). Safety of Praziquantel and Albendazole Coadministration for the Control and Elimination of Schistosomiasis and Soil-Transmitted Helminths Among Children in Rwanda: An Active Surveillance Study. Drug safety, 45(8), 909–922. https://doi.org/10.1007/s40264-022-01201-3

Massa, K., Olsen, A., Sheshe, A., Ntakamulenga, R., Ndawi, B., & Magnussen, P. (2009). Can coverage of schistosomiasis and soil transmitted helminthiasis control programmes targeting school-aged children be improved? New approaches. Parasitology, 136(13), 1781–1788. https://doi.org/10.1017/S0031182008000474

Kura, K., Hardwick, R. J., Truscott, J. E., & Anderson, R. M. (2021). What is the impact of acquired immunity on the transmission of schistosomiasis and the efficacy of current and planned mass drug administration programmes?. PLoS neglected tropical diseases, 15(12), e0009946. https://doi.org/10.1371/journal.pntd.0009946

Byrne, A., Rosário, A., da Conceição Ferreira, M., de Jesus Trovoada Dos Santos, M., Rollinson, D., & Vaz Nery, S. (2022). Progress towards control and elimination of neglected tropical diseases targeted by preventive chemotherapy in São Tomé e Príncipe. Transactions of the Royal Society of Tropical Medicine and Hygiene, 116(5), 446–453. https://doi.org/10.1093/trstmh/trab153

Fenwick, A., Webster, J. P., Bosque-Oliva, E., Blair, L., Fleming, F. M., Zhang, Y., Garba, A., Stothard, J. R., Gabrielli, A. F., Clements, A. C., Kabatereine, N. B., Toure, S., Dembele, R., Nyandindi, U., Mwansa, J., & Koukounari, A. (2009). The Schistosomiasis Control Initiative (SCI): rationale, development and implementation from 2002-2008. Parasitology, 136(13), 1719–1730. https://doi.org/10.1017/S0031182009990400

Ezeamama, A. E., He, C. L., Shen, Y., Yin, X. P., Binder, S. C., Campbell, C. H., Jr, Rathbun, S., Whalen, C. C., N'Goran, E. K., Utzinger, J., Olsen, A., Magnussen, P., Kinung'hi, S., Fenwick, A., Phillips, A., Ferro, J., Karanja, D. M., Mwinzi, P. N., Montgomery, S., Secor, W. E., … Colley, D. G. (2016). Gaining and sustaining schistosomiasis control: study protocol and baseline data prior to different treatment strategies in five African countries. BMC infectious diseases, 16, 229. https://doi.org/10.1186/s12879-016-1575-2

Garba, A., Touré, S., Dembelé, R., Boisier, P., Tohon, Z., Bosqué-Oliva, E., Koukounari, A., & Fenwick, A. (2009). Present and future schistosomiasis control activities with support from the Schistosomiasis Control Initiative in West Africa. Parasitology, 136(13), 1731–1737. https://doi.org/10.1017/S0031182009990369

Allam, A. F., Salem, A., Elsheredy, A., Dewair, M. M., Ibrahim, H. S., Farag, H. F., Hagras, N. A., & Shehab, A. Y. (2021). Intestinal schistosomiasis among preschool and school-aged children in a rural setting near Alexandria: initiative for elimination. Tropical medicine & international health : TM & IH, 26(6), 632–639. https://doi.org/10.1111/tmi.13562
Secor WE, Wiegand RE, Montgomery SP, Karanja DMS, Odiere MR. Comparison of School-Based and Community-Wide Mass Drug Administration for Schistosomiasis Control in an Area of Western Kenya with High Initial Schistosoma mansoniInfection Prevalence: A Cluster Randomized Trial. Am J Trop Med Hyg. 2020 Feb;102(2):318-327. doi: 10.4269/ajtmh.19-0626. PMID: 31802733; PMCID: PMC7008345.
Colley DG; Fleming FM; Matendechero SH; Knopp S; Rollinson D; Utzinger J; Castleman JD; Kittur N; King CH; Campbell CH; Kabole FM; Kinung'hi S; Ramzy RMR; Binder S, The American journal of tropical medicine and hygiene [Am J Trop Med Hyg], ISSN: 1476-1645, 2020 Jul; Vol. 103 (1_Suppl), pp. 125-134; Publisher: American Society of Tropical

Lund AJ; Sam MM; Sy AB; Sow OW; Ali S; Sokolow SH; Bereknyei Merrell S; Bruce J; Jouanard N; Senghor S; Riveau G; Lopez-Carr D; De Leo GA, The American journal of tropical medicine and hygiene [Am J Trop Med Hyg], ISSN: 1476-1645, 2019 Oct; Vol. 101 (4), pp. 837-847; Publisher: American Society of Tropical Medicine and Hygiene; PMID: 31452497;

Cribb DM; Clarke NE; Doi SAR; Vaz Nery S, PLoS neglected tropical diseases [PLoS Negl Trop Dis], ISSN: 1935-2735, 2019 Oct 11; Vol. 13 (10), pp. e0007808; Publisher: Public Library of Science; PMID: 31603895;

Gurarie D; Lo NC; Ndeffo-Mbah ML; Durham DP; King CH, PLoS neglected tropical diseases [PLoS Negl Trop Dis], ISSN: 1935-2735, 2018 May 21; Vol. 12 (5), pp. e0006514; Publisher: Public Library of Science; PMID: 29782500;

Person B; Knopp S; Ali SM; A'kadir FM; Khamis AN; Ali JN; Lymo JH; Mohammed KA; Rollinson D, Journal of biosocial science [J Biosoc Sci], ISSN: 1469-7599, 2016 Sep; Vol. 48 Suppl 1, pp. S56-73; Publisher: Cambridge University Press; PMID: 27428066;

Campbell CH Jr; Rathbun S; Whalen CC; N'Goran EK; Utzinger J; Olsen A; Magnussen P; Kinung'hi S; Fenwick A; Phillips A; Ferro J; Karanja DM; Mwinzi PN; Montgomery S; Secor WE; Hamidou A; Garba A; King CH; Colley DG, BMC infectious diseases [BMC Infect Dis], ISSN: 1471-2334, 2016 May 26; Vol. 16, pp. 229; Publisher: BioMed Central; PMID: 27230666;

Muhumuza S; Olsen A; Katahoire A; Nuwaha F, BMC infectious diseases [BMC Infect Dis], ISSN: 1471-2334, 2015 Oct 14; Vol. 15, pp. 423; Publisher: BioMed Central; PMID: 26466681;

Garba A; Touré S; Dembelé R; Boisier P; Tohon Z; Bosqué-Oliva E; Koukounari A; Fenwick A, Parasitology [Parasitology], ISSN: 1469-8161, 2009 Nov; Vol. 136 (13), pp. 1731-7; Publisher: Cambridge University Press; PMID: 19631007;

Gutman J; Richards FO Jr; Eigege A; Umaru J; Alphonsus K; Miri ES, Annals of tropical medicine and parasitology [Ann Trop Med Parasitol], ISSN: 1364-8594, 2009 Sep; Vol. 103 (6), pp. 501-11; Publisher: Taylor & Francis; PMID: 19695155;

de Vlas SJ; Danso-Appiah A; van der Werf MJ; Bosompem KM; Habbema JD, Tropical medicine & international health : TM & IH [Trop Med Int Health], ISSN: 1360-2276, 2004 Jun; Vol. 9 (6), pp. A16-21; Publisher: Blackwell Scientific Publications; PMID: 15189470;

Taylor M, Bulletin of the World Health Organization [Bull World Health Organ], ISSN: 1564-0604, 2008 Oct; Vol. 86 (10), pp. 738; Publisher: World Health Organization; PMID: 18949203, Database: MEDLINE

Adeneye AK; Akinwale OP; Idowu ET; Adewale B; Manafa OU; Sulyman MA; Omotola BD; Akande DO; Mafe MA; Appelt B, Research in social & administrative pharmacy : RSAP [Res Social Adm Pharm], ISSN: 1551-7411, 2007 Jun; Vol. 3 (2), pp. 183-98; Publisher: Elsevier; PMID: 17561219

Fenwick A; Webster JP; Bosque-Oliva E; Blair L; Fleming FM; Zhang Y; Garba A; Stothard JR; Gabrielli AF; Clements AC; Kabatereine NB; Toure S; Dembele R; Nyandindi U; Mwansa J; Koukounari A, Parasitology [Parasitology], ISSN: 1469-8161, 2009 Nov; Vol. 136 (13), pp. 1719-30; Publisher: Cambridge University Press; PMID: 19631008;

[illegible] Homeida M; Kabatereine N; Kabole FM; King CH; Mafe MA; Midzi N; Mutapi F; Mwanga JR; Ramzy RMR; Satrija F; Stothard JR; Traoré MS; Webster JP; Utzinger J; Zhou XN; Danso-Appiah A; Eusebi P; Loker ES; Obonyo CO; Quansah R; Liang S; Vaillant M; Murad MH; Hagan P; Garba A, The Lancet.

Kabatende J; Barry A; Mugisha M; Ntirenganya L; Bergman U; Bienvenu E; Aklillu E, Drug safety [Drug Saf], ISSN: 1179-1942, 2022 Aug; Vol. 45 (8), pp. 909-922; Publisher: Adis, Springer International; PMID: 35819751;

Mushi V; Zacharia A; Shao M; Mubi M; Tarimo D, PloS one [PLoS One], ISSN: 1932-6203, 2022 Feb 15; Vol. 17 (2), pp. e0263929; Publisher: Public Library of Science; PMID: 35167622;

Ouattara M; Diakité NR; Yao PK; Saric J; Coulibaly JT; Assaré RK; Bassa FK; Koné N; Guindo-Coulibaly N; Hattendorf J; Utzinger J; N'Goran EK, PLoS neglected tropical diseases [PLoS Negl Trop Dis], ISSN: 1935-2735, 2021 Jan 15; Vol. 15 (1), pp. e0008845; Publisher: Public Library of Science; PMID: 33449924;

Mnkugwe RH; Minzi O; Kinung'hi S; Kamuhabwa A; Aklillu E, PLoS neglected tropical diseases [PLoS Negl Trop Dis], ISSN: 1935-2735, 2020 Sep 23; Vol. 14 (9), pp. e0008619; Publisher: Public Library of Science; PMID: 32966290;

Kinung'hi SM; Olsen A; Magnussen P; Karanja DMS; Mwinzi PNM; Montgomery SP; Secor WE; Phillips AE; Dhanani N; Gazzinelli-Guimaraes PH; Clements MN; N'Goran EK; Meite A; Utzinger J; Hamidou AA; Garba A; Fleming FM; Whalen CC; King CH; Colley DG, The American journal of

By: Colley DG; Fleming FM; Matendechero SH; Knopp S; Rollinson D; Utzinger J; Castleman JD; Kittur N; King CH; Campbell CH; Kabole FM; Kinung'hi S; Ramzy RMR; Binder S, The American journal of tropical medicine and hygiene [Am J Trop Med Hyg], ISSN: 1476-1645, 2020 Jul; Vol. 103 (1_Suppl), pp. 125-134; Publisher: American Society of Tropical Medicine and Hygiene; PMID: 32400345

Schistosomiasis, a disease caused by blood flukes of the genus Schistosoma, belongs to the neglected tropical diseases. Left untreated, schistosomiasis can lead to severe health problems and even death. An estimated 800 million people are at risk of schistosomiasis and 250 million people are infected. The global strategy to control and eliminate schistosomiasisemphasizes large-scale preventive chemotherapy with praziquantel targeting school-age children. Other tools are available, such as

Shen Y; Sung MH; King CH; Binder S; Kittur N; Whalen CC; Colley DG, The Journal of infectious diseases [J Infect Dis], ISSN: 1537-6613, 2020 Feb 18; Vol. 221 (5), pp. 796-803; Publisher: Oxford University Press; PMID: 31621850;

Secor WE; Wiegand RE; Montgomery SP; Karanja DMS; Odiere MR, The American journal of tropical medicine and hygiene [Am J Trop Med Hyg], ISSN: 1476-1645, 2020 Feb; Vol. 102 (2), pp. 318-327; Publisher: American Society of Tropical Medicine and Hygiene; PMID: 31802733;

Chisango TJ; Ndlovu B; Vengesai A; Nhidza AF; Sibanda EP; Zhou D; Mutapi F; Mduluza T, BMC infectious diseases [BMC Infect Dis], ISSN: 1471-2334, 2019 Mar 04; Vol. 19 (1), pp. 219; Publisher: BioMed Central; PMID: 30832614;

Atalabi TE; Adubi TO, BMC infectious diseases [BMC Infect Dis], ISSN: 1471-2334, 2019 Jan 18; Vol. 19 (1), pp. 73; Publisher: BioMed Central; PMID: 30658583;

Results	findings
Prevalence of STH was 15.5% for any STH species, 9.47% for Ascaris lumbricoides, 1.78% for Trichuris trichiura, and 7.24% for hookworm. Intestinal schistosomiasis (Schistosoma mansoni) infection prevalence was 0.85% by Kato Katz, 21.6% by POC-CCA trace positive (Tr +), and 13.3% trace negative (Tr-). Microhaematuria was 2.77%, with 0.13% of people examined with S. haematobium eggs detected by urine filtration. At the household level, increased (> 30 min) time taken to collect drinking water, sharing a latrine, and lack of handwashing facilities were all associated with a greater risk of A. lumbricoides, hookworm, and S. mansoni infection. Not disposing of infant stool at the household and	The principal finding of this study is that lack of access to WaSH, such as improved drinking water and shared toilet and hand-washing facilities, were linked to an increased risk of infection with STH and schistosome parasites. These associations are difficult to establish at an individual household level because of wide variability in access between houses but are detectable when coverage is aggregated at the community level. Maintenance of WaSH facilities as well as increased access within the whole community is important in influencing the community-wide prevalence of infection with STH and
WASH and parasitology data were available for 1,645 schools. More frequent collection of water for schools, from open freshwater sources was associated with statistically significantly higher Schistosoma mansoni infection intensity (Kendall's τb = 0.097, 95% confidence interval, CI: 0.011 to 0.18), better sanitation was associated with significantly lower Ascaris lumbricoides intensity (Kendall's τb = -0.067, 95% CI: -0.11 to -0.023) and borderline significant lower hookworm	Improving school WASH may reduce transmission of these parasites. However, different forms of WASH appear to have different effects on infection with the various parasites, with our analysis finding the strongest associations between water and S. mansoni, sanitation and A. lumbricoides, and hygiene and hookworm.
Data of 8002 stool tests was extracted. The proportion of S. mansoni progressively decreased from 9.6% in 2013 to 4.1% in 2018 in the overall patient population and from 20.3% in 2013 to 8.8% in 2018 in school-aged children. However, a declining trend of S. mansoni was observed before	The declined trend of S. mansoni positivity rate is encouraging and may be related to the existence of intervention packages. Although the timing of MDA was related with low positivity rate of S. mansoni infection, it has not resulted in the expected

At the baseline, prevalence was low among children from the village of Mbane who live close to the Lac de Guiers (38%), moderate among those from the villages of Dioundou and Khodit, which neighbor the Doue river (46%), and very high at Khodit (90.6%) and Guia (91.2%) which mainly use an irrigation canal. After treatment, the observed cure rates confirmed the efficacy of praziquantel. The lowest cure rate (88.5%) was obtained in the village using the irrigation canal, while high cure rates were obtained in those using the lake (96.5%) and the river (98%). However, high egg reduction rates (between 96.7 and 99.7%) were obtained in all the villages. The re-infection was significantly higher in the village using the canal (42.5%) than in the villages accessing the Lac de Guiers (18.3%) and the Doue river (14.8%).

Praziquantel has an impact on reducing the prevalence and intensity of urogenital schistosomiasis. However, in the Senegal river basin, S. haematobium remains a real health problem for children living in the villages near the irrigation canals, despite regular treatment, while prevalence is declining from those frequenting the river and the Lac de Guiers. Trial registration ClinicalTrials.gov, NCT04635553. Registered 19 November 2020 retrospectively registered, https://www.

The overall prevalence of S. mansoni infection among the schoolchildren was 52.1% with a mean intensity of 546 eggs per gram of stool. Majorities of the S. mansoni infections were moderate to heavy intensity, with only 5.0% light infections. Praziquantel administered at a single oral dose of 40 mg/kg achieved a cure rate of 91.7% and reduced the egg rate by 86.8%. Twenty-one schoolchildren remained infected at 4 weeks post-treatment, among which 6 and 15 children had moderate and light infections, respectively.

S. mansoni prevalence among primary school children in Northeast Ethiopia was high, highlighting the need to implement school-based chemotherapy with annual frequency. The efficacy of praziquantel at 40 mg/kg is sufficient to permit continued use in treating S. mansoni-infected schoolchildren.

The overall prevalence for S. haematobium and S. mansoni at baseline was 28.5% and 0.4%, respectively. At follow-up survey after 6-9 months post-treatment, the prevalence of S. haematobium infection was reduced to 13.5% (95% CI = 0.331-0.462). A higher reduction in prevalence was observed among girls, those with moderately infected status (around 20%), and residents in rural areas, than among boys, those with high prevalence (>40%), and residents in urban areas. After health education, increased awareness about schistosomiasis was checked by questionnaire survey. Also, a drinking water facility was constructed at Al Hidaib village, where infection rate was reduced more compared to that in a neighboring village within the same unit. However, we found no significant change in the prevalence of S. mansoni infection between baseline and follow-up survey (95% CI = 0.933-6.891).

At the end of the project, the prevalence of S. haematobium infection was reduced by more than 50% in comparison with the baseline rate. Approximately 200,000 subjects had received either praziquantel therapy, health education, or supply of clean water. To consolidate the achievements of this project, the integrated intervention should be adapted continuously.

The MDA program only partially controlled parasite infections, owing to high rates of re-infection. The infection rate of S. haematobium across all 10 schools, for example, was only reduced by 1.8%, and even this change not significant, even though the S. haematobiuminfection rates of the Chaani and Mzambarauni schools were significantly reduced from 64.4 and 23.4%, respectively, at the first screening, to 7.3 and 2.3% at the last screening. The overall infection rate of Ascaris lumbricoides was reduced from 36.0% at the first screening to 22.6% at the last screening.

At baseline, 47/67 (70.1%) children presented Schistosoma haematobium (75.8% in the baseline total sample) and 12/67 (17.9%) with STH (30.5% in the initial sample, p=0.010). Among the children, 47.3% had heavy Schistosoma haematobium infection. The most frequent STH was Trichuris trichiura in 9.0%. We also found Hymenolepis nana (13.2%) and Plasmodium falciparum (9.1%) infections and anaemia (82.1%). One mo after chemotherapy there was a significant (p=0.013) reduction of Schistosoma haematobium prevalence (23.5%) and a high egg reduction rate (86.9%). Considering the sample of 67 children, the mean egg concentration was 498 at baseline, 65 at 1 mo and 252 at 6 mo (p<0.05). We also observed a reduction in STH infections, 50% in Ascaris lumbricoides, 33.3% in T. trichiura and 50% in hookworms. At 6 mo, the prevalence of Schistosoma haematobium (76.1%) was similar to the baseline and the STH reduction was not significant.

Longitudinal studies have reported many losses in these settings, but we were able to show that mass drug administration for control of schistosomiasis and STH present low effectiveness, that reinfections occur rapidly and that stand alone anthelmintic therapy is not a sustainable choice.

Five rounds of MDA have been implemented from 2009 along with PHAST and improved water supply with pumped wells as other control strategies for complementing MDA. A remarkable steady decline of schistosomiasis and STHs was observed from 2009 to 2012 with significant trends in their prevalence decline, and thereafter infection rate has remained at a low sustainable control. By the third follow-up survey in 2012, Schistosoma mansoni infection prevalence was reduced by 90.5% and hookworm by 93.3% among schoolchildren while in adults the corresponding reduction was 83.2% and 56.9%, respectively.

Integrated control strategies have successfully reduced S. mansoni and STH infection status to a lower level. This study further suggests that monitoring and evaluation is a crucial component of any large-scale STH and schistosomiasis intervention.

The overall prevalence of both STH and schistosomiasis declined significantly, from 31.5% to 11.6% for STH and from 23.5% to 5.0% for schistosomiasis ($p<0.001$ in both instances). Egg counts from both years were available only for hookworm and S. mansoni; intensity of infection decreased significantly for both infections from 2009 to 2015 ($p<0.001$ for both infections). In areas with high baseline prevalence, rebound of hookworm infection was noted in children who had not received albendazole in the past 6 months.	After four to five years of MDA in Togo, th
At baseline, higher intensities of S. haematobium infection were observed in children with anemia and/or severe microhematuria, but there was no apparent association between the risk of undernutrition and intensity of S. haematobium infection. Significant reductions in the prevalence and intensity of S. haematobium infection 1 year after treatment were, however, observed. Children who benefited the most from anthelmintic treatment in terms of increased hemoglobin concentrations were those who had anemia at baseline and those with highly positive microhematuria scores at baseline.	This study suggests that even a single round of mass chemotherapy can have a substantial impact on S. haematobium infection and its associated morbidity in children.
Logistic regression was used to test association between dependent and independent variables. We found MDA uptake among PSC as 72.5%, and the prevalence of Schistosoma haematobium infection 5.8%. The risk of infection increased among PSC engaged in fetching water and adjusted odds ratio (AOR) for swimming, bathing, fishing, crossing ponds and paddy fields were 0.123, 0.166, 0.232, 0.202 and 0.093 respectively. Thus we conclude that multiple water contact activities and low participation in MDA is responsible for persistent Schistosoma transmission.	

Improvements were observed in responses to all questions between pre- and post-education answers in 2017 (53-77%, P < 0.0001) and 2018 (72-98%, P < 0.0001) and in the pre-education answers between years (53-72%, P < 0.0001). Praziquantel mass drug administration attendance improved, rising from 64% to 91% (P < 0.0001), alongside improved latrine use, from 89% to 96% (P = 0.005). This community-consulted and -engaged SEP resulted in substantial improvements in children's understanding of schistosomiasis, with improvements in praziquantel uptake and latrine use. Socioculturally tailored education programs can help gain schistosomiasis control. Continued investment in SEP will help promote the future well-being of children through increased participation in control and treatment

At year 5, neither overall S. mansoni prevalence nor the prevalence of high infection-intensity S. mansoniinfection was significantly reduced compared with baseline in either the CWT cohort (N = 277 remaining) or the SBT cohort (N = 235 remaining). Nevertheless, by year 5, children in both cohorts demonstrated significant decreases in wasting, ultrasound-detected organomegaly, and STH infection along with significantly improved pediatric quality-of-life scores compared with year 1. Stunting did not change over time, but children who were S. mansoni egg-positive at year 5 had significantly more stunting than children without schistosomiasis. The only significant difference between arms at year 5 was a lower prevalence of STH infections in the CWT group.

S. haematobium was detected in 287/3514 school children (adjusted prevalence: 8.76%, range across sentinel sites: 0.0-56.3%; median: 2.5%). The prevalence of S. haematobium infection was higher in the children from the Centre-Est, Est and Sahel regions than in those from Burkina Faso's other eight regions with sentinel sites ($P < 0.001$). The adjusted arithmetic mean intensity of S. haematobium infection, among all children, was 6.0 eggs per 10 ml urine. Less than 1% of the children in six regions had heavy S. haematobium infections - i.e. at least 50 eggs per 10 ml urine - but such infections were detected in 8.75% (28/320) and 11.56% (37/320) of the children from the Centre-Est and Sahel regions, respectively. Schistosoma mansoni was only detected in two regions and 43 children - i.e. 1 (0.31%) of the 320 from Centre-Sud and 42 (8.75%) of the 480 from Hauts Bassins.

The reduction of Bulinus globosus snail populations and S. haematobium-infected snails will be investigated. In 15 other communities, interventions triggering behaviour change have been designed and will be implemented in collaboration with the community. A change in knowledge, attitudes and practices will be assessed annually through focus group discussions and in-depth interviews with schoolchildren, teachers, parents and community leaders. In all 45 communities, changes in the health system, water and sanitation infrastructure will be annually tracked by standardized questionnaire-interviews with community leaders. Additional issues potentially impacting on study outcomes and all incurring costs will be recordedand monitored longitudinally.

Elimination of schistosomiasis has become a priority on the agenda of the Zanzibar government and the international community. Our study will contribute to identifying what, in addition to preventive chemotherapy, needs to be done to prevent, control, and ultimately eliminate schistosomiasis, and to draw lessons for current and future schistosomiasis elimination programmes in Africa and elsewhere.

Overall, mean infection prevalence was 7.4% (95%CI: 7.0-7.7, 1514/20,389) and geometric mean infection intensity was 15.8eggs/10mls. Both infection prevalence (5.9% versus 9%, P<0.001) and intensity (t = -6.9256, P<0.001) were significantly higher in males compared to females respectively. Light and heavy infections were detected in 82.3% and 17.7% of the positive children respectively. The prevalence of macrohaematuria was 0.3% and that of microhaematuria was 9.3% (95%CI:8.9-9.7). The sensitivity and specificity of the urine reagent strip were 78% (95%CI: 76.1-79.9) and 99.8% (95%CI: 99.7-99.9). Having light (P<0.001) and heavy infection intensities (P<0.001) and living in the study districts increased the odd of having microhaematuria. Predictors of S. haematobium infection were being male (P<0.003), microhaematuria (P<0.001), and living in the three study districts (P<0.001) compared to living at Nzega district.

The findings provide an updated geographical prevalence which gives an insight on the planning and implementation of MDA. Comparing with the earlier mapping survey at the start of the national wide mass drug administration, the prevalence of S. haematobium infection have significantly declined. This partly could be attributed to repeated rounds of mass drug administration. The urine reagent strips remain as a useful adjunct diagnostic test for rapid monitoring of urogenital schistosomiasis in areas with low and high prevalence. Based on prevalence levels and with some schools having no detectable infections, review of the current blanket mass drug administration is recommended.

A total of 1451 students, 708 from intervention and 743 from non-intervention schools completed the questionnaire. Noting some between island differences, students who had received the HEBC interventions reported significant improvements in knowledge about Schistosoma haematobium transmission and personal risk, strategies for schistosomiasis prevention, and self-reported changes in risk behaviours: stopped washing laundry/dishes 49.4% (350/708) versus 5.8% (43/743), stopped bathing in streams/ponds 49.4% (350/708) versus 4.2% (31/743), and stopped playing in streams/ponds 40.8% (289/708) versus 10.8% (80/743). HEBC exposed children also reported a significant increase in swallowing tablets during mass drug administration (MDA) campaigns (when they had not before) 30.2% (214/708) versus 4.6% (34/743).

The school based HEBC interventions were associated with desirable positive behaviour change among students. Data suggest that scaling up HEBC interventions to all schools in high-risk areas, augmented with bi-annual MDA, can help to reduce prevalence of urogenital schistosomiasis in Zanzibar, strengthening the possibility for future disease elimination.

The reported treatment seeking and preventive practices were congruous with the actual (observed) behaviour. Frequency, duration and timing of water contacts also decreased significantly after the intervention and took into consideration the fact that those activities which need larger body surface exposure, for a long period and at an appropriate time when cercarial densities are high (i.e. around noon) are important for the transmission of schistosomiasis. We conclude that PHAST intervention has succeeded in effecting positive changes in peoples' perceptions and attitudes towards water. As a result, knowledge obtained from the said intervention was translated into actions to prevent schistosomiasis. Studies on knowledge, attitudes and practices coupled with structured observations should be part of the integrated approach for the control of schistosomiasis.

In adults, the apparent S. haematobium prevalence was 3.9% in 2011 and 0.4% in 2020. In schoolchildren, the prevalence decreased from 6.6% in 2012 to 1.2% in 2019 with vicissitudes over the years. Prominent recrudescence of infection from 2.8% in 2019 to 9.1% (+225%) in 2020 was observed in 29 schools with historically moderate prevalences (≥10%). Compared with 2019, reinfection in 2020 was particularly striking in boys aged 9-16 years. Being male was a risk factor for infection in 2020 (adults: odds ratio (OR): 6.24, 95% confidence interval (95% CI): 1.96-19.60; schoolchildren: OR: 2.06, 95% CI: 1.52-2.78). Living near to a natural freshwater body significantly increased the odds of infection in adults (OR: 2.90, CI: 1.12-7.54).

After 11 rounds of MDA over 7 years and a 16-month treatment gap, the urogenital schistosomiasis prevalence considerably rebounded in hotspot areas. Future elimination efforts in Zanzibar should focus on re-intensifying MDA plus additional interventions in hotspot areas. In low-prevalence areas, the strategy might be adapted from MDA to targeted surveillance-response.

Treatment significantly decreased the prevalence of S. mansoni and associated morbidity in the treated groups. The untreated preschool children also showed a significant decrease in the prevalence of S. mansoni, from 21.1% (2008) to 6.3% (2010) ($p<0.001$). The percentage of untreated schoolchildren with a normal portal vein score increased significantly from 57.8% (2008) to 70.3% (2010) ($p=0.029$).	The significantly lower rates of S. mansoni and the decreased liver morbidity in untreated preschool children in 2010 suggest decreased environmental transmission rates and improved liver morbidity in untreated children following several rounds of mass drug administration.
CR after the first dose of PZQ was 66.7%, increased to 79.12% after second dose (X2 = 3.05, P = 0.08). Median egg count before treatment was 30.00 (6.00-744), that significantly decreased after two doses of PZQ to 0.00 (0.00-221.33) (Z = 8.29, P = 0.001). Children aged 10-15 years showed higher CR (91.3%) than those aged 5-9 years (OR = 5.25, CI 1.58-17.40).	PZQ is still an effective agent against S. mansoni in endemic areas, achieving a high CR and ERR with predominantly low intensity of infection. Age is a main predictor of response to PZQ.
The overall prevalence (and intensity) in these sentinel sites pre-MDA of S. mansoni was 69.0% (170.8 epg), hookworm: 41.7% (71.7 epg), Ascaris lumbricoides: 1.8% and Trichuris trichiura: 3.8%. Six months post MDA, the findings were S. mansoni: 38.2% (47.3 epg) and hookworm: 14.5% (8.7 epg), representing a reduction from pre-MDA levels of 44.6% (65.2%) and 72.3% (87.9%) respectively. The proportion of children who were moderately or heavily infected with S. mansoni fell from 35.6% pre MDA to 9.9% post MDA.	Significant reduction in S. mansoni and hookworm infection was achieved by this first round MDA in school-going children in Sierra Leone. This reduction in infection burden can potentially contribute to a reduction of morbidity, such as anaemia, in these children.

In total, data were collected from 167,500 individuals across 225 villages in nine districts within the Niger River valley, Western Niger. Overall, the prevalence of S. haematobium decreased from baseline to Year 5 across all study arms. The relative reduction of prevalence was greater in biannual compared with annual treatment across all arms; however, the only significant difference was seen in areas with a high starting prevalence. Although adults were not targeted for treatment in SBT arms, a statistically significant decrease in prevalence among adults was seen in moderate prevalence areas receiving biannual (10.7% to 4.8%) SBT ($P < 0.001$). Adults tested in the annual SBT group also showed a decrease in prevalence between Year 1 and Year 5 (12.2% to 11.0%), but this difference was not significant.

These findings are an important consideration for schistosomiasis control programmes that are considering elimination and support the idea that scaling up the frequency of treatment rounds, particularly in areas of low prevalence, will not eliminate schistosomiasis. Interestingly, the finding that prevalence decreased among adults in SBT arms suggests that transmission in the community can be reduced, even where only school children are being treated, which could have logistical and cost-saving implications for the national control programmes.

Annual treatment reduced the prevalence of S. haematobium infection ($p < 0.05$) from 23.1% at baseline to 0.47% after 2 years. Overall cure rate was 97.8%. Intensity of infection declined ($p < 0.05$) from 15.9 eggs/10 ml urine at baseline to 2 eggs/10 ml urine. After two years, overall rate of reinfection was 0.96%. At baseline, total IgG4 was higher in S. haematobium-infected children ($p = 0.042$) ,while all other immunoglobulins were within normal ranges. There was an increase in total IgG2 ($p = 0.044$) levels and a decrease in total IgG4 ($p = 0.031$) levels 2 years post-treatment; and no significant changes in other total immunoglobulins. Schistosoma-infected children at baseline showed an increase in anti-Sh13 IgG1 ($p = 0.005$) and a decrease in Sh13 IgG4 levels ($p = 0.012$) following treatment.

Annual praziquantel treatment delivered to school children over 2 years significantly reduce prevalence, intensity of infection and reinfection of S. haematobium infection. Treatment was also observed to cause a reduction in schistosome-specific blocking IgG4 and an increase in Schistosoma-specific protecting IgG1.

By study end, we observed the lowest S. haematobium prevalence in the biannual MDA, compared to the standard treatment arm (0.6% vs 7.5%; odds ratio [OR] = 0.07, 95% confidence interval [CI] = .02 to .24). The prevalence in arms 2 and 4 was about 3.5%, which was not statistically significantly different from the standard strategy (both ORs 0.4, 95% CI = .1 to ~1.8). New cases of infection were still observed in all arms at study end.

Biannual MDA was the only regimen that outperformed the standard treatment. All strategies resulted in decreased prevalence of infection; however, none of them was able to interrupt transmission of S. haematobium within a 3-year period.

We observed heavy intensity of infection (≥ 50 eggs/10 ml urine) in 87.9% of infected samples and co-occurrence of the eggs of S. haematobium and S. mansoni in urine for two participants. The overall prevalence we recorded is slightly above the national average (9.5%) reported in 2015. Our findings indicate that despite the ongoing administration of praziquantel in Nigeria, urogenital schistosomiasis is still prevalent with heavy intensity of infection. Large-scale epidemiological monitoring is required to monitor the efficacy of schistosomiasis control in Nigeria.

Initial baseline testing in 2008 established that 105 of 247 (42.5%) children were egg-positive. In 2009, with drug treatment alone, the pre-WRA annual cumulative incidence of infection was 29 of 216 (13.4%). In 2010, this incidence rate fell significantly (p<0.001, chi-squared) to 9 of 245 (3.7%) children after installation of the WRA. Logistic regression analysis was used to determine correlates of infection among the variables age, sex, distance between home and river, minutes observed at the river, low height-for-age, low weight-for-age, low Body Mass Index (BMI)-for-age, and previous infection status.

The installation and use of a WRA is a feasible and highly effective means to reduce the incidence of schistosomiasis in school-aged children in a rural Ghanaian community. In conjunction with drug treatment and education, such an intervention can represent a significant step towards the control of schistosomiasis. The WRA should be tested in other water-rich endemic areas to determine whether infection prevalence can be substantially reduced.

Prevalence was reduced from 6.1% (95% confidence interval (CI): 4.5%-7.6%) to 1.7% (95% CI: 1.2%-2.2%) in 9- to 12-year old students, from 3.9% (95% CI: 2.8%-5.0%) to 1.5% (95% CI: 1.0%-2.0%) in adults, and from 8.8% (95% CI: 6.5%-11.2%) to 2.6% (95% CI: 1.7%-3.5%) in 1st year students from 2011/12 to 2017. In 2017, heavy infection intensities occurred in 0.4% of 9- to 12-year old students, 0.1% of adults, and 0.8% of 1st year students. Considering 1st year students in 2017, 13/45 schools in Pemba and 4/45 schools in Unguja had heavy infection intensities >1%. There was no significant difference in prevalence between the intervention arms in any study group and year.	Urogenital schistosomiasis was eliminated as public health problem from most sites in Pemba and Unguja. Prevalence was significantly reduced, but transmission was not interrupted. Continued interventions that are adaptive and tailored to the micro-epidemiology of S. haematobium in Zanzibar are needed to sustain and advance the gains made by ZEST
Uptake of MDA was 39.5% in 2011 and 43.6% in 2012. Prevalence of S. haematobium significantly dropped by 50.0% from 26% in 2011 to 15% in 2012 ($p = 0.000$). Prevalence of S. haematobium was significantly low in MDA participating (3.1%) than non-participating (28.5%) schoolchildren ($p = 0.000$). MDA campaigns had significant impact on knowledge of the disease ($p = 0.02$) and borderline impact on safe water use ($p = 0.04$) but had no impact on avoidance of contact with unsafe water bodies ($p = 0.06$). Bulinus spp. snails were found shedding schistosome cercariae indicating environmental contamination with viable S. haematobium eggs. In conclusion, though MDA significantly reduced prevalence of S. haematobium, uptake was below 50.0% and below the World Health Assembly resolution 54.19 target of 75.0% for 2010.	Non-participation in MDA was the likely source of S. haematobium eggs in the environment hence the observed 15.0% prevalence of S. haematobium infection; and cercarial shedding Bulinus spp. snails indicating continuity of transmission hence the need for further health promotion campaigns.

The overall prevalence of S. mansoni among the school children in the three primary schools in Manna District was 24.0 %. Higher prevalence was recorded for males 25.6 % (61/238) than for females 22.5 % (59/262). Majority (27.5 %) of infection intensity was light with mean faecal egg count (FEC) of 202 eggs per gram (EPG). The therapeutic efficacy of PZQ at a dose of 40 mg/kg was highly efficient (cure rate of 99.1 % and egg reduction rate of 99.9 %) among the school children in the three primary schools in Manna District.

The school children in the three primary schools of Manna District, Jimma Zone were at moderate risk of the morbidity caused by S. mansoni (prevalence > 10 % and < 50 % according to WHO threshold), and hence a biannual MDA with PZQ is required. PZQ available on the local market was found efficient and can be recommended for individual treatment in absence of MDA. The therapeutic efficacy of PZQ at 40 mg/kg against S. mansoni was high in the study area.

The trial was done from Nov 1, 2011, through to Dec 31, 2017 and recruitment took place from Nov 2, 2011, until May 17, 2017. At baseline we enrolled 8278 participants, of whom 2899 (35%) were randomly allocated to arm 1, 2741 (33%) to arm 2, and 2638 (32%) to arm 3. 120 (4·2%) of 2853 in arm 1, 209 (7·8%) of 2688 in arm 2, and 167 (6·4%) of 2613 in arm 3 had S haematobium infections at baseline. Heavy infections (≥50 eggs per 10 mL of urine) were found in 126 (1·6%) of 8073 children at baseline. At the 5-year endline survey, 46 (1·4%) of 3184 in arm 1, 56 (1·7%) of 3217 (odds ratio [OR] 1·2 [95% CI 0·6-2·7] vs arm 1) in arm 2, and 58 (1·9%) of 3080 (1·3 [0·6-2·9]) in arm 3 had S haematobium infections. Heavy infections were detected in 33 (0·3%) of 9462 children.

Biannual MDA substantially reduced the S haematobium prevalence and infection intensity but was insufficient to interrupt transmission. Although snail control or behaviour change activities did not significantly boost the effect of MDA in our study, they might enhance interruption of transmission when tailored to focal endemicity and applied for a longer period. It is now necessary to focus on reducing prevalence in remaining hotspot areas and to introduce new methods of surveillance and public health response so that the important gains can be maintained and advanced.

Observed cure and egg reduction rates were highly dependent on the time point post-treatment. Egg reduction rates were high (>97%) in weeks 3-9 post-treatment. Cure rates were highest in weeks 6 (92.9%) and 9 (95.0%) post-treatment. The prevalence of infection-associated parameters decreased after treatment, reaching a minimum of 2.4% in weeks 5 (proteinuria) and 7 (leukocyturia) post-treatment, and 16.3% at the end of week 8 (microhaematuria). Macrohaematuria disappeared between weeks 3 and 6 post-treatment.

For monitoring praziquantel efficacy against S. haematobium, we recommend that the cure rate is assessed at week 6 post-treatment. The egg reduction rate can be evaluated earlier, from day 14 post-treatment onwards. Reagent strips are a useful additional tool for evaluating treatment outcomes in areas with high endemicity, preferably at weeks 5 and 6 post-treatment. The delayed decrease of microhaematuria confirms that lesions in the urinary tract persist longer than egg excretion post-treatment.

We observed an extremely high prevalence of schistosomiasis (78%) in PSAC before implementation of the program in 2008. Contamination occurred in very young children, as 64.3% of children under 2 years old were infected. Moreover, prevalence increased with age and reached 96.8% in children 4 to < 6 years old. Our annual interventions in Assoni Village raised awareness among villagers that water bodies were areas of significant infestation, allowed the building of 88 latrines and led to a decrease in prevalence in PSAC as only 11% of these children were infected in 2020.

Our study allowed Assoni to be the first village in Senegal to treat PSAC since 2014, but only on an individual basis. It also shows that schistosomiasis is difficult to eradicate and that multi-sectorial actions are required to keep its prevalence at a low level.

Before treatment, 80 out of the 400 children enrolled in the study tested positive for S. haematobium (20.0% (95% confidence interval (CI) 16.4-24.2%). Of these, 41 had infections of heavy intensity (51.3%) while the rest (48.7%) were of light intensity. Five weeks post-treatment, 10 children who had heavy intensity infection were diagnosed with S. haematobium (prevalence: 2.5% (95% CI 1.5-4.9%). Infection intensities decreased significantly from 45.9 (95% CI: 31.0-68.0) eggs/ 10 ml urine to1.4 (95% CI: 1.1-1.7) eggs/ 10 ml urine during pre-and post-treatment respectively. The ERR was 96.9%. There were no severe adverse events during follow up 24 hours post treatment. Treatment tolerability among the 400 children was high as none of the children spat and/ or vomited as observed in this study.

The study revealed that crushed PZQ is safe and effective in the treatment of urogenital schistosomiasis in this age group. It is therefore recommended that PZQ should be administered to the PSC in Kwale County.

The overall reinfection rate at 6 months after treatment was 9.8% (95% confidence interval: 0.5-17.4%). By school, the reinfection rate was highest in the Al Hidaib school, whose prevalence was highest at baseline. The reinfection rate was significantly higher in high-infection areas than low-infection areas (p = 0.02). Of the prevalence at 6 months in high-infection areas, 41% of cases were due to reinfection. MDA interventions are decided upon and undertaken at the district level. A more targeted treatment strategy should be developed with a particular focus on tracking high-risk groups, even within a school or a community.

Abdominal pain, bloody diarrhoea and sleepiness were the most common adverse events, but these were transient and self-limiting. Praziquantel syrup showed moderate-to-high efficacy against Schistosoma haematobium with egg reduction rates of 69.4% and 71.2% 3 and 6 weeks post-treatment and cure rates of 85.7% (95% confidence interval (CI) 79.7-90.5%) and 94.9% (95% CI 90.5-97.6%), respectively. Considerably lower cure and egg reduction rates were observed against Schistosoma mansoni (e.g. cure rate at 6-week post-treatment follow-up was only 50.6% (95% CI 39.9-61.2%). Concluding, praziquantel syrup is well tolerated in preschool-aged children with moderate-to-high efficacy against S. haematobium, but considerably lower efficacy against S. mansoni in Niger. A larger study is warranted to investigate the observed differences in species-specific susceptibilities and to assess operational issues and community-effectiveness.

Out of 562 (309 boys and 253 girls) schoolchildren recruited from three elementary schools, 420 completed one longitudinal dataset that comprised of data from two time points; baseline, and follow-up 1 year after treatment with a single dose of PZQ 40 mg/kg for S. haematobium infection. A single dose of PZQ significantly reduced the prevalence of S. haematobium infection by 83.3% (from 51.4% to 8.6%) and the geometric mean intensity of infection of positive individuals by 17.0% (from 87.7 to 72.8 eggs/10 ml of urine) 1 year after treatment. While there was no significant difference in the reduction of the prevalence of S. haematobium infection between the gender or age groups, there was a significantly higher reduction of intensity of S. haematobium infection among girls in comparison with boys.

These regimens were also associated with reduced odds of undernutrition and reduced odds of portal vein dilation at follow-up. However, neither hemoglobin levels nor the prevalence of the rare abnormal pattern C liver scores on ultrasound improved. For the combined cohorts, growth stunting worsened in the areas receiving biennial SBT, and maximal oxygen uptake as estimated by fitness testing scores declined under both regimens. After adjusting for imbalance in starting prevalence between study arms, children in villages receiving annual CWT had significantly greater decreases in infection prevalence and intensity than those villages receiving biennial SBT. Although health-related quality-of-life scores improved in both study arms, children in the CWT villages gained significantly more. We conclude that programs using annual CWT are likely to achieve better overall S. mansoni morbidity control than those implementing only biennial SBT.

Baseline and year 5 assessments of first-year students and adults were also performed. Using Poisson and negative binomial regression with generalized estimating equations, we found similar effects of CWT and SBT MDA treatment strategies in children aged 9-12 years: significant reductions of prevalence of infection in all arms and of heavy-intensity (≥ 400 eggs/gram) infections in most arms but no significant differences between arms. Combined arms of villages that received four rounds of treatment had greater reduction than villages in arms that only received two rounds of treatment. Surprisingly, we also found benefits of SBT for first-year primary students and adults, who never received treatment in those arms. Our data support the use of annual SBT for control programs when coupled with attention to infections in younger children and occasional treatment of adults.

Schistosomiasis is a neglected tropical disease, typically associated with chronic morbidity, and its control is a global health priority. Praziquantel (PZQ) is the only available antiparasitic drug and is often given out, as a single oral dose (40 mg/kg), to school-aged children by mass drug administration (MDA) schemes operating within preventive chemotherapy campaigns as endorsed by the World Health Organization (WHO). This current strategy has several limitations. (i) It excludes preschool children who can be patently infected. (ii) It delivers PZQ at a dose directly extrapolated from adult pharmacological studies. To address these problems, we conducted the first pharmacokinetic and pharmacodynamic study of young children within an area of Uganda where Schistosoma mansoni is hyperendemic. Our results demonstrate that a higher dose (>60 mg/kg) is required, especially in smaller children, and draw attention to the need for further optimization of PZQ treatment based on schistosome antigenic assays, which are more sensitive to pharmacodynamic markers.

The prevalence of multiple potential morbidity markers did not differ significantly between the egg-positive participants at baseline and those at 5 years by Mann Whitney nonparametric analysis and Fisher's exact test for continuous and categorical data, respectively. There was a small but significantly higher score in school-related quality of life assessment by year five compared to baseline by Mann Whitney analysis ($P = 0.048$) in 13-18 year olds where malaria-negative. However, anemia was not positively impacted by four annual rounds of MDA, but registered a significant negative outcome.

We did not detect differences in morbidity markers measured in a population of those infected or re-infected after multiple MDA. This could have been due to their relative insensitivity or a failure of MDA to prevent morbidity among those who remain infected. High malaria transmission in this area and/or a lack of suitable methods to measure the more subtle functional morbidities caused by schistosomiasis could be a factor. Further research is needed to identify and develop well-defined, easily quantifiable S. mansoni morbidity markers for this age group.

Pre-intervention, fourteen (14) respondent had urinary Schistosomiasis, giving a prevalence of 6.4% with males (64.3%) having the higher prevalence both pre- and post-intervention. Six weeks after intervention, prevalence had reduced to 0.9%, giving a cure rate of about 92%. Of the 22 respondents (10.1%) who had ever noticed 'blood in their urine', only 6 (27.3%) had Schistosoma ova present in their urine samples at the time of the study.

It was concluded that drug treatment with Praziquantel, especially when combined with Health Education was effective in reducing the prevalence of Urinary Schistosomiasis among school-aged children in Langai Community of Plateau State.

Overall, 810 people had complete baseline and follow-up data and were given anthelminthic treatment. The baseline prevalence of hookworm, Schistosoma haematobium, Trichuris trichiura, Schistosoma mansoni and Ascaris lumbricoides was 31.1%, 7.0%, 2.0%, 1.0% and 0.3%, respectively. Four of the five intervention communities were classified open-defecation free. For hookworm infection, we observed higher negative changes in terms of proportion of decrease (-0.10; 95% confidence interval (CI): -0.16, -0.04) and higher egg reduction rate (64.9 vs 15.2%) when comparing intervention with control communities. For intestinal protozoa, prevalence reduction was higher in intervention compared to control communities (8.2 vs 2.6%) and WASH indicators and intervention outcomes associated with lower odds for infection at follow-up. The intervention significantly impacted on reported latrine use (before: 15.5%, after: 94.6%), open defecation in the community surroundings (before: 75.0%, after: 16.7%) and awareness for environmental contamination through open defecation (before: 20.4%, after: 52.2%).

An integrated package of interventions consisting of preventive chemotherapy, health education and CLTS reduces the prevalence of helminth and intestinal protozoa infection. Additional studies in other social-ecological settings are warranted to confirm our findings.

Twenty-five schools were randomly assigned to each arm. S. mansoni prevalence and infection intensity in 9-12 year old students significantly decreased within each arm from baseline to year 5 but there were no differences between arms. There were no differences in infection levels in first year students either within or between arms.

Strategies employing 2 or 4 rounds of MDA had a similar impact in schools with moderate initial prevalence, suggesting that schistosomiasis control can be sustained by school-based MDA, even if provided only every other year.

Overall, 160 preschool-aged children had at least one stool and one urine sample examined with duplicate Kato-Katz thick smears and a point-of-care circulating cathodic antigen (POC-CCA) cassette for S. mansoni, and urine filtration for S. haematobium diagnosis before and 3 weeks after praziquantel administration. According to the Kato-Katz and urine filtration results, we found high efficacy against S. mansoni (cure rate (CR), 88.6%; egg reduction rate (ERR), 96.7%) and S. haematobium (CR, 88.9%; ERR, 98.0%). POC-CCA revealed considerably lower efficacy against S. mansoni (CR, 53.8%). Treatment was generally well tolerated, but moderately severe adverse events (i.e., body and face inflammation), were observed in four Schistosoma egg-negative children.

Crushed praziquantel administered to preschool-aged children at a dose of 40 mg/kg is efficacious against S. mansoni and S. haematobium in a co-endemic setting of Côte d'Ivoire. Further research is required with highly sensitive diagnostic tools and safety must be investigated in more depth.

162 PSAC and 141 SAC participated in the ultrasound examination at baseline, of which 128 PSAC and 122 SAC were present at follow-up. At baseline 43% (70/162) of PSAC had UT morbidity, mostly at bladder level and 7% had hydronephrosis. 67% (94/141) of SAC revealed mainly moderate UT pathology, 4% presented pseudopolyps on the bladder wall, and 6% had pyelectasis. At follow up, 45% of PSAC and 58% of SAC were S. haematobium positive, mostly harboring light infection intensities (41% and 51%, respectively). Microhematuria was present in 33% of PSAC and 42% of SAC and leukocyturia in 53% and 40% of PSAC and SAC, respectively. 50% (64/128) of PSAC and 58% (71/122) of SAC presented urinary tract morbidity, which was mainly mild. A significant correlation ($p<0.05$) was observed between praziquantel treatment and reversal of S. haematobium induced morbidity. Progression of UT pathology decreased with increasing praziquantel dosages. A worsening of morbidity was observed among children in the placebo group.

Bladder morbidity is widespread among PSAC. Praziquantel treatment is significantly associated with the reversal of S. haematobium induced morbidity, which underscores the importance of preventive chemotherapy programs. These programs should be expanded to PSAC to prevent or decrease the prevalence of morbidity in young children. This trial is registered as an International Standard Randomized Controlled Trial, number ISRCTN15280205.

The remaining strategies constituted different combinations of CWT, SBT, and holiday years. Baseline results on S. mansoni infection were obtained from 14,620 schoolchildren from 148 villages, and mean prevalence and mean intensity among infected were 48.6-60.6% and 130.5-229.8 eggs per gram, respectively. Over the years, mean prevalence and mean intensities declined in all arms, but when comparing year 5 mean prevalence and mean intensity, there were no statistically significant differences between treatment arms. Thus, measured in a random selection of schoolchildren aged 9-12 years, four times CWT was not superior to four times SBT, while 2 years of treatment holiday combined with 2 years of SBT had the same impact as 4 years of SBT.

Prevalence comparisons between the two approaches did not show any significant differences following treatment. However, infection intensity levels in the 9-12year old school-attending pupils were significantly higher in the community-wide treatment arm than in the school-based treatment arm. Nevertheless, significant reductions in S. mansoni infection prevalence and intensity levels were achieved among school-age children regardless of the treatment approach used.

At baseline, the overall prevalence of S. haematobium infection was 57.7%, and the proportion of heavy infection was 45.3%, but one month after the first treatment high cure rates (92.9%) were obtained. The overall infection prevalence and proportion of heavy infection intensities were drastically reduced to 4.2% and 2.3%, respectively. The level of the first reinfection in February-March 2012 was 9.5%. At follow-up time points, prevalence levels varied slightly between reinfection and treatment from 9.5% in June 2012 to 0.3% in March 2013, 11.2 in June 2013, and 10.1% April 2014. At the end of the study, overall prevalence was significantly reduced from 57.7% to 10.1%. The overall rate of infested Bulinid snails was reduced after repeated treatment from 0.8% in 2012 to 0.5% in 2013.

Repeated annual treatments are suggested to have a considerable impact on the transmission dynamics of S. haematobium in Niakhar, due to the nature of the epidemiological system with seasonal transmission. Thus, to maintain this benefit and continue to reduce the morbidity of urogenital schistosomiasis, other approaches should be integrated into the strategy plans of the National program to achieve the goal of urogenital schistosomiasis elimination in seasonal foci in Senegal.

The prevalence of schistosomiasis was 14.5% compared to 51% in the prepotable water era (P = 0.001). The intensity of the infection was also significantly reduced between the two eras with 1.3% of the children having a severe intensity compared to 4.5% in the prepotable water era.

The prevalence and intensity of S. haematobium have significantly reduced in this community though not yet eliminated. More boreholes need to be provided to make the water more accessible. This could be combined with other measures to eradicate S. haematobium from this community.

Of the 535 children recruited from the five communities, 169 (31.6%) participated consecutively at all survey points. The highest mean number of samples submitted was 2.9 among communities and survey points. S. haematobium prevalence significantly reduced from 13.3% at baseline to 2.8% at 12 months for all participants and from 24.9% at baseline to 1.8% at 12 months ($P < 0.001$) for participants coming at all- time points. Among the communities, the highest baseline prevalence was found in Chihuri for both the participants coming consecutively (38.5%, 10/26) and all participants (20.4%, 21/103). Reinfections were significantly high at 9 months follow up survey ($P = 0.021$) and in Mupfure ($P = 0.003$). New infections significantly decreased over time ($P < 0.001$). Logistic regression analysis showed that the risk of acquiring schistosomiasis was high in some communities ($P < 0.05$).

S. haematobium infections and reinfections are seasonal and depend on micro-geographical settings. The risk of being infected with schistosomes in pre-school aged children increases with increasing age. Sustained treatment of infected individuals in a community reduces prevalence overtime. Participation compliance at consecutive visits and sample submission adherence are important for effective operational control interventions.

The observed cure rates in cohorts 1 and 2 were 69.7% and 98.2%, respectively. Differences in infection between the cohorts in terms of cure rate and level of infection two months post-treatment were statistically significant and indicate that in areas with a seasonal transmission pattern, the effect of PZQ can be enhanced if treatment takes place during the low transmission season. We conclude that appropriately timed PZQ administration will increase the impact of schistosomiasis control programmes.

S haematobium reinfection intensity was significantly lower in boys in the intervention group than in boys in the control group at 6 months (P < 0.001) and 9 months (P < 0.001) of supplementation. Significantly lower S haematobium reinfection intensity was found in girls in the intervention group than in girls in the control group only at 6 months of supplementation (P = 0.018). Boys in the intervention group were 42% (Adjusted Risk Ratio = 0.58, 95% confidence interval 0.39, 0.86) less likely to be reinfected with S haematobium than in the control group at 6 months follow up.

Once weekly iron supplementation can decrease S haematobium reinfection after 6 months and should be incorporated into school based schistosomiasis control programs in highly endemic areas.

After adjustment, location of residence, age less than 12 years, pretreatment hematuria, and incomplete treatment were the significant independent predictors of infection, whereas sex and frequency of water contact were not. We conclude that local physical features and age-related factors play a predominant role in S. haematobium transmission in this setting. In large population-based control programs, treatment allocation strategies may need to be tailored to local conditions on a village-by-village basis.

This cross-sectional study was conducted among 111 school children aged 7-15 years in Chongwe and Siavonga Districts in Zambia. Species-specific cell-free repeat DNA fragment were amplified from 111 filtered urine samples. Our approach detected eight times more positive cases (total 77) than by KK (9) for S. mansoni and six times more (total 72) than by hematuria (11) for S. haematobium and even more against urine filtration (77 compared to only 6). The same pattern was observed when stratified for age group and sex specific analysis with 100% sensitivity and specificity devoid of any cross amplification. In addition, 69 individuals (62%) were co-infected by both parasites.

We have demonstrated a significantly higher prevalence of both species than indicated by the traditional tests and the persistent maintenance of reservoir of infection after MDA. Our approach is an effective means of detecting low intensity infection, which will enhance the effectiveness of surveillance and assess the impact of MDA control programs against schistosomiasis.

There was no statistically significant difference for the prevalence of infection between males (14.7%) and females (10.2%), although the mean egg count for the females (9.87) was significantly more (P < 0.05) than the males (6.06). At 6 and 12 months post-treatment there was 74.4% and 86.4% reduction in the mean egg count, respectively. Interestingly, an increased prevalence of infection from 2.1% at 6 months to 7.7% at 12 months post-treatment was observed, nonetheless the mean egg count was reduced to 0.27 at 12th month from 1.98 at 6 months post-treatment.

Resurgence in the prevalence rate between 6 and 12 months post-treatment with praziquantel is herein reported and the need for a follow-up treatment in endemic areas for adequate impact on schistosomiasis control is discussed.

There was a significant association between cure rate and intensity of infection prior to treatment with highest cure rates observed in light infections ($P < 0.01$). Praziquantel, at a single dose of 40 mg/kg, was again administered 35 days after the first treatment. The overall cure and egg reduction rates increased considerably. The association between cure rate and intensity of infection prior to the second treatment was significant but less pronounced. Twenty-two children remained S. mansoni positive after the two chemotherapy campaigns, and interestingly, many of these were only identified after repeated stool examinations.

We argue that pre-patent infections may account for some of these 'treatment failures'. However, further studies in other endemic settings are needed, with parasitological diagnoses having a high sensitivity.

The risk of reinfection by Schistosoma haematobium was higher in children between the ages of 7 and 10 years than in children between the ages of 11 and 15 years ($p < 0.001$), The incidence of intense Schistosoma haematobium egg excretion rose from 0 p. 100 in July 1989 to 6.0 p. 100 in February 1990. The incidence of intense Schistosoma mansoni excretion in February 1990 was 4.5 p. 100. The reinfection rate at 7 months was over 50 p. 100 for both parasite species despite repeated treatment. This finding demonstrates that additional measures such as proper sanitation and vector control are needed to control human schistosomiasis in irrigated rice paddies.

Results: The results of thisstudy show the endemic prevalences of urinary schistosomiasisin all ecological areas ofthe Senegal River Basin: 57.4% in the Delta, 32.5% in the Valley and 25.1% in the upper basin. The prevalence of the intestinalschistosomiasisform was 21.8 % in the Delta, and this form has also entered the valley. Conclusion: The results ofthisstudy confirm that schistosomiasis is still a public health problem in the Senegal River Basin despite several series of mass praziquantel 600 mg administration. This situation requires detailed reflection concerning dosing frequencies of this drug in the Senegal River basin and the need to take social behaviours and sociological realities into account in order to eradicate schistosomiasis.

At baseline S. haematobium infection prevalence and intensity were 31.7% (95% CI = 31.1-32.2) and 28.75 eggs/10ml urine (SEM = 0.81) respectively, while S. mansoni prevalence and intensity were 4.6% (95% CI = 4.4-4.8) and 0.28 eggs/25mg (SEM = 0.02). Prior to the 6th round of MDA, S. haematobium infection prevalence had reduced to 1.56% ($p<0.001$) and infection intensity to 0.07 (SEM 0.02). Six weeks later after the 6th MDA, both were 0. Similarly the prevalence of S. haematobium morbidity as indicated by haematuria also fell significantly from 32.3% (95% CI = 29.9-34.6) to 0% ($p< 0.0001$) prior to the final MDA. For S.mansoni, both prevalence and intensity had decreased to 0 prior to the 6th MDA. After 6 rounds of annual MDA, prevalence and intensity of both schistosome species decreased significantly to 0% ($p< 0.0001$).

Zimbabwe's helminth control program significantly reduced schistosome infection intensity and prevalence and urogenital schistosomiasis morbidity prevalence in a cohort of school-aged children, moving the schistosome prevalence in the children from moderate to low by WHO classification. These findings will inform the design of the country's next stage interventions for helminth control and eventual elimination.

The prevalence rate of Sh showed a significate decreasing from 2011, 2013 to 2014 with respectively 10.2% [95% CI=10.04-10,18], 5.32% [95% CI=5.30-5.33], and 5.25% [95% CI=524.-5.31], followed by an increase to 10.6% [95% CI = 10.47-10.63] in 2015 and a significative decrease in 2016 to 5.4% [95% CI=3.5-7,3]. Children aged from six to 10 years and mostly boys were more infected with Sh, then could serve of parasite reservoir. MDA with PZQ remains an effective strategy for schistosomiasis control against Sh in Kalifabougou. Additional studies on MDA/PZQ average treatment covering human-water contact behaviors and population migration are necessary to understand the persistence of the 5% annual prevalence rate of egg shedding in the cohort of volunteers periodically treated with PQZ. Testing eggs shed viability will be also an added value.

These were reduced to 33.2 eggs/10 ml of urine and 43.2 epg in 2010 respectively, a significant reduction of 81.6% and 51% (p<0.001). The proportion of heavy S. haematobium infections was reduced from 48.8% in 2004 to 13.8% in 2010, and the proportion of moderate and heavy S. mansoni infection was reduced from 15.6% in 2004 to 9.4% in 2010, both significantly (p<0.01). Mathematical modelling suggests that the observed results were in line with the expected changes.	Significant reduction in intensity of infection on both infections and modest but significant reduction in S. haematobium prevalence were achieved in highly-endemic Segou region after repeated chemotherapy. However, persistent prevalence of both infections and relatively high level of intensity of S. mansoni infection suggest that more intensified control measures be implemented in order to achieve the goal of schistosomiasis elimination. In addition, closer monitoring and evaluation activities are needed in the programme to monitor the drug tolerance and to adjust treatment focus
Data from the longitudinal cohort show that a single round of PZQ treatment significantly reduced prevalence of S. haematobium infection by 87% (from 59.6% to 7.7%) and intensity of infection by 92.8% (from 94.2 to 6.8 eggs/10 ml of urine) 2 years post-treatment. The impact on infection was also confirmed by a cross-sectional survey 2 years post-treatment. Importantly, the proportion of school-age children with heavy S. haematobium infection decreased from around 25% before treatment to around 2-3% 2 years post-treatment. Cross-sectional comparison of S. haematobium infection in 7-year-old children in their first year at school, who received treatment through community-based drug delivery, also showed significant reduction in both prevalence (65.9%) and intensity of S. haematobium infection (78.4%) 2 years after single treatment. A significant reduction in S. mansoni infection was also achieved.	Significant and sustained reduction in S. haematobium infection was achieved by biennial treatment in school-age children in Burkina Faso. This may provide a cost-effective treatment strategy for similar national schistosomiasis control programmes in sub-Saharan Africa.

Using a negative binomial regression fitted to egg count data, we found that every percentage point increase in piped water coverage was associated with 4.4% decline in intensity of re-infection (incidence rate ratio = 0.96, 95% CI: 0.93-0.98, p=0.004) among the treated children. We therefore provide further compelling evidence in support of the scaleup of piped water as an effective control strategy against Schistosoma haematobium transmission.

The present random allocation study examined the relative efficacy of a 20 mg/kg dose versus a 40 mg/kg dose of praziquantel in control of hematuria and bladder and renal abnormalities associated with S. haematobium infection in an endemic area of Coast Province, Kenya. After a nine-month observation period, the results indicated an advantage to the standard 40 mg/kg praziquantel dose in terms of reduction of infection prevalence and hematuria after therapy (P < 0.01 and P < 0.005, respectively). However, the two treatment groups were equally effective in reducing structural urinary tract morbidity detected on ultrasound examination. We conclude that in certain settings, a 20 mg/kg dose of praziquantel may be sufficient in providing control of morbidity due to urinary schistosomiasis in population-based treatment programs.

Diagnosis of S. mansoni infection and cure assessment were based on examination of 2 Kato slides prepared from a single stool sample collected before and 4 weeks after the first and second treatments. The cure rate was 78·8% after the first treatment and increased significantly to 90·8% after the second treatment. Egg reduction rates were 71·2% and 77·2% after 1 and 2 treatments respectively. Pre-treatment intensity of infection has a great influence on cure and egg reduction rates. Our results confirmed that low praziquantel cure rate, in Egypt, might be attributed, even partially, to survival and maturation of the immature S. mansoni stages that escaped pzq that is effective against mature worms only

This study will provide data on the efficacy of repeated PZQ treatment on the clearance of S. mansoni as measured by several diagnostic techniques. These findings will inform future mass drug administration policy and shed light on position of novel diagnostic tools to evaluate schistosomiasis control strategies.

Praziquantel treatment was highly efficacious at three weeks after treatment when judged by egg reduction rate (95.3%) and cure rate of heavy infections (94.1%). The apparent overall cure rate three weeks after treatment (57.9%) was much lower but improved to 80.7% at 41 weeks after treatment. Re-infection with S. haematobium was low and appeared to be limited to the hot and rainy summer. Analysis of only one urine specimen per child considerably underestimated prevalence when compared to the analysis of two specimens, but both approaches provided similar estimates of the proportion of heavy infections and of average infection intensity in the population.

According to WHO guidelines the high prevalence and intensity of S. haematobium infection necessitate regular treatment of schoolchildren in the area. The seasonal transmission pattern together with the slow pace of re-infection suggest that one treatment per year, applied after the end of summer, is sufficient to keep S. haematobium infection in the area at low levels.

At baseline, the overall prevalence and the heavy intensity of infection were 73.2% and 356.1 eggs/10 ml of urine. Significant differences in the prevalence and intensity of S. haematobium infection were noted between villages. A single dose of PZQ significantly reduced the prevalence of S. haematobium infection from 73.2% to 4.6% and the geometric mean intensity of infection from 356.1 to 43.3 eggs/10 ml of urine. The cure rates ranged from 89.4% to 100%. The egg reduction rates also ranged from 77.6% to 100%. Two to three months after the period of transmission, the overall rate of reinfection was 12.6% and was significantly higher in male children than in female children. The overall prevalence at this period was 13.8%, which was significantly lower than the prevalence at baseline (73.2%).

The Niakhar study area remains a hot spot of urinary schistosomiasis in Senegal with differences in transmission between villages. This study suggests that when transmission is strictly seasonal, Praziquantel shows the expected efficacy in reducing the prevalence and intensity of infection, but also a significant effect on the occurrence of reinfection.

Two rounds of treatment significantly reduced the prevalence of S. mansoni infection in schoolchildren across three regions in the country from 33.4-49.3% to 9.7-29.6%, and intensity of infection from 105.7-386.8 eggs per gram of faeces (epg) to 11.6-84.1 epg. The prevalence of hookworm infection was reduced from 41.2-57.9% to 5.5-16.1%, and intensity of infection from 186.9-416.8 epg to 3.7-36.9 epg. The proportion of children with heavy S. mansoni infection was significantly reduced from 15% (95% CI 13.4-16.8%) to 2.3% (95% CI 1.6-3.0%). In adults, significant reduction in the prevalence and intensity of S. mansoni and hookworm infections was also observed.

More importantly, the prevalence and intensity of both S. mansoni and hookworm infections in the cohorts of newly-recruited 6-year-olds who had never previously received treatment decreased significantly over 2 years: 34.9% (95% CI 31.9-37.8%) to 22.6% (95% CI 19.9-25.2%) and 171.1 epg (95% CI 141.5-200.7) to 72.0 epg (95% CI 50.9-93.1) for S. mansoni; and 48.4% (95% CI 45.4-51.5) to 15.9% (95% CI 13.6-18.2) and 232.7 epg (95% CI 188.4-276.9) to 51.4 epg (95% CI 33.4-69.5) for hookworms, suggesting a general decline in environmental transmission levels.

Overall, 7,[illegible] children aged 9-12 years were examined at baseline and 7,223 at the final survey. The baseline prevalence of S. mansoni was 17.4%, 20.2%, and 25.2% in arms 1, 2, and 3, respectively. In the final year, we observed the lowest prevalence of 10.4% in arm 1, compared to 18.2% in arm 2 and 17.5% in arm 3. The comparison between arms 1 and 2 estimated an odds ratio (OR) of 0.52 but the difference was not statistically significant (95% confidence interval (CI) = 0.23-1.16). Likewise the difference between arms 1 and 3 lacked statistical significance (OR = 0.55, 95% CI = 0.23-1.29). There was no noteworthy difference observed between arms 2 and 3 (OR = 1.06, 95% CI = 0.64-1.75). The lowest S. mansoni fecal egg counts in the final year survey were observed in arm 1 (7.9 eggs per gram of stool (EPG)). However, compared with 11.5 EPG in arm 2 and 15.4 EPG in arm 3, the difference lacked statistical significance. There were 4,812 first-grade children examined at baseline and 4,513 in the final survey. The overall prevalence of S. mansoni in these children slightly decreased in arms 1 (from 4.5% to 3.6%) and 2 (from 4.7% to 4.3%), but increased in arm 3 (from 6.8% to 7.9%). However, there was no significant difference in prevalence and infection intensity observed between study arms.

The three treatment schedules investigated led to a reduction in the prevalence and intensity of S. mansoni infection among children aged 9-12 years. Comparing intervention arms at the end of the study, no statistically significant differences were observed between annual treatement and the other two treatment schedules, neither in reduction of prevalence nor intensity of infection. It is important to combine our results with those of three sister trials conducted simultaneously in other African countries, before final recommendations can be drawn.

Liver function testing was performed for all children who received combined therapy. The study included 48 children with LF alone, 60 children with schistosomiasis (Schistosoma mansoni), 41 children with STH, 49 children with schistosomiasis + LF and 37 children with all three types of infection. Children were closely monitored by a paediatrician for any adverse reactions for 7 days. No serious adverse events were experienced. However, 4 of 18 children in the test group and 2 of 3 children in the control group who did not report any ill conditions before treatment developed adverse drug reactions. The combined and conventional therapies were found to be equally safe. The efficacies of both therapies were comparable and satisfactory.

Distended abdomens, defined as an abdominal circumference ratio (ACR) >1.05, were observed in 2.5% of the sampled children, several of whom presented with particularly severe distensions necessitating hospital referral. ACR scores were highly overdispersed between districts and schools. Multivariate regression analysis revealed that S. mansoni infection accounted for only a small fraction of ACR variation, suggesting that either single point prevalence and intensity measures failed to reflect this more chronically evolved morbidity and/or that other interacting factors were involved, e.g. malnutrition and malaria. At 1-year follow-up, ACR scores showed an overall trend of regression towards the mean, potentially indicative of amelioration following chemotherapy, but geographic overdispersion still remained

Schistosome soluble egg antigen (SEA) and soluble worm antigen preparation (SWAP) stimulated higher IL-5 production by egg-negative children in the post-MDA group compared to the baseline group. Similarly, anti-SEA IgE levels were higher in egg-negative children in the post-MDA group compared to the baseline group. Anti-SEA and anti-SWAP IgG4 levels were lower in egg-negative children in the post-MDA group compared to baseline. This resulted in higher anti-SEA IgE/IgG4 ratios for children in the post-MDA group compared to baseline. These post-MDA immunological changes are compatible with the current paradigm that treatment shifts immune responses to higher antischistosome IgE:IgG4 ratios in parallel with a potential increase in resistance to reinfection

Sixty-one children were present on all examination time points and had complete datasets. No difference in efficacy was observed between the three treatment groups on either follow-up. On the 21-22 day posttreatment follow-up, based on available case analysis, cure rates of 33% (95% confidence interval (CI) 11-55%), 29% (95% CI 8-50%), and 26% (95% CI 5-48%) were observed for praziquantel, mefloquine-artesunate-praziquantel, and mefloquine-praziquantel, respectively. The corresponding egg reduction rates were 94% and above. On the second follow-up, observed cure rates ranged from 19% (praziquantel) to 33% (mefloquine-artesunate-praziquantel), and egg reduction rates were above 90%. Praziquantel monotherapy was the best tolerated treatment. In the mefloquine-artesunate-praziquantel group, adverse events were reported by 91% of the participants, and in the mefloquine-praziquantel group, 95% experienced adverse events. With the exception of abdominal pain at moderate severity, adverse events were mild.

The addition of mefloquine or mefloquine-artesunate does not increase the efficacy of praziquantel against chronic S. haematobium infection. Additional studies are necessary to elucidate the effect of the combinations against acute schistosomiasis.

One year after a single-dose praziquantel treatment (administered using the WHO PZQ dose pole) co-administered with albendazole (400 mg single dose) for de-worming, the prevalence of S. haematobium infection was 38%, while the prevalence of anaemia fell to 50.4%. The mean haemoglobinemia showed a statistically significant increase of 0.39 g/dl to reach 11.4 g/dl. Anaemia was no longer associated with S. haematobium or to P. falciparum infections, or to haematuria or ultrasound abnormalities of the urinary tract.

The high prevalence of anaemia in Nigerian children is clearly a result of many factors and not of schistosomiasis alone. Nevertheless, treatment of schistosomiasis and de-worming were followed by a partial, but significant, reduction of anaemia in schoolchildren, not explainable by any other obvious intervention.

The prevalence of hard spleens, and the magnitude of clinically assessed splenomegaly along the mid-axillary and mid-clavicular lines decreased monotonically over time, independently of age, whereas clinically measured hepatomegaly along the mid-sternal line and the prevalence of firm livers decreased in an age-specific manner, being more pronounced amongst children aged 14 years or older at enrolment. Ultrasound data were less informative, and did not concur with clinical observations. These results demonstrate that praziquantel treatment reduces hepatosplenomegaly in the absence of exposure to S. mansoni, even with continuing exposure to malaria. The lack of complete resolution of hepatosplenomegaly in most children suggests, among other things, a residual organomegaly attributable to malaria

At the end of the study, prevalence of infection among the PZQ/ART was approximately half that of the PZQ/ART-placebo group, i.e. 6.7% versus 11.6%, and incidence of new infections for the PZQ/ART was 2.7% versus 6.5% for the PZQ/ART-placebo. In conclusion, PZQ/ART combined therapy might be considered as an adjunct measure against human schistosomiasis, by specifically reducing transmission and therefore contribute to disease elimination.

Self reported uptake of praziquantel was 44.7% (275/615), 95% confidence interval (CI) 40.8-48.7%. Of the 275 community members who said they had swallowed praziquantel, 142 (51.6%) reported that they had developed side effects. Uptake of MDA was more likely if the respondent was knowledgeable about schistosomiasis transmission and prevention (adjusted odds ratio [AOR] 1.85, 95% CI 1.22-2.81) and reported to have received health education from the health personnel (AOR 5.95, 95% CI 3.67-9.65). Service delivery challenges such as drug shortages and community health worker attrition also influenced MDA in Koome Islands.

Uptake of MDA for schistosomiasis control in Koome was sub optimal. Lack of knowledge about schistosomiasis transmission and prevention, inadequate health education and drug shortages are some of the major factors associated with low uptake. These could be addressed through routine health education and systematic drug supply for the successful elimination of schistosomiasis on the islands.

The programme was implemented by schoolteachers and coordinated by the District Health Management Team in collaboration with the District Education Office. Teachers were responsible for carrying out all programme activities. Community participation was through collaboration with Teachers-Parents Associations and Village Health Committees. Coverage at yearly (1995-99) examination varied from 67.7% to 80.3%. Prevalence of haematuria decreased from 51.2% (range 22.2-89.5%) at baseline to 23.4% (range 5.8-56.7%) in 1999, a reduction of 54.3%. Macrohaematuria was 21.2% at baseline and 7.2% in 1999, a reduction of 66.0%. Prevalence of infection in class 5 was reduced by 71.4% and geometric mean intensity of positives reduced from 71 eggs/10 mL (95% confidence interval [CI] 52.5-97.7) to 28 eggs/10 mL (95% CI 25.7-55.0), a reduction of 60.6%.

Post-treatment stool examination was carried out 8 weeks later, and a total of 1942 stool samples were collected. Prevalence decreased to 8.6% for S. mansoni, 0.2% for N. americunus, 0 for A. lumbricoides, and 0.6% for T. trichiura. Efficacy was good for S. mansoni and N. americanus (92.6% and 95.0%, respectively). Results of the first round of treatment of school-age children in Mwea indicate a good reduction in parasite burden.

Teachers were highly committed, and secured community participation and a smooth implementation of the programme. The community accepted the introduction of a cost-recovery system, whereby parents pay for the treatment of children with episodes of visible haematuria during the school year. Communities also participated in the improvement of sanitary installations at the schools.

The reduction achieved in the prevalence and intensity of schistosomiasis after an intervention period of four years was limited. This observation corroborates the fact that molluscicides must always be considered as supplementary to chemotherapy in the control of schistosomiasis. Although both approaches can be used, the spraying approach appears to be simpler and more feasible because two or three times yearly application of Endod suspension would suppress snail population and reduce transmission. Nevertheless, the choice as to what approach to use must be made on the basis of community preference, and for some soap-effect of Endod would be attractive

Overall reported treatment coverage of Praziquantel (PZQ) against SCH was 4286 (75.5%). Males were 27% more likely to swallow the drug (AOR = 1.27; 95% CI: 1.09, 1.47) than females. SAC with age 10-14 years were 45% more likely to swallow the drug compared with their counter parts (5-9 years), (AOR =1.45; 95% CI: 1.25, 1.69). There is statistically significant association between PZQ swallowing status with school enrollment. (AOR = 20.90, 95% CI: 17.41, 25.08). Swallowing status of PZQ against SCH significantly higher for SAC treated in districts applied integrated treatment approach (87.5%) compared with SAC treated in vertical treatment approach (72.5%); P-value < 0.001. SACs were asked for reasons for not taking the drug and the main reported reason for not swallowing PZQ in the present study was none attending of the school.

Over all treatment coverage of PZQ against SCH in the present study was 75.5%. Although it is in accordance with WHO recommendation for Ethiopia, national programmatic improvements are necessary to achieve higher coverage in the future. To increase treatment coverage for PZQ against SCH in Ethiopia, school based training should target all schools. Moreover, mobilization, sensitization and implementation of the community wide treatment need to be improved.

During baseline screening, 1,022 children were assessed for eligibility of whom 153 (15%) had a detectable S. mansoni infection, and hence, were randomized to the standard treatment group (N = 70) and the intense treatment group (N = 83). Based on KK, the CR was 42% (95% confidence interval (CI) 31-52%) in the standard treatment group and 86% (95% CI 75-92%) in the intense treatment group. Observed IRR was 72% (95% CI 55-83%) in the standard treatment group and 95% (95% CI 85-98%) in the intense treatment group. The CR estimated by POC-CCA was 18% (95% CI 11-27%) and 36% (95% CI 26-46%) in the standard and intense treatment group, respectively. Repeated PZQ treatment did not result in a higher number of adverse events.

The observed CR using KK was significantly higher after four repeated treatments compared to a single treatment, without an increase in adverse events. Using POC-CCA, the observed CR was significantly lower than measured by KK, indicating that PZQ may be considerably less efficacious as concluded by KK. Our findings highlight the need for reliable and more accurate diagnostic tools, which are essential for monitoring treatment efficacy, identifying changes in transmission, and accurately quantifying the intensity of infection in distinct populations. In addition, the higher CR in the intense treatment group suggests that more focused and intense PZQ treatment can help to advance schistosomiasis control.

We found no evidence of reduced transmission or schistosome population decline over the course of the program. Although prevalence declined in the 67 children as it did in the overall program, reinfection rates were high, and for the 15 children studied in detail, schistosome egg counts and estimated adult worm burdens did not decline between years 1 and 4, and genetic diversity increased over the course of drug treatment.

School based control programs undoubtedly improve the health of individuals; however, our data show that in an endemic area, such a program has had no obvious effect on reducing transmission or of significantly impacting the schistosome population as sampled by the children we studied in depth. Results like these, in combination with other sources of information, suggest more integrated approaches for interrupting transmission and significantly diminishing schistosome populations will be required to achieve sustainable control.

We demonstrated that there were individual parasites with reduced PZQ susceptibility in the 2010 collections, as evidenced by our in vitro larval behavioural phenotypic assay. There was no evidence, however, that miracidia showing phenotypically reduced susceptibility clustered together genetically. Molecular analysis also demonstrated a significant reduction of adult worm load over time, despite little evidence of reduction in parasite infection intensity, as measured by egg output. Genetic diversity of infections did not reduce over time, despite changes in the genetic composition of the parasite populations.

Genotypic and phenotypic monitoring did not indicate a selective sweep, as may be expected if PZQ treatment was selecting a small number of related "resistant" parasites, but there was evidence of genetic changes at the population level over time. Genetic data were used to estimate adult worm burdens, which unlike parasite infection intensity, showed reductions over time, suggesting the relaxation of negative density-dependent constraints on parasite fecundity with PZQ treatment. We thereby demonstrated that density-dependence in schistosome populations may complicate evaluation and monitoring of control programmes.

The four approaches are 1) absolute percent change in prevalence; 2) percent change in prevalence; 3) change in World Health Organization guideline categories; 4) change (absolute or percent) in both prevalence and intensity. We compare and contrast the outcomes of these analyses. Our intent is to show how the same dataset yields different numbers of persistent hotspots depending on the approach used to define them. We suggest that investigators and NTD program managers use the approach most suited for their study or program, but whichever approach is used, it should be clearly stated so that comparisons can be made within and between studies and programs.

Uptake of praziquantel was higher in the snack schools, 93.9% (95% CI 91.7%-95.7%), compared to that in the non-snack schools, 78.7% (95% CI 75.4%-81.7%) ($p = 0.002$). The occurrence of side effects was lower in the snack schools, 34.4% (95% CI 31.5%-39.8%), compared to that in the non-snack schools, 46.9% (95% CI 42.2%-50.7%) ($p = 0.041$). Prevalence and mean intensity of S. mansoni infection was lower in the snack schools, 1.3% (95% CI 0.6%-2.6%) and 38.3 eggs per gram of stool (epg) (95% CI 21.8-67.2), compared to that in the non-snack schools, 14.1% (95% CI 11.6%-16.9%) ($p = 0.001$) and 78.4 epg (95% CI 60.6-101.5) ($p = 0.001$), respectively.

In the Kou Valley, the prevalence went up from 14% in 1957 to 80% in 1974 for urinary schistosomiasis and from 1.3% to 45% for intestinal schistosomiasis. The same tendencies are likely to appear in the hydraulic installations of Bagré, Ziga, and Kompienga. Dams thus constitute amplifying factors for the proliferation of species and for parasite-host interactions. All the actors (developers, populations and scientists) are faced with the challenge of finding a mean to control the development of schistosomiasis infections which are likely to seriously lessen the benefits expected from these hydraulic installations.

The program we have implemented from 2008 in partnership with the PNLB/WHO involved campaigns to 1) evaluate schistosomiasis prevalence in children of 53 villages around Ninefescha hospital, 2) perform a mass drug administration following the protocol established by the PNLB in school-aged children, 3) monitor annual prevalence, 4) implement health education campaigns, and 5) oversee the building of latrines. This campaign led to a drop in schistosomiasis prevalence but highlighted that sustainable schistosomiasis control by praziquantel treatment, awareness of the use of latrines, and inhabitants' voluntary commitment to the program are crucial to improve Schistosoma elimination.

Moreover, this study revealed that preschool-aged children, for whom praziquantel was not recommended until 2014 in Senegal, constituted a significant reservoir for the parasite.

The standard arm will receive PZQ at week 8 and 52. We expect to enrol 480 participants, with 80% infected with S. mansoni at the outset.Primary outcomes are BCG-specific interferon-γ ELISpot responses 8 weeks after BCG immunisation and for other vaccines, antibody responses to key vaccine antigens at 4 weeks after immunisation. Secondary analyses will determine the effects of intensive anthelminthic treatment on correlates of protective immunity, on waning of vaccine response, on priming versus boosting immunisations and on S. mansoni infection status and intensity. Exploratory immunology assays using archived samples will enable assessment of mechanistic links between helminths and vaccine responses.

Ethics approval has been obtained from relevant ethics committes of Uganda and UK. Results will be shared with Uganda Ministry of Health, relevant district councils, community leaders and study participants. Further dissemination will be done through conference proceedings and publications.

One year later, the score was 2.43 for schistosomiasis and 2.70 for malaria from 779 children (351 boys and 428 girls). As might be expected, knowledge and attitudes scores for schistosomiasis increased (+0.05), but not as much as originally hoped, while the score for malaria decreased (-0.33). According to a Kolmogorov-Smirnov test, neither change was statistically significant. Analysis also revealed that 75% of school children misunderstood the importance of reinfection after treatment with praziquantel. These results are disappointing.

They demonstrate that it is mistaken to assume that knowledge conveyed in child-friendly booklets will necessarily be interpreted, and acted upon, in the way intended. If long-term sustained behavioural change is to be achieved, health education materials need to engage more closely with local understandings and responses to urogenital schistosomiasis. This, in turn, needs to be part of the development of a more holistic, biosocial approach to the control of schistosomiasis.

Pre-treatment S. haematobium infection intensity in 1-5 year olds was 14.6 eggs/10 ml urine and prevalence was 21%. Of the 104 children, 3.8% reported side effects within 24 hours of taking PZQ treatment. These were stomach ache, loss of appetite, lethargy and inflammation of the face and body. PZQ treatment significantly reduced schistosome infection levels in 1-5 year olds with an egg reduction rate (ERR) of 99% and cure rate (CR) of 92%. This was comparable to the efficacy of praziquantel in 6-10 year olds where ERR was 96% and CR was 67%.
INTERPRETATION/SIGNIFICANCE: PZQ treatment is as safe and efficacious in children aged 1-5 years as it is in older children aged 6-10 years in whom PZQ is the drug of choice for control of schistosome infections.

Between Nov [illegible], 2014, and Feb [illegible], [illegible], [illegible] PSAC and 225 SAC were assessed for eligibility; of whom 161 (24%) PSAC and 180 (80%) SAC had a detectable Schistosoma mansoni infection. 161 PSAC were randomly allocated of whom 154 received treatment: 42 were assigned to 20 mg/kg praziquantel, of whom 40 received treatment; 38 were assigned to 40 mg/kg praziquantel, of whom 38 received treatment; 41 were assigned to 60 mg/kg praziquantel, of whom 39 received treatment; and 40 were assigned to placebo, of whom 37 received placebo. 180 SAC were randomly allocated of whom 177 received treatment: 49 were assigned to 20 mg/kg praziquantel, of whom 47 received treatment; 46 were assigned to 40 mg/kg praziquantel, of whom 46 received treatment; 42 were assigned to 60 mg/kg praziquantel, of whom 42 received treatment; and 43 were assigned to placebo, of whom 43 received treatment. Follow-up (available-case) data were available for 143 PSAC and 174 SAC. In PSAC, the 20 mg/kg dose resulted in cure in 23 children (62%; 95% CI 44·8-77·5), 40 mg/kg in 26 children (72%; 54·8-85·8), 60 mg/kg in 25 children (71%; 53·7-85·4), and placebo in 13 children (37%; 21·5-55·1). In SAC, the 20 mg/kg dose resulted in cure in 14 children (30%; 95% CI 17·7-45·8), 40 mg/kg in 31 children

Praziquantel shows a flat dose-response and overall lower efficacy in PSAC compared with in SAC. In the absence of treatment alternatives, a single dose of praziquantel of 40 mg/kg, recommended by the WHO for S mansoni infections in SAC can be endorsed for PSAC in preventive chemotherapy programmes.

In total 47.4% of children were already aware that schistosomiasis was a water-borne disease while only 10.5% knew of its exact aetiology; after booklet intervention these levels increased to 54.6 and 15.7%, respectively. The majority of children still failed, however, to realise that re-infection could take place soon after treatment. While a positive increase was observed for children's total KA questionnaire scores for both malaria and schistosomiasis after booklet intervention, these were not statistically significant. In the context of control, further educational efforts are needed to promote and guide behavioural change, especially in relation to reduction of environmental water contact.

The notion of reinfestation remains little known. Behaviours that favour the illness were ignored by 1/3 of people interrogated in the project area. However, there was an increase in knowledge about the illness in the program zone in comparison with the control area. Despite the increase in knowledge level, changes in behaviour in relation to the illness remained low. Risky behaviour continued in about 2/3 of people interrogated. Only 33% of persons of the project area declared having adopted at least a single good behaviour. Changes of behaviour are slow to take place. Activities of health education must be sustained throughout a long period of time for sustainable profits of control actions to occur.

At preassessment, 0/98 (0.0%) children in the intervention group had heard of praziquantel compared with 2/177 (1.1%) in the control group. Similarly, 0/98 (0.0%) children in the intervention group did not know that praziquantel does not kill compared with 4/177 (2.3%) in the control group. The postassessment showed that 53/78 (67.9%) in the intervention group were aware of praziquantel compared with 2/177 (1.1%) in the control group (p=0.000). Similarly, 53 (69.7%) in the intervention group knew about the safety of praziquantel compared with 0/177 (0.0%) in the control group (p=0.000). Sixty-four children (65.3%) from the intervention group sought praziquantel treatment after the trial.

2 year-Cohort study

Schisto and Ladders version 2 is a useful sensitisation tool with which to encourage compliance to praziquantel treatment in schools.

[illegible]
post-treatment, 10 were infected. Six of these were new
infection cases, while four were cases of re-infection. The intensity of infection had decreased
significantly (p = 0.001) at the time of the follow-up survey compared to the baseline survey. However, no
significant difference was found among

Randomised dose finding trial	received treatment. Almost 90% of PSAC and three quarters of SAC were lightly infected with S. haematobium. Follow-up data were available for 157 PSAC and 166 SAC. In PSAC, CRs of praziquantel were 85.7% (30/35), 78.0% (32/41) and 68.3% (28/41) at 20, 40 and 60 mg/kg and 47.5% (19/40) for placebo. In SAC, CRs were 10.8% for placebo (4/37), 55.6% for 20 mg/kg (25/45), 68.3% for 40 mg/kg (28/41) and 60.5% for 60 mg/kg (26/43). ERRs based on geometric means ranged between 96.5% (60 mg/kg) and 98.3% (20 mg/kg) in PSAC and between 97.6% (20
Systematic Review	Contrary to earlier records, S. haematobium was low and confined to a few putative foci, with declined in infections from 28.2% in 1951 to 2.48% by 2011. Although this decline can be attributed to control, this was already much lower in 1967 than 1951, long before control interventions began suggesting that environmental changes may have made the habitat less suitable for the transmission of S. haematobium. Compared to the
Epidemiological study	Seventy children who had previously participated in an epidemiological study on schistosomiasis in the city of Cubal, Angola, and had also performed urinary ultrasound between August 2013 and February 2014 were cited 6-8 months later to assess the possible reinfection and repeat new urinary ultrasound, analyzing changes at the level of urinary pathology. The presence of hematuria and proteinuria was also analyzed. Of the 70 children analyzed, 29 (41.4%) were girls, with an average age of 10.4 years (standard

	No subject with heavy intensity of infection was detected in Fayoum and Bani Sweif governorates. Of the 39 studied districts 97.4% had prevalence of heavy intensity infection of <1%, indicating elimination of schistosomiasis haematobia as a public health problem in these districts. Of those studied 72.0% were male. Males were 2.9 times as likely to be infected (1.5% [95% CI: 1.4-1.7]) as females (0.5% [95% CI: 0.3-0.7]); $\chi 2 = 51.2$, $p < 0.0001$. Heavy intensity of infection was detected only in males. The prevalence of S. haematobium infection
12 month Compliance study	Of the 535 children recruited from the five communities, 169 (31.6%) participated consecutively at all survey points. The highest mean number of samples submitted was 2.9 among communities and survey points. S. haematobium prevalence significantly reduced from 13.3% at baseline to 2.8% at 12 months for all participants and from 24.9% at baseline to 1.8% at 12 months ($P < 0.001$) for participants coming at all- time points. Among the communities, the highest baseline prevalence was found in Chihuri for both the participants coming consecutively (38.5%, 10/26) and all
A pilot survey	School-aged children (SAC) have a considerable burden of intestinal schistosomiasis in Madagascar yet its burden in pre-school aged children (PSAC) is currently overlooked. To assess the at-risk status of PSAC, we undertook a pilot epidemiological survey in June 2019 examining children (n = 89), aged 2-4-years of balanced gender, in six remote villages in Marolambo District, Madagascar. Diagnosis included use of urine-circulating cathodic antigen (CCA) dipsticks and coproscopy of stool with duplicate Kato-Katz (K-K) thick smears. Prevalence of intestinal schistosomiasis by urine-CCA was 67.4% (95% confidence

literature Review	We have designed an individually randomised, parallel group trial of intensive versus standard praziquantel (PZQ) intervention against schistosomiasis, to determine effects on vaccine response outcomes among school-going adolescents (9-17 years) from rural Schistosoma mansoni-endemic Ugandan islands. Vaccines to be studied comprise BCG on day 'zero'; yellow fever, oral typhoid and human papilloma virus (HPV) vaccines at week 4; and HPV and tetanus/diphtheria booster vaccine at week
Randomized controlled trial	28. The intensive arm will receive PZQ
	Results: Adequate dosing can be achieved with formulations that can be split into four 150 mg quarters for children weighing 5 kg or more, and with tablets than can be split into two 300 mg halves for children weighing 10 kg or more. Giving ½ tablet for 5-7 kg; ¾ tablet for 8-10 kg; 1 tablet for 11-15 kg; 1 ½ tablet for 16-21 kg; and two tablets for 22-25 kg will have 100% of subjects correctly dosed
systematic review	within the target 40-60 mg/kg range.

Cohort Study	Schistosomiasis control programs are designed to reduce morbidity by providing mass drug administration (MDA) of praziquantel to at-risk populations. We compared morbidity markers between two cohorts of Kenyan schoolchildren that initially had high prevalence of Schistosoma mansoni infections. One cohort (N = 416 at year 1) received four rounds of annual MDA in a community-wide treatment (CWT) strategy. The other cohort (N = 386 at year 1) received school-based treatment (SBT) every other year over the 4-year period. We measured infection with S. mansoni and soil-transmitted helminths (STH) as well as subtle morbidity markers at year 1, year 3, and year 5 and compared cohorts with
	Schistosomiasis affects over 200 million people worldwide, most of whom are children. Research and control strategies directed at preschool-aged children (PSAC), i.e., ≤5 years old, have lagged behind those in older children and adults. With the recent WHO revision of the schistosomiasis treatment guidelines to include PSAC, and the recognition of gaps in our current knowledge on the disease and its treatment in this age group, there is now a concerted effort to address these shortcomings. Global and national
Cross sectional study	during the study, 76.1% of participants claimed to know about schistosomiasis ($p<0.001$) among them, 85.6% did not know the mode of contamination ($p=0.001$) and 66.3% knew the traditional treatment ($p=0.004$). Participants whose households were close to water impoundment were 2.16 times more likely to know schistosomiasis than those who were not (95% CI = [1.49 - 3.11]).

Molluscicides spearheaded control programmes until the late 1970s but were then replaced by the newly developed, safe drugs still used today. Whatever the method used, the initial goal of eradication was, in the light of experience and cost, gradually replaced by less ambitious targets; first to stop transmission and then to reduce morbidity. The most successful programmes combined several methods to minimise reinfection after chemotherapy. Comparisons between different

Until recently, the epidemiology and control of schistosomiasis in sub-Saharan Africa have focused primarily on infections in school-aged children and to a lesser extent on adults. Now there is growing evidence and reports of infection in infants and pre-school-aged children (≤ 6 years old) in Ghana, Kenya, Mali, Niger, Nigeria and Uganda, with reported prevalence from 14% to 86%. In this review, we provide available information on the epidemiology, transmission and control of schistosomiasis in this age group, generally not considered or included in national schistosomiasis control programmes that are being implemented in several sub-Saharan African countries.

Overall, [illegible] AEs were reported by [illegible] children; 91.3%, 8.4%, and 0.3% of the AEs were mild, moderate, and severe, respectively, and most resolved within 3 days. Headache (21%), dizziness or fainting (15.2 %), nausea (12.8%) and stomach pain (12.2%) were the most common AEs. The overall cumulative incidence of experiencing at least one type of AE was 20.6% (95% confidence interval [CI] 19.7-21.5%), being significantly higher ($p < 0.001$) in children with pre-MDA clinical events (27.5%,

Surveillance study

Systematic Review	Control programmes generally use a school-based strategy of mass drug administration to reduce morbidity of schistosomiasis and soil-transmitted helminthiasis (STH) in school-aged populations. The success of school-based programmes depends on treatment coverage. The community-directed treatment (ComDT) approach has been implemented in the control of onchocerciasis and lymphatic filariasis in Africa and improves treatment coverage. This study compared the treatment coverage between the ComDT approach and the school-based treatment approach, where non-enrolled school-aged children
Systematic Review	The question of whether ecology (age-dependant exposure) or immunity (resistance to reinfection), or some combination of both, determines the form of observed convex age-intensity profile is still unresolved, but there is a growing body of evidence that the human hosts acquire some partial level of immunity after a long period of repeated exposure to infection. In the majority of past research modelling schistosome transmission and the impact of MDA programmes, the effect of acquired immunity has not been taken into account. Past work has been
`Narrative review	The São Tomé e Príncipe government is committed to achieving neglected tropical disease (NTD) control and elimination as a public health problem by 2025. In 2014, the Ministry of Health led a national survey to determine the prevalence of soil-transmitted helminths (STHs) and schistosomiasis across the country. Following this survey, a preventive
systematic review	Although schistosomiasis has recently attracted increased focus and funding for control, it has been estimated that less than 20% of the funding needed to control the disease in Africa is currently available. In this article the following issues are discussed: the rationale, development and objectives of the Schistosomiasis Control Initiative (SCI)-supported programmes; the management approaches followed to

[illegible]
of meetings to specify empirical questions and design studies related to different schedules of PCT for schistosomiasis control in communities with high (gaining control studies) and moderate (sustaining control studies) prevalence of Schistosoma infection among school-aged children. Seven studies are currently being implemented in five African countries. During the first year, villages were screened for eligibility, and data were collected on prevalence and intensity of infection prior to randomisation and the

Since 2004 the West African countries of Burkina Faso, Mali and Niger have implemented national schistosomiasis and soil-transmitted helminthiasis control programmes with financial and technical support from the Schistosomiasis Control Initiative (SCI). In the first three years of the control programmes, nearly 13.5 million doses of praziquantel and

The study was conducted on 400 children; 103 PSAC and 297 SAC. Diagnosis of Schistosoma mansoni was based on triplicate Kato-Katz thick smears from a single stool sample. To identify the missed cases by Kato-Katz, 120 randomly selected negative cases (38 PSAC and 82 SAC) were screened by real-time PCR. All S. mansoni-positive cases by Kato-

Randomized Trial

A total of 150 villages were randomized into six treatment arms (25 villages per arm), were assessed at baseline, and received two or four rounds of MDA using community-wide (CWT) or school-based (SBT) treatment over 4 years. In the fifth year, a final evaluation was conducted. The primary outcomes were prevalence and intensity of Schistosoma

Herein, we summarize what we consider are major contributions resulting from the Schistosomiasis Consortium for Operational Research and Evaluation (SCORE) program, including its key findings and key messages from those findings. Briefly, SCORE's key findings

[illegible]
parasitic disease affecting more than 200 million people worldwide. Direct contact with snail-infested freshwater is the primary route of exposure. Water management infrastructure, including dams and irrigation schemes, expands snail habitat, increasing the risk across the landscape. The Diama Dam, built on the lower basin of the Senegal River to
We searched MEDLINE, Embase and Web of Science to identify papers that reported schistosome prevalence before and after praziquantel administration, either to children only or to all community members. Extracted data included Schistosoma species, drug administration strategy, number of treatment rounds, follow-up interval and prevalence and intensity before and after treatment. We
We propose a nonlinear snail force of infection (FOI) that takes into account an intermediate larval stage (miracidium) and snail biology. We focused, in particular, on the effects of snail force of infection (FOI) on the impact of mass drug administration (MDA) in human communities. The
[illegible]
control schistosomiasis in sub-Saharan Africa have had limited success, primarily because they fail to engage with the social, political, economic and ecological contexts in which they are delivered. Despite the call to foster community engagement and to adapt interventions to local
[illegible]
of meetings to specify empirical questions and design studies related to different schedules of PCT for schistosomiasis control in communities with high (gaining control studies) and moderate (sustaining controlstudies) prevalence of Schistosoma infection among school-aged children. Seven studies are currently being

Uptake of praziquantel reduced from 93.9 to 78.0 % (p = 0.002) in the snack schools but was unchanged in the non-schools 78.7 and 70.4 % (p = 0.176). The occurence of side-effects attributable to praziquantel increased from 34.4 to 61.2 % (p = 0.001) in the snack schools but was unchanged in the non-snack schools; 46.9 and 53.2 % (p = 0.443). Although the prevalence of S. mansoni infection

Since 2004 the West African countries of Burkina Faso, Mali and Niger have implemented national schistosomiasis and soil-transmitted helminthiasis control programmes with financial and technical support from the Schistosomiasis Control Initiative (SCI). In the first three years of the control programmes, nearly 13.5

indicate that 81% of the villages in Plateau and Nasarawa states probably qualify for the mass administration of praziquantel (PZQ) because of Schistosoma haematobium (SH) and/or S. mansoni (SM) infection. To determine the best strategy, relative costs were modelled for four different programmatic approaches to mass drug administration (MDA) at village level. The approaches considered

Passive case finding based on adequate diagnosis and treatment of symptomatic individuals with praziquantel by the health care facilities is a minimum requirement for integrated schistosomiasis control. Two field studies were conducted in Ghana to obtain quantifications about the steps in this process: (1) a study of health-

Subjects: Schistosomiasis prevention & control; Anthelmintics administration & dosage; Child; Global Health; Humans; Praziquantel administration & dosage; Schistosomiasis drug therapy

	Following a 3-week period of mass treatment in six communities, we observed an increased awareness of rivers/streams as a source of schistosomiasis. In addition, the communities reported overwhelming support for the mass treatment after receiving treatment. The reasons for this acceptance appear to include the perceived efficacy of praziquantel against the disease and its availability free of charge. The drug's distributors adhered to the originally specified systems-based protocol for mass prevalent parasitic diseases in developing countries. After malaria, schistosomiasis is the most important tropical disease in terms of human morbidity with significant economic and public health consequences. Although schistosomiasis has recently attracted increased focus and funding for
	Schistosomiasis is a helminthiasis infecting approximately 250 million people worldwide. In 2001, the World Health Assembly (WHA) 54.19 resolution defined a new global strategy for control of schistosomiasis through preventive chemotherapy programmes. This
medline	Overall, 3196 AEs were reported by 1658 children; 91.3%, 8.4%, and 0.3% of the AEs were mild, moderate, and severe, respectively, and most resolved within 3 days. Headache (21%), dizziness or fainting (15.2 %), nausea (12.8%) and stomach pain (12.2%) were the most common AEs. The overall cumulative incidence of experiencing at least one type of AE was 20.6% (95% confidence interval [CI] 19.7-21.5%), being significantly higher ($p < 0.001$) in children
cross sectional study medline	A quantitative cross-sectional survey was carried out among 649 school-age children in the Mtama district to determine the burden and factors associated with continuity of S.haematobium infection transmission. A single urine specimen was obtained from each pupil and tested for macro- and microhaematuria, presence of S.haematobium ova, as well intensity of

randomized trial	Overall, 7,410 children aged 9-12 years were examined at baseline and 7,223 at the final survey. The baseline prevalence of S. mansoni was 17.4%, 20.2%, and 25.2% in arms 1, 2, and 3, respectively. In the final year, we observed the lowest prevalence of 10.4% in arm 1, compared to 18.2% in arm 2 and 17.5% in arm 3. The comparison between arms 1 and 2 estimated an odds ratio (OR) of 0.52 but
randomized non-infiriority clinical trial	t 3 weeks of post-treatment, cure rates were 88.3% (263/298, 95% CI = 84.1%- 91.4%) and 81.2% (277/341, 95% CI = 76.7%- 85.0%) for the combination therapy and praziquantel alone, respectively ($p < 0.01$, odds ratio (OR) = 1.74, 95% CI of OR = 1.11 to 2.69). At 8 weeks, there was a significant drop in the
	Operational Research and Evaluation (SCORE) was created to conduct research that could inform programmatic decision-making related to schistosomiasis. SCORE included several large cluster randomized field studies involving mass drug
	Herein, we summarize what we consider are major contributions resulting from the Schistosomiasis Consortium for Operational Research and Evaluation (SCORE) program, including its key findings and key messages from those findings. Briefly, SCORE's key findings are as follows: i) biennial mass drug administration (MDA) with praziquantel can control schistosomiasis to moderate
	Fifteen articles met our inclusion criteria. In general, it was hard to compare the reported costs from the different studies due to different approaches used to estimate and classify the costs of the intervention assessed. Costs varied considerably from one study to another, ranging from US0.06toUS 4.46 per person treated. The difference between financial and opportunity costs only played

	Generalized linear models with variable selection possessed relatively stable performance compared with tree-based methods. Models applied to Kenya data alone or combined data from Kenya and Tanzania could reach over 80% predictive accuracy, whereas predicting PHS for Tanzania was challenging. Models developed from one country and validated
cluster randomized trial	We conducted a cluster randomized trial comparing the target population and timing of mass drug administration (MDA) with praziquantel for control of schistosomiasis in villages in western Kenya with high initial prevalence (> 25%) according to a harmonized protocol developed by the
	Annual treatment reduced the prevalence of S. haematobium infection ($p < 0.05$) from 23.1% at baseline to 0.47% after 2 years. Overall cure rate was 97.8%. Intensity of infection declined ($p < 0.05$) from 15.9 eggs/10 ml urine at baseline to 2 eggs/10 ml urine. After two years, overall
systematic review	The search yielded [illegible] literatures of which 93 full text research, review and online articles were deemed fit for inclusion. Our key findings showed that: (1) of all World Health Organization (WHO) Regions, Africa is the most endemic zone for US, with Kenya and Senegal recording the

ıe prevalence and intensity of STH and schistosomiasis infection were significantly reduced comp

of S. haematobium had decreased significantly in the cohort at 2 years post praziquantel treatment, during a

revealed dose-independent efficacy against light infections of S. haematobium. Over the dose range tested, praziquantel displayed a ceiling effect with the highest

This suggests that a combination of environmental and mass treatment has had a significant impact on transmission in Lango

None of the parameters analyzed completely disappeared. After one single course of treatment with praziquantel, all the analyzed parameters

The national schistosomiasis control programme (NSCP) adopted a new elimination strategy by readjusting thresholds for MDA using praziquantel and targeting all

S. haematobium infections and reinfections are seasonal and depend on micro-geographical settings. The risk of being infected with schistosomes in pre-school aged children increases with

Observed proportions of faecal epg intensities were light (78.6%), moderate (17.9%) and heavy (3.6%). Soil-transmitted helminthiasis was noted, prevalence of ascariasis was 18.8% and

[illegible]
reveals three key findings: 1) rural villagers understand schistosomiasis risk (i.e., where and when infections occur), 2)

Formulations that can be divided into four parts (to give 150 mg increments) are preferred for children weighing less than 11 kg; the same dosing can be applied with 600 mf

Nevertheless, by year 5, children in both cohorts demonstrated significant decreases in wasting, ultrasound-detected organomegaly, and STH infection along with significantly improved pediatric quality-of-life

In this review, we (i) discuss the current knowledge on the dynamics and consequences of paediatric schistosomiasis and (ii) identify knowledge and policy

majority of participants reported being aware of schistosomiasis. However, the modes of transmission, prevention, and treatment of schistosomiasis were not well known.

Drug resistance on a scale comparable with malaria has not occurred in schistosomiasis but the likely withdrawal of all drugs except [illegible]

previous assumptions, we show that schistosomiasis infection starts from early childhood in many endemic communities and factors associated with exposure of infants and pre-school-aged children to infection are yet to be

Praziquantel and albendazole MDA is safe and well-tolerated; however, one in five children experience transient mild to moderate, and in few cases severe,

Similar treatment coverage levels were attained at the second treatment round. Again, equal levels of treatment coverage were found between the two approaches for the enrolled school-aged
Compared with scenarios with no immunity, we find that acquired immunity makes the MDA programme less effective with a slower decrease in the prevalence of
This narrative review systematises the existing literature reporting on the epidemiology of NTDs for
The results have demonstrated that morbidity due to schistosomiasis has been reduced by the control programmes.

These studies of different treatment schedules with PZQ will provide the most comprehensive data thus far on the optimal frequency and continuity of PCT for schistosomiasi

The challenges currently faced by these national control programmes are the ability to maintain the reduction in

The prevalence of S. mansoni with Kato-Katz was 7.8% among PSAC and 7.4% among SAC. Most of children

Combined arms of villages that received four rounds of treatment had greater reduction than villages in

Besides providing useful information for program managers and revision of

Our analysis reveals three key findings: 1) rural villagers understand schistosomiasis risk (i.e., where and when

The results of this meta-analysis do not support the hypothesis that community-wide treatment is more effective than targeted

The observed impact of varying location-specific snail inputs sheds light on the

interventions included the implementation of novel school-based education and training, the

studies of different treatment schedules with PZQ will provide the most comprehensive

Our results show that in absence of food, uptake of praziquantel reduced and the side-effects of the drug increased. However, the

Our new working hypothesis is that targeted control accompanied by periodic mass treatment

year costs, to cover a population of 30,000, were U.S.18,673for themodelwithscreeningonlyforSH,U.S.36,816 for the model with

Programmes aimed at making the drug available at all levels of the health care delivery system and

Obtaining community support and involvement before the implementation of mass treatment of schistosomiasis with praziquantel contributes to have demonstrated that morbidity due to schistosomiasis has been reduced by the

[illegible] written by the 2018-2022 Schistosomiasis Guidelines Development Group and its Praziquantel and albendazole MDA is safe and well-tolerated; however, one in five children experience transient mild to moderate, and in few

[illegible] S.haematobium infection by macro- and microhaematuria was 13.1% and 46.2% respectively. The prevalence

The three treatment schedules investigated led to a reduction in the prevalence and intensity of S. mansoni infection
Praziquantel and Dihydroartemisinin piperaquine combination therapy is safe, and more
there was often a wide variation behind these summary statistics, and
The data and specimens collected and curated through SCORE efforts will continue to be critical resource for
The degree of transparency of most of the costing studies of schistosomiasis interventions found in the current review was limited.

Statistical
models applied
to Year-3 data
could help
predict PHS
and guide
program
decisions, with
infection

Combined
arms of
villages that
received four
rounds of
treatment had
greater

Annual
praziquantel
treatment
delivered to
school
children over 2
years

In view of the
WHO strategic
plan to
eliminate
schistosomiasi
s by 2020 and
the findings

pared to baseline. Data on STH indicate that stopping MDA in areas with high baseline prevalenc

:e may result in significant rebound of infection. Togo's findings may help refine treatment recom

mendations for these diseases.
