# Supplementary material for: Effects of paediatric schistosomiasis control programmes in sub-Saharan Africa: A systematic review
Source: PLoS One. 2024 May 2;19(5):e0301464. doi: 10.1371/journal.pone.0301464 (PMC11065241; doi:10.1371/journal.pone.0301464)
Supplement: S2 File — (PDF) [file pone.0301464.s004.pdf]

Database	Date	Article Citation
Pubmed	2018	Kabuyaya, M., Chimbari, M. J., & Mukaratirwa, S. (2018). Infection status and risk factors associated with urinary schistosomiasis among school-going children in the Ndumo area of uMkhanyakude District in KwaZulu-Natal, South Africa two years post-treatment. International journal of infectious diseases : IJID : official publication of the International Society for Infectious Diseases, 71, 100–106. https://doi.org/10.1016/j.ijid.2018.04.002
	2018	Coulibaly, J. T., Panic, G., Yapi, R. B., Kovač, J., Barda, B., N'Gbesso, Y. K., Hattendorf, J., & Keiser, J. (2018). Efficacy and safety of ascending doses of praziquantel against Schistosoma haematobium infection in preschool-aged and school-aged children: a single-blind randomised controlled trial. BMC medicine, 16(1), 81.
		Adriko, M., Tinkitina, B., Tukahebw, E. M., Standley, C. J., Stothard, J. R., & Kabatereine, N. B. (2018). The epidemiology of schistosomiasis in Lango region Uganda 60 years after Schwetz 1951: Can schistosomiasis be eliminated through mass drug administration without other supportive control measures?. Acta tropica, 185, 412–418. https://doi.org/10.1016/j.actatropica.2018.06.009
		Bocanegra, C., Pintar, Z., Mendioroz, J., Serres, X., Gallego, S., Nindia, A., Aznar, M. L., Soriano-Arandes, A., Salvador, F., Gil, E., Sikaleta, N., Moreno, M., & Molina, I. (2018). Ultrasound Evolution of Pediatric Urinary Schistosomiasis after Treatment with Praziquantel in a Highly Endemic Area. The American journal of tropical medicine and hygiene, 99(4), 1011–1017. https://doi.org/10.4269/ajtmh.18-0343
		[illegible] Gabrielli, A. F., Abdelhai, R., Hashish, A., Jabbour, J., & Ramzy, R. M. R. (2018). Elimination of schistosomiasis haematobia as a public health problem in five governorates in Upper Egypt. Acta tropica, 188, 9–15. https://doi.org/10.1016/j.actatropica.2018.08.024
		Mutsaka-Makuvaza, M. J., Matsena-Zingoni, Z.,

Sheehy, C., Lawson, H., Andriamasy, E. H.,
Lund, A. J., Sam, M. M., Sy, A. B., Sow, O. W.,
Nkurunungi, G., Zirimenya, L., Nassuuna, J.,
Olliaro, P. L., Vaillant, M., Hayes, D. J., Montresor,
Sircar, A. D., Mwinzi, P. N. M., Onkanga, I. O.,
Osakunor, D. N. M., Woolhouse, M. E. J., &
Maïga, F. K., Sangare, M., Dolo, H., Dicko, I.,
Sturrock R. F. (2001). Schistosomiasis epidemiology
Ekpo, U. F., Oluwole, A. S., Abe, E. M., Etta, H. E.,
Kabatende, J., Barry, A., Mugisha, M., Ntirenganya,
Massa, K., Olsen, A., Sheshe, A., Ntakamulenga,
Kura, K., Hardwick, R. J., Truscott, J. E., &
Byrne, A., Rosário, A., da Conceição Ferreira, M.,
Fenwick, A., Webster, J. P., Bosque-Oliva, E., Blair,
Ezeamama, A. E., He, C. L., Shen, Y., Yin, X. P.,
Garba, A., Touré, S., Dembelé, R., Boisier, P.,
Allam, A. F., Salem, A., Elsheredy, A., Dewair, M.
Secor WE, Wiegand RE, Montgomery SP, Karanja

Ebscohost	04/03/2023	Colley DG; Fleming FM; Matendechero SH; Knopp
		Lund AJ; Sam MM; Sy AB; Sow OW; Ali S;
	medline	Cribb DM; Clarke NE; Doi SAR; Vaz Nery S, PLoS

medline

Gurarie D; Lo NC; Ndeffo-Mbah ML; Durham DP;
Person B; Knopp S; Ali SM; A'kadir FM; Khamis
Ezeamama AE; He CL; Shen Y; Yin XP; Binder SC;
Muhumuza S; Olsen A; Katahoire A; Nuwaha F,
Garba A; Touré S; Dembelé R; Boisier P; Tohon Z;
Gutman J; Richards FO Jr; Eigege A; Umaru J;
de Vlas SJ; Danso-Appiah A; van der Werf MJ;
Taylor M, Bulletin of the World Health Organization
Adeneye AK; Akinwale OP; Idowu ET; Adewale B;
Fenwick A; Webster JP; Bosque-Oliva E; Blair L;
Lo NC; Bezerra FSM; Colley DG; Fleming FM;
Kabatende J; Barry A; Mugisha M; Ntirenganya L;
Mushi V; Zacharia A; Shao M; Mubi M; Tarimo D,
Ouattara M; Diakité NR; Yao PK; Saric J; Coulibaly
Mnkugwe RH; Minzi O; Kinung'hi S; Kamuhabwa
Binder S; Campbell CH; Castleman JD; Kittur N;
By: Colley DG; Fleming FM; Matendechero SH;
Schistosomiasis, a disease caused by blood flukes of

Shen Y; Sung MH; King CH; Binder S; Kittur N;
Whalen CC; Colley DG, The Journal of infectious
diseases [J Infect Dis], ISSN: 1537-6613, 2020 Feb
18; Vol. 221 (5), pp. 796-803; Publisher: Oxford
University Press; PMID: 31621850;

Secor WE; Wiegand RE; Montgomery SP; Karanja DMS; Odiere MR, The American journal of tropical medicine and hygiene [Am J Trop Med Hyg], ISSN: 1476-1645, 2020 Feb; Vol. 102 (2), pp. 318-327; Publisher: American Society of Tropical Medicine and Hygiene; PMID: 31802733;

Chisango TJ; Ndlovu B; Vengesai A; Nhidza AF; Sibanda EP; Zhou D; Mutapi F; Mduluza T, BMC infectious diseases [BMC Infect Dis], ISSN: 1471-2334, 2019 Mar 04; Vol. 19 (1), pp. 219; Publisher: BioMed Central; PMID: 30832614;

Atalabi TE; Adubi TO, BMC infectious diseases
[BMC Infect Dis], ISSN: 1471-2334, 2019 Jan 18;
Vol. 19 (1), pp. 73; Publisher: BioMed Central;
PMID: 30658583;

Study Type	Summary of study results
2 year-Cohort study	Of the 173 participants screened 2 years post-treatment, 10 were infected. Six of these were new infection cases, while four were cases of re-infection. The intensity of infection had decreased significantly (p = 0.001) at the time of the follow-up survey compared to the baseline survey. However, no significant difference was found among the risk factors for schistosomiasis 2 years later
Randomised dose finding trial	treatment. Almost 90% of PSAC and three quarters of SAC were lightly infected with S. haematobium. Follow-up data were available for 157 PSAC and 166 SAC. In PSAC, CRs of praziquantel were 85.7% (30/35), 78.0% (32/41) and 68.3% (28/41) at 20, 40 and 60 mg/kg and 47.5% (19/40) for placebo. In SAC, CRs were 10.8% for placebo (4/37), 55.6% for 20 mg/kg (25/45), 68.3% for 40 mg/kg (28/41) and 60.5% for 60 mg/kg (26/43). ERRs based on geometric means ranged between 96.5% (60 mg/kg)
Systematic Review	Contrary to earlier records, S. haematobium was low and confined to a few putative foci, with declined in infections from 28.2% in 1951 to 2.48% by 2011. Although this decline can be attributed to control, this was already much lower in 1967 than 1951, long before control interventions began suggesting that environmental changes may have made the habitat less suitable for the transmission of S. haematobium. Compared to the historical records S. mansoni prevalence first increased up immediately before control interventions in 2003, significantly declined
Epidemiological study	Seventy children who had previously participated in an epidemiological study on schistosomiasis in the city of Cubal, Angola, and had also performed urinary ultrasound between August 2013 and February 2014 were cited 6-8 months later to assess the possible reinfection and repeat new urinary ultrasound, analyzing changes at the level of urinary pathology. The presence of hematuria and proteinuria was also analyzed. Of the 70 children analyzed, 29 (41.4%) were girls, with an average age of 10.4 years
	No subject with heavy intensity of infection was detected in Fayoum and Bani Sweif governorates. Of the 39 studied districts 97.4% had prevalence of heavy intensity infection of <1%, indicating elimination of schistosomiasis haematobia as a public health problem in these districts. Of those studied 72.0% were male. Males were 2.9 times as likely to
12 month Compliance	Of the 535 children recruited from the five

A pilot survey	School-aged children (SAC) have a considerable
literature Review	
Randomized controlled	We have designed an individually randomised,
systematic review	Results: Adequate dosing can be achieved with
Cohort Study	Schistosomiasis control programs are designed to
	Schistosomiasis affects over 200 million people
Cross sectional study	during the study, 76.1% of participants claimed to
	Molluscicides spearheaded control programmes until
	Until recently, the epidemiology and control of
Surveillance study	Overall, 3196 AEs were reported by 1658 children;
Systematic Review	Control programmes generally use a school-based
Systematic Review	The question of whether ecology (age-dependant
`Narrative review	The São Tomé e Príncipe government is committed to
systematic review	Although schistosomiasis has recently attracted
	Beginning in 2009, SCORE held a series of meetings
	Since 2004 the West African countries of Burkina
	The study was conducted on 400 children; 103
Randomized Trial	A total of 150 villages were randomized into six
	[illegible] district, Uganda, 12 primary schools were randomized into two groups; one received education messages for schistosomiasis prevention for two months prior to mass treatment, while the other, in addition to the education messages, received a pre-treatment snack shortly before mass treatment. Four weeks after mass treatment, uptake of praziquantel was assessed among a random sample of 595 children in the snack schools and 689 children in the non-snack schools as the primary outcome. The occurrence of side effects and the prevalence and mean intensity of Schistosoma mansoniinfection were determined as the secondary outcomes. Uptake of praziquantel was higher in the snack schools, 93.9% (95% CI 91.7%–95.7%), compared to that in the non-snack schools, 78.7% (95% CI 75.4%–81.7%) (p = 0.002). The occurrence of side effects was lower in the snack schools, 34.4% (95% CI 31.5%–39.8%), compared to that in the non-snack schools, 46.9% (95% CI 42.2%–50.7%) (p = 0.041). Prevalence and mean intensity of S. mansoniinfection was lower in the snack schools, 1.3% (95% CI 0.6%–2.6%) and 38.3 eggs per gram of stool (epg) (95% CI 21.8–67.2), compared to that in the non-snack schools, 14.1% (95% CI 11.6%–16.9%) (p = 0.001) and 78.4 epg (95% CI 60.6–101.5) (p = 0.001), respectively.
Randomized Trial	Herein, we summarize what we consider are major
	Human schistosomiasis is a snail-borne parasitic
	We searched MEDLINE, Embase and Web of

	We propose a nonlinear snail force of infection (FOI)
	Top-down biomedical interventions to control
	Beginning in 2009, SCORE held a series of meetings
	Uptake of praziquantel reduced from 93.9 to 78.0 %
	Since 2004 the West African countries of Burkina
	The results of previous studies in Nigeria indicate
	Passive case finding based on adequate diagnosis and
	Subjects: Schistosomiasis prevention & control;
	Following a 3-week period of mass treatment in six
	Schistosomiasis remains one of the most prevalent
	Schistosomiasis is a helminthiasis infecting
medline	Overall, 3196 AEs were reported by 1658 children;
cross sectional study	A quantitative cross-sectional survey was carried out
randomized trial	Overall, 7,410 children aged 9-12 years were
randomized non-	t 3 weeks of post-treatment, cure rates were 88.3%
	The Schistosomiasis Consortium for Operational
	Herein, we summarize what we consider are major
	Fifteen articles met our inclusion criteria. In general,
	Generalized linear models with variable selection possessed relatively stable performance compared with tree-based methods. Models applied to Kenya data alone or combined data from Kenya and Tanzania could reach over 80% predictive accuracy, whereas predicting PHS for Tanzania was challenging. Models developed from one country and validated in another failed to achieve satisfactory performance. Several Year-3 variables were identified as key predictors.

We conducted a cluster randomized trial comparing the target population and timing of mass drug administration (MDA) with praziquantel for control of schistosomiasis in villages in western Kenya with high initial prevalence (> 25%) according to a harmonized protocol developed by the Schistosomiasis Consortium for Operational Research and Evaluation. A total of 150 villages were randomized into six treatment arms (25 villages per arm), were assessed at baseline, and received two or four rounds of MDA using community-wide (CWT) or school-based (SBT) treatment over 4 years. In the fifth year, a final evaluation was conducted. The primary outcomes were prevalence and intensity of Schistosoma mansoni infections in children aged 9-12 years, each year their village received MDA. Baseline and year 5 assessments of first-year students and adults were also performed. Using Poisson and negative binomial regression with generalized estimating equations, we found similar effects of CWT and SBT MDA treatment strategies in children aged 9-12 years: significant reductions of prevalence of infection in all arms and of heavy-intensity (≥ 400 eggs/gram) infections in most arms

cluster randomized trial but no significant differences between arms.

Annual treatment reduced the prevalence of S. haematobium infection ($p < 0.05$) from 23.1% at baseline to 0.47% after 2 years. Overall cure rate was 97.8%. Intensity of infection declined ($p < 0.05$) from 15.9 eggs/10 ml urine at baseline to 2 eggs/10 ml urine. After two years, overall rate of reinfection was 0.96%. At baseline, total IgG4 was higher in S. haematobium-infected children ($p = 0.042$) ,while all other immunoglobulins were within normal ranges. There was an increase in total IgG2 ($p = 0.044$) levels and a decrease in total IgG4 ($p = 0.031$) levels 2 years post-treatment; and no significant changes in other total immunoglobulins. Schistosoma-infected children at baseline showed an increase in anti-Sh13 IgG1 ($p = 0.005$) and a decrease in Sh13 IgG4 levels ($p = 0.012$) following treatment.

The search yielded 183 literatures of which 93 full text research, review and online articles were deemed fit for inclusion. Our key findings showed that: (1) of all World Health Organization (WHO) Regions, Africa is the most endemic zone for US, with Kenya and Senegal recording the highest prevalence and mean intensity respectively; (2) SAC within the range of 5-16 years contribute most significantly to the transmission cycle of US globally; (3) gender is a factor to watch out for, with male often recording the highest prevalence and intensity of infection; (4) contact with open, potentially infested water sources contribute significantly to transmission; (5) parental factors (occupation and education status) predispose SAC to US; (6) economic vis a vis ecological factors play a key role in infection transmission; and (7) in the last decade, a treatment coverage of 45% was never achieved globally for SAC or non-SAC treatment category for urinary schistosomiasis.

systematic review

Comment

The prevalence of S. haematobium had decreased significantly in the cohort at 2 years post praziquantel treatment, during a period of persistent drought in the area. Risk factors that were significantly associated with schistosomiasis at baseline were no longer significantly associated at 2 years following treatment

Praziquantel revealed dose-independent efficacy against light infections of S. haematobium. Over the dose range tested, praziquantel displayed a ceiling effect with the highest response for 20 mg/kg in PSAC. In SAC maximum efficacy was obtained with 40 mg/kg praziquantel. Further investigations are required in children with moderate to heavy infections.

This suggests that a combination of environmental and mass treatment has had a significant impact on transmission in Lango region.

None of the parameters analyzed completely disappeared. After one single course of treatment with praziquantel, all the analyzed parameters showed regression. Improvement was greater in the urinary bladder than in the upper urinary tract, though these lesions also reversed; the reversion of all parameters was greater among children older than 10 years old than the younger ones.

The national schistosomiasis control programme (NSCP) adopted a new elimination strategy by readjusting thresholds for MDA using praziquantel and targeting all transmission areas. The NSCP, after this major achievement of elimination of schistosomiasis

S. haematobium infections and

Observed proportions of faecal epg
Our analysis reveals three key findings:

Formulations that can be divided into
Nevertheless, by year 5, children in both
In this review, we (i) discuss the current
most of the majority of participants
Drug resistance on a scale comparable
Contrary to previous assumptions, we
Praziquantel and albendazole MDA is
Similar treatment coverage levels were
Compared with scenarios with no
This narrative review systematises the
The results have demonstrated that
These studies of different treatment
The challenges currently faced by these
The prevalence of S. mansoni with Kato-
Combined arms of villages that received

Our results suggest that provision of a pre-treatment snack combined with education messages achieves a higher uptake compared to the education messages alone. The use a pre-treatment snack was associated with reduced side effects as well as decreased prevalence and intensity of S. mansoni infection.
Besides providing useful information for
Our analysis reveals three key findings:
The results of this meta-analysis do not

The observed impact of varying location-
These interventions included the
Results: These studies of different
Our results show that in absence of
Our new working hypothesis is that
The total 5-year costs, to cover a
Programmes aimed at making the drug

Obtaining community support and
The results have demonstrated that
This Review, written by the 2018-2022
Praziquantel and albendazole MDA is
Prevalence of S.haematobium infection 52.7%
The three treatment schedules
Praziquantel and Dihydroartemisinin
However, there was often a wide
The data and specimens collected and
The degree of transparency of most of

Statistical models applied to Year-3 data could help predict PHS and guide program decisions, with infection intensity, prevalence of heavy infections (≥400 eggs/gram of feces), and total prevalence being particularly important factors. Additional studies including more variables and locations could help in developing generalizable models.

Combined arms of villages that received four rounds of treatment had greater reduction than villages in arms that only received two rounds of treatment. Surprisingly, we also found benefits of SBT for first-year primary students and adults, who never received treatment in those arms. Our data support the use of annual SBT for control programs when coupled with attention to infections in younger children and occasional treatment of adults., Database: MEDLINE

Annual praziquantel treatment delivered to school children over 2 years significantly reduce prevalence, intensity of infection and reinfection of S. haematobium infection. Treatment was also observed to cause a reduction in schistosome-specific blocking IgG4 and an increase in Schistosoma-specific protecting IgG1., Database: MEDLINE

In view of the WHO strategic plan to eliminate schistosomiasis by 2020 and the findings from this review, it is obvious that this goal, in the face of realities, might not be achieved. It is imperative that annual control programmes be scaled up marginally, particularly in the African region of WHO. While US-based researches should be sponsored at the grass-root level to unveil hidden endemic foci, adequate facilities for Water, Sanitation, and Hygiene (WASH) should be put in place in all schools globally., Database: MEDLINE
