# Supplementary material for: Effects of paediatric schistosomiasis control programmes in sub-Saharan Africa: A systematic review
Source: PLoS One. 2024 May 2;19(5):e0301464. doi: 10.1371/journal.pone.0301464 (PMC11065241; doi:10.1371/journal.pone.0301464)
Supplement: S3 File — (PDF) [file pone.0301464.s005.pdf]

SN	First author	Country	Sample size	Age group<br><br>(Years)	Control strategy	*Schistosoma* species
1	(Chisango et al., 2019)	Zimbabwe	212<br>105 boys<br>107 girls	SAC<br>7-13 years	MDA	*S. haematobium*<br>*S. mansoni*
2	(Karanja et al., 2017a)	Kenya	7500 SAC from 75 schools<br>cohort	SAC<br>9-12years	MDA	*S. mansoni*
3	(Secor et al., 2019)	Kenya	100 individuals from 25 villages:<br>6 arms with 25 villages each<br>cohort	SAC<br>9-12 years	MDA	*S. mansoni*
4	(Olsen et al., 2018a)	Tanzania	14620 SAC	SAC<br>13-14 years	MDA	*S. mansoni*
5	(Hessler et al., 2017)	Cote d'Ivoire	111 individuals<br>60 females<br>50 males	SAC, Adolescents<br>7-15 years	MDA	*S. mansoni*
6	(Kasambala et al., 2022b)	Zimbabwe	27 cases<br>106 controls	PSAC	MDA	*S. mansoni*<br>*S. haematobium*
7	(J. E. T. Grimes et al., 2016b)	Ethiopia	80475 children, sex matched, from 1645 schools	SAC, Adolescents<br>(10-15yrs)	WASH	*S. mansoni*
8	(Zeleke et al., 2020)	Ethiopia	8002 children<br>Infected: 408<br>Uninfected:7594	SAC<br>(5-14yrs)	MDA	*S. mansoni*
9	(Senghor et al., 2016)	Senegal	777 children,<br>Infected:226<br>Uninfected:551	SAC<br>5-11 years	MDA.<br>WASH	*S. haematobium*
10	(Ouattara et al., 2022a)	Cote' d'Ivoire	6400 individuals	SAC<br>5-14 years	MDA<br>Snail control	*S. haematobium*
11	(Ouattara et al., 2021)	Cote d'Ivoire	2455 individuals<br>Infected: 618<br>Uninfected: 1837	SAC<br>9-12 years	MDA	*S. mansoni*
12	(Person et al., 2021)	Tanzania	1451 individuals<br>708 intervention<br>743 non-intervention	SAC<br>Adolescents<br>9-16 years	Education,<br>Behaviour change	*S. haematobium*
13	(Shen et al., 2019)	Kenya	1374 individuals	SAC<br>7-8 years	MDA	*S. mansoni*
14	(Spencer et al., 2022a)	Madagascar	286 individuals	SAC<br>5-14 years	Education<br>MDA	*S. mansoni*
15	(Abudho et al., 2018a)	Kenya	1110 individuals	SAC<br>Adolescents<br>6-17 years	MDA	*S. mansoni*

16	(Trippler et al., 2021a)	Tanzania	9700 individuals	SAC<br>9-12 years	MDA<br>Snail control<br>Behaviour change	*S. haematobium*
17	(Adewale et al., 2018a)	Nigeria	434 individuals<br>108 infected<br>326 uninfected	SAC,<br>Adolescents<br>5-17 years	MDA	*S. haematobium*
18	(Chisha et al., 2020)	Ethiopia	4286 SAC individuals<br>Sex matched	SAC<br>5-14 years	MDA	*S. haematobium*
19	(Mutsaka-Makuvaza et al., 2018)	Zimbabwe	535 SAC	PSAC	MDA	*S. haematobium*
20	(Kimani et al., 2018)	Kenya	400 PSC	PSAC	MDA	*S. haematobium*
21	(Jin et al., 2021a)	Sudan	1951 SAC	SAC<br>6-14 years	MDA	*S. haematobium*<br>*S. mansoni*
22	(Mazigo et al., 2022)	Tanzania	20389 individuals	PSAC<br>SAC<br>Adolescents<br>3-17 years	MDA	*S. haematobium*
23	(Enabulele et al., 2021)	Nigeria	1,267 individuals	SAC<br>Adolescents<br>10-17 years	MDA	*S, haematobium*<br>*S. mansoni*
24	(Senghor et al., 2022)	Senegal	777 individuals	SAC<br>5-11 years	MDA	*S. haematobium*
25	(Mduluza et al., 2020)	Zimbabwe	14000 individuals	SAC<br>6-15 years	MDA	*S, haematobium*<br>*S. mansoni*
26	(Berhanu et al., 2022)	Ethiopia	499 children from two schools<br>Infected: 234<br>Uninfected: 265	SAC, Adolescents<br>5-18 years	MDA	*S. mansoni*
27	(Kim et al., 2020)	Tanzania	1716 student cohort	SAC	MDA	*S, haematobium*
28	(Lemos et al., 2020)	Angola	67 children<br>Infected:47<br>Uninfected: 20	PSAC<br>SAC<br>(2 - <15yrs)	MDA	*S. haematobium*
29	(Bronzan et al., 2018)	Togo	17,100 children at 1129 schools	SAC<br>Adolescents<br>5-17 years	*MDA*	*S. haematobium*<br>*S. mansoni*
	(Ekanem et al.,		380 children	SAC		[illegible]

30	[illegible] 2017)	Niger	Infected: 55	5-14 years	*WASH*	*S. haematobium*

Diagnosis technique	Intervention (community or school-based or combined)
Urine filtration<br>Kato Katz<br>ELISA	School-based
Kato Katz	School-based
Kato Katz	Community-based<br>School-based
Kato Katz	Community-based
Kato Katz<br>PCR	School-based
Urine filtration<br>Kato Katz	Community-based
Kato Katz	School-based
Kato Katz	Community-based
Kato Katz	School-based
Reagent strip<br>Urine filtration	School-based
Kato Katz	School-based
Urine filtration	School-based
Kato Katz<br>Abdominal Ultrasonography	School-based<br>Community-based
Kato Katz	School-based
Kato Katz	School-based

Urine filtration	School-based
Urine filtration	School-based
Urine filtration	School-based
Urine filtration	community-based
Urine filtration<br>Kato Katz	School-based
Urine filtration	School-based
Urine filtration<br>Dipstick	School-based
Urine filtration<br>Kato Katz	Community-based
Urine filtration	Community-based
Urine filtration<br>Kato Katz	School-based
Kato Katz	School-based
Urine filtration	School-based
Urine filtration	Community-based
Urine filtration<br>Kato Katz	Community-based
[illegible]	School-based

Urine filtration	Community-based

Summary of main findings
**Principal findings**
S. haematobium prevalence changed from 23.1% to 0.47%. Significant reduction in prevalence, intensity and re-infection levels after treatment. Biennial treatment kept infection levels low, chemotherapy also led to decreased levels of Ig G4 and Ig G1
Significant decreases in the prevalence and intensity of infection within the arms that received treatment every year or every other year. However, four treatments did not result in significantly lower prevalence or intensities of infection compared to treatment every other year over 5 years. Biennial treatment may have similar benefits as annual treatment in schistosomiasis control programs in moderate prevalence areas, making it possible to provide MDA to twice as many schools with the same
In all six arms, infection prevalence was significantly less at year 5 than at year 1. The prevalence of heavy-intensity infections also decreased over time, with four arms demonstrating significantly reduced levels by year 5.
Community-wide in 9- to 12-year-old schoolchildren, did not result in a significantly lower prevalence or intensities of infection compared with four times school-based treatment. Biennial treatment has the same effect as annual treatment. The significant increase in mean prevalence from years 4 to 5 observed in our study was unexpected and likely reflects increased transmission
Detection of cell-free schistosomal DNA via PCR is at least six to eight times more sensitive than any of the parasitological methods, KK, haematuria and urine filtration. It can be seen clearly from the results that after MDA, diagnosis using haematuria and egg detection missed the detection of *S. haematobium* infections. Detecting parasite eggs in the urine and stool but with low-intensity infections, especially after MDA, has been shown to have low sensitivity. PCR can detect a true positive or a true negative even when the infection load is lower as may be the case after MDA
22% - 3.6% change in prevalence due to reinfections post-treatment. PSAC with S. haematobium infection were 3.9 times more likely to have low performance in the Foundations of Learning domain in comparison to uninfected PSAC (p = 0.008). There was an improvement in the Language and Communication Domain, Eye-Hand Coordination Domain (and General
No significant differences were observed when comparing sanitation and infection with *S. mansoni.* Improving school WASH may reduce schistosomal transmission. However, different forms of WASH appear to have different effects on infection with the various parasites, with the strongest associations between water and S. mansoni.
S. mansoni progressively decreased from 9.6% to 4.1. However, a declining trend of *S. mansoni* was observed before the launch of MDA and remained constant after the start of the MDA. The positivity rate was significantly higher in males and in the 5-14 years age group. *S .mansoni* infection in school-aged children showed significant seasonal variation.
high rates of infection reduction (between 96.7 and 99.7%) were obtained. The re-infection was significantly higher in the village using the canal. Praziquantel has an impact on reducing the prevalence and intensity of urogenital schistosomiasis when administered periodically.
Baseline prevalence was 16.3% Heavy intensity infections decreased from baseline to final survey among all age groups in all arms. In the final survey, the prevalence and the AM egg count decreased in all study arms. The day after each treatment, the snails found in the human-water contact sites were all dead. B. truncates was the predominant species. Snail control did not
Treatment strategies with praziquantel investigated, achieved a reduction in prevalence and intensity of *S. mansoni* infection among children aged 9–12 years, despite reductions in prevalence observed after 5v years MDA, the prevalence (baseline 5.3% to 5.2% after intervention) remained moderately endemic in some of the treatment arms and several heavy intensity infections were observed in some of the children at the end of the intervention.
A significant association between exposure to behavioural interventions against urogenital schistosomiasis, guided by the social ecological framework and grounded in constructs of health education and improvements in knowledge about *S. haematobium* transmission and perceptions of risk; improved attitudes towards prevention and treatment of the disease with an increased uptake of swallowing of anthelminthic tablets during MDA campaigns, suggesting that a group of children had been resistant to or did not swallow the drugs in previous MDA efforts before the intervention; and self-reported changes in behaviours.
64% infected at baseline-45.5% after the intervention the study demonstrates that regular treatment of schoolchildren is associated with reductions in both *S. mansoni* infection prevalence and mean infection intensity. Health-related quality-of-life scores showed improvement in both treatment groups.
The percentage of correct pre-education answers improved between 2017 and 2018 from 53% to 72% and older children were more likely to provide correct answers. There were no differences in scores between male and female participants. Children from school education programs (SEP)-participating schools had 91% MDA attendance after the SEP—more than double the
Annual school-based MDA to lower the proportion of infected children from 44.7% at baseline to 14.0% in Year 4. Four rounds of annual PZQ MDA significantly reduced *S. mansoni* infection prevalence and virtually eliminated heavy infections as defined by WHO guidelines, supportive of the goal to prevent high-intensity schistosome infections that are generally associated with morbidity, including anaemia in children.

The apparent overall *S. haematobium* prevalence among schoolchildren decreased from 6.6% at baseline in 2012 to 3.4% in 2020 and microhaematuria levels from 9.5% to 5.2%. However, an even lower *S. haematobium* prevalence of 1.2% and microhaematuria levels. of 4.0% were observed in schoolchildren in 2019. In 2020, the considerable rebound in the overall prevalence and also infection intensity was caused by certain hotspot areas, while most areas had very low prevalence throughout the study period and also after the 16-month treatment gap. The hotspot areas showed an unstable and undulating *S. haematobium* prevalence.

A single dose of praziquantel at 40 mg/kg body weight was able to significantly reduce the prevalence and intensity of infection up to 6 months post-treatment the12th-month assessment of infection, there was an increased rate in the prevalence of infection among the children, some of whom were not found to be initially infected at baseline. This connotes new infection after treatment had started or some of the children harbour the juvenile stages of *S. haematobium* . Resurgence in the prevalence rate between 6 and 12 months post-treatment with praziquantel is herein reported and the need for a follow-up treatment in endemic

Overall treatment coverage of PZQ against SCH in the present study was 75.5%. PZQ treatment coverage among SAC was significantly higher for SAC who attended school (84.1%) compared to their counterparts(14.4%)

Prevalence significantly reduced from 13.3% at baseline to 2.8% at 12 months. S. haematobium infections and reinfections are seasonal and depend on micro-geographical settings. The risk of being infected with schistosomes in pre-school-aged children increases with increasing age. Sustained treatment of infected individuals in a community reduces prevalence over time. Participation compliance at consecutive visits and sample submission adherence are important for effective operational control interventions.

The overall pre-treatment and post-treatment prevalence of *S. haematobium* infections was 20.0% and 2.8% respectively. The results of this study revealed that the prevalence of S. haematobium in preschool children from Kwale County was high (20%) compared to that observed among school-aged in the same county (24.5%) the burden of schistosomiasis high in pre-school

At baseline, younger children had a higher prevalence but instead had a lower prevalence than older children at 2 weeks and 6 months after Praziquantel treatment. Infection intensity was significantly different by age only at baseline, where it peaked among children who were 9 years of age. The overall prevalence decreased to 6.6% at 2 weeks and 4.2% at 6 months after the

Overall, mean infection prevalence was 7.4%, Light and heavy infections were detected in82.3% and 17.7% of the positive children respectively, Prevalence of schistosomiasis decreased post-treatment with schistosomiasis

Although there was mass distribution and administration of praziquantel across Nigeria, there is yet to be a nationwide 119 assessment of the level of impact of the drug intervention on schistosomiasis prevalence. The overall prevalence of S. haematobium we recorded in the 10 states studied (10.4%). The high intensity observed suggests that transmission is still high and that the infected children reported here have been re-infected after treatment or missed treatment.

Praziquantel has an impact on reducing the prevalence and intensity of urogenital schistosomiasis. However, in the Senegal river basin, S. haematobium remains a real health problem for children living in the villages near the irrigation canals, despite regular treatment, while prevalence is not declining among those frequenting the river and the Lac de Guiers.

Before the MDA, *S haematobium* prevalence in the sentinel sites was 31.7%. *S. haematobium* was the most prevalent schistosome species in the country. Following 6annual rounds of MDA, the prevalence of *S. haematobium* decreased significantly to 0%. When comparing infection prevalence pre-treatment, the pre-MDA prevalence of 31.7% decreased to

Praziquantel administered at a single oral dose of 40 mg/kg achieved a cure rate of 91.7% and reduced the egg rate by 86.8%. The efficacy of praziquantel at 40 mg/kg is sufficient to permit continued use in treating S. mansoni-infected schoolchildren.

The MDA program only partially controlled parasite infections, owing to high rates of re-infection.

Mass drug administration for control of schistosomiasis presents low effectiveness, reinfections occur rapidly and that stand-alone anthelmintic therapy is not a sustainable choice.

The prevalence and intensity of schistosomiasis infection were significantly reduced and stopping MDA in areas with high prevalence may result in a significant rebound of infection

The prevalence of schistosomiasis was 14.5% compared to 51% in the reportable water era. The prevalence and intensity of S.

The prevalence of schistosomiasis was 14.[illegible]% compared to [illegible]1% in the reportable water era. The prevalence and intensity of S. *haematobium* were significantly reduced in this community though not yet eliminated.
