# Supplementary material for: Effects of paediatric schistosomiasis control programmes in sub-Saharan Africa: A systematic review
Source: PLoS One. 2024 May 2;19(5):e0301464. doi: 10.1371/journal.pone.0301464 (PMC11065241; doi:10.1371/journal.pone.0301464)

Search terms

schistosomiasis OR bilharziasis OR bilharzia OR katayama fever OR snail fever OR urogenital schistosomiasis OR urinary schistosomiasis OR genital schistosomiasis OR schistosoma OR haematobium OR schistosoma haematobium OR s. haematobium OR schistosoma mansoni OR s. mansoni OR mansoni OR intestinal schistosomiasis OR s. japonicum OR japonicum OR s. mekongi OR mekongi OR s. intercalatum OR intercalatum OR s. guineensis OR guineensis

AND

preventive OR chemotherapy OR control OR elimination OR wash OR sanitation OR hygiene OR education OR mass drug administration OR mda OR snail control OR molluscicides OR molluscicidal

Database Searches

1. Pubmed (20 April 2023)

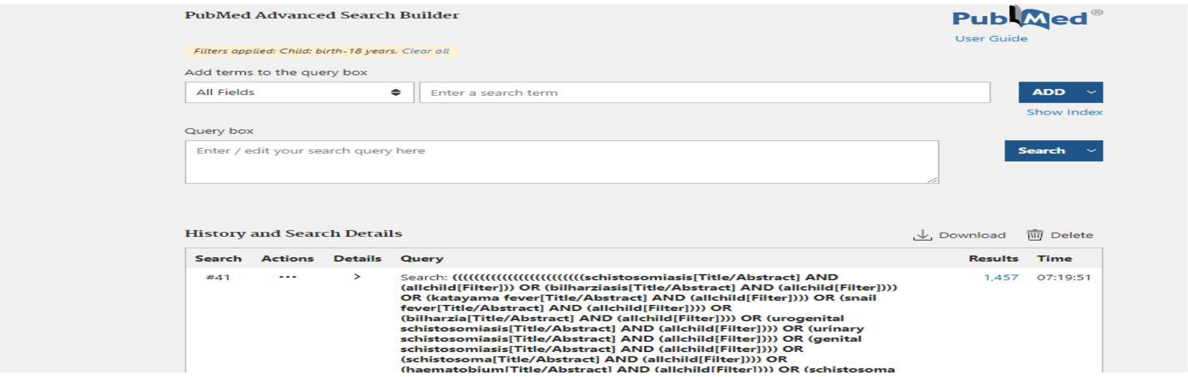

41	((((((((((((((((((((((((schistosomiasis[Title/Abstract] AND (allchild[Filter])) OR (bilharziasis[Title/Abstract] AND (allchild[Filter]))) OR (katayama fever[Title/Abstract] AND (allchild[Filter]))) OR (snail fever[Title/Abstract] AND (allchild[Filter]))) OR (bilharzia[Title/Abstract] AND (allchild[Filter]))) OR (urogenital schistosomiasis[Title/Abstract] AND (allchild[Filter]))) OR (urinary schistosomiasis[Title/Abstract] AND (allchild[Filter]))) OR (genital schistosomiasis[Title/Abstract] AND (allchild[Filter]))) OR (schistosoma[Title/Abstract] AND (allchild[Filter]))) OR (haematobium[Title/Abstract] AND (allchild[Filter]))) OR (schistosoma haematobium[Title/Abstract] AND (allchild[Filter]))) OR (s. haematobium[Title/Abstract] AND	((("schistosomiasis"[Title/Abstract] AND ("infant"[MeSH Terms] OR "child"[MeSH Terms] OR "adolescent"[MeSH Terms])) OR ("bilharziasis"[Title/Abstract] AND ("infant"[MeSH Terms] OR "child"[MeSH Terms] OR "adolescent"[MeSH Terms])) OR ("katayama fever"[Title/Abstract] AND ("infant"[MeSH Terms] OR "child"[MeSH Terms] OR "adolescent"[MeSH Terms])) OR ("snail fever"[Title/Abstract] AND ("infant"[MeSH Terms] OR "child"[MeSH Terms] OR "adolescent"[MeSH Terms])) OR ("bilharzia"[Title/Abstract] AND ("infant"[MeSH Terms]	1,457

	(allchild[Filter]))) OR (schistosoma<br>mansoni[Title/Abstract] AND<br>(allchild[Filter]))) OR (s.<br>mansoni[Title/Abstract] AND<br>(allchild[Filter]))) OR<br>(mansoni[Title/Abstract] AND<br>(allchild[Filter]))) OR (intestinal<br>schistosomiasis[Title/Abstract] AND<br>(allchild[Filter]))) OR (s.<br>japonicum[Title/Abstract] AND<br>(allchild[Filter]))) OR<br>(japonicum[Title/Abstract] AND<br>(allchild[Filter]))) OR (s.<br>mekongi[Title/Abstract] AND<br>(allchild[Filter]))) OR<br>(mekongi[Title/Abstract] AND<br>(allchild[Filter]))) OR (s.<br>intercalatum[Title/Abstract] AND<br>(allchild[Filter]))) OR<br>(intercalatum[Title/Abstract] AND<br>(allchild[Filter]))) OR (s.<br>guineensis[Title/Abstract] AND<br>(allchild[Filter]))) OR<br>(guineensis[Title/Abstract] AND<br>(allchild[Filter])) AND (allchild[Filter]))<br>AND ((((((((((((preventive[Title/Abstract]<br>AND (allchild[Filter])) OR<br>(chemotherapy[Title/Abstract] AND<br>(allchild[Filter]))) OR<br>(control[Title/Abstract] AND<br>(allchild[Filter]))) OR<br>(elimination[Title/Abstract] AND<br>(allchild[Filter]))) OR<br>(wash[Title/Abstract] AND<br>(allchild[Filter]))) OR<br>(sanitation[Title/Abstract] AND<br>(allchild[Filter]))) OR<br>(hygiene[Title/Abstract] AND<br>(allchild[Filter]))) OR<br>(education[Title/Abstract] AND<br>(allchild[Filter]))) OR (mass drug<br>administration[Title/Abstract] AND<br>(allchild[Filter]))) OR (mda[Title/Abstract]<br>AND (allchild[Filter]))) OR (snail<br>control[Title/Abstract] AND<br>(allchild[Filter]))) OR<br>(molluscicides[Title/Abstract] AND<br>(allchild[Filter]))) OR<br>(molluscicidal[Title/Abstract] AND<br>(allchild[Filter])) AND (allchild[Filter]))	OR "child"[MeSH Terms] OR<br>"adolescent"[MeSH Terms]))<br>OR ("urogenital<br>schistosomiasis"[Title/Abstrac<br>t] AND ("infant"[MeSH<br>Terms] OR "child"[MeSH<br>Terms] OR<br>"adolescent"[MeSH Terms]))<br>OR ("urinary<br>schistosomiasis"[Title/Abstrac<br>t] AND ("infant"[MeSH<br>Terms] OR "child"[MeSH<br>Terms] OR<br>"adolescent"[MeSH Terms]))<br>OR ("genital<br>schistosomiasis"[Title/Abstrac<br>t] AND ("infant"[MeSH<br>Terms] OR "child"[MeSH<br>Terms] OR<br>"adolescent"[MeSH Terms]))<br>OR<br>("schistosoma"[Title/Abstract<br>] AND ("infant"[MeSH<br>Terms] OR "child"[MeSH<br>Terms] OR<br>"adolescent"[MeSH Terms]))<br>OR<br>("haematobium"[Title/Abstrac<br>t] AND ("infant"[MeSH<br>Terms] OR "child"[MeSH<br>Terms] OR<br>"adolescent"[MeSH Terms]))<br>OR ("schistosoma<br>haematobium"[Title/Abstract]<br>AND ("infant"[MeSH Terms]<br>OR "child"[MeSH Terms] OR<br>"adolescent"[MeSH Terms]))<br>OR ("s<br>haematobium"[Title/Abstract]<br>AND ("infant"[MeSH Terms]<br>OR "child"[MeSH Terms] OR<br>"adolescent"[MeSH Terms]))<br>OR ("schistosoma<br>mansoni"[Title/Abstract]<br>AND ("infant"[MeSH Terms]<br>OR "child"[MeSH Terms] OR<br>"adolescent"[MeSH Terms]))<br>OR ("s<br>mansoni"[Title/Abstract]<br>AND ("infant"[MeSH Terms]	

		OR "child"[MeSH Terms] OR "adolescent"[MeSH Terms])) OR ("mansoni"[Title/Abstract] AND ("infant"[MeSH Terms] OR "child"[MeSH Terms] OR "adolescent"[MeSH Terms])) OR ("intestinal schistosomiasis"[Title/Abstract] AND ("infant"[MeSH Terms] OR "child"[MeSH Terms] OR "adolescent"[MeSH Terms])) OR ("s japonicum"[Title/Abstract] AND ("infant"[MeSH Terms] OR "child"[MeSH Terms] OR "adolescent"[MeSH Terms])) OR ("japonicum"[Title/Abstract] AND ("infant"[MeSH Terms] OR "child"[MeSH Terms] OR "adolescent"[MeSH Terms])) OR ("s mekongi"[Title/Abstract] AND ("infant"[MeSH Terms] OR "child"[MeSH Terms] OR "adolescent"[MeSH Terms])) OR ("mekongi"[Title/Abstract] AND ("infant"[MeSH Terms] OR "child"[MeSH Terms] OR "adolescent"[MeSH Terms])) OR ("s intercalatum"[Title/Abstract] AND ("infant"[MeSH Terms] OR "child"[MeSH Terms] OR "adolescent"[MeSH Terms])) OR ("intercalatum"[Title/Abstract] AND ("infant"[MeSH Terms] OR "child"[MeSH Terms] OR "adolescent"[MeSH Terms])) OR ("s guineensis"[Title/Abstract] AND ("infant"[MeSH Terms] OR "child"[MeSH Terms] OR "adolescent"[MeSH Terms])) OR	

		("guineensis"[Title/Abstract] AND ("infant"[MeSH Terms] OR "child"[MeSH Terms] OR "adolescent"[MeSH Terms]))) AND ("infant"[MeSH Terms] OR "child"[MeSH Terms] OR "adolescent"[MeSH Terms]) AND ((("preventive"[Title/Abstract] AND ("infant"[MeSH Terms] OR "child"[MeSH Terms] OR "adolescent"[MeSH Terms])) OR ("chemotherapy"[Title/Abstract] AND ("infant"[MeSH Terms] OR "child"[MeSH Terms] OR "adolescent"[MeSH Terms])) OR ("control"[Title/Abstract] AND ("infant"[MeSH Terms] OR "child"[MeSH Terms] OR "adolescent"[MeSH Terms])) OR ("elimination"[Title/Abstract] AND ("infant"[MeSH Terms] OR "child"[MeSH Terms] OR "adolescent"[MeSH Terms])) OR ("wash"[Title/Abstract] AND ("infant"[MeSH Terms] OR "child"[MeSH Terms] OR "adolescent"[MeSH Terms])) OR ("sanitation"[Title/Abstract] AND ("infant"[MeSH Terms] OR "child"[MeSH Terms] OR "adolescent"[MeSH Terms])) OR ("hygiene"[Title/Abstract] AND ("infant"[MeSH Terms] OR "child"[MeSH Terms] OR "adolescent"[MeSH Terms])) OR ("education"[Title/Abstract] AND ("infant"[MeSH Terms] OR "child"[MeSH Terms] OR "adolescent"[MeSH Terms])) OR ("mass drug administration"[Title/Abstract] AND ("infant"[MeSH Terms] OR "child"[MeSH Terms]	

		Terms] OR "adolescent"[MeSH Terms])) OR ("mda"[Title/Abstract] AND ("infant"[MeSH Terms] OR "child"[MeSH Terms] OR "adolescent"[MeSH Terms])) OR ("snail control"[Title/Abstract] AND ("infant"[MeSH Terms] OR "child"[MeSH Terms] OR "adolescent"[MeSH Terms])) OR ("molluscicides"[Title/Abstract] AND ("infant"[MeSH Terms] OR "child"[MeSH Terms] OR "adolescent"[MeSH Terms])) OR ("molluscicidal"[Title/Abstract] AND ("infant"[MeSH Terms] OR "child"[MeSH Terms] OR "adolescent"[MeSH Terms]))) AND ("infant"[MeSH Terms] OR "child"[MeSH Terms] OR "adolescent"[MeSH Terms]))) AND ((allchild[Filter]) AND (2000:2023[pdat]))	
40	((((((((((((((((((((((schistosomiasis[Title/Abstract] AND (allchild[Filter])) OR (bilharziasis[Title/Abstract] AND (allchild[Filter]))) OR (katayama fever[Title/Abstract] AND (allchild[Filter]))) OR (snail fever[Title/Abstract] AND (allchild[Filter]))) OR (bilharzia[Title/Abstract] AND (allchild[Filter]))) OR (urogenital schistosomiasis[Title/Abstract] AND (allchild[Filter]))) OR (urinary schistosomiasis[Title/Abstract] AND (allchild[Filter]))) OR (genital schistosomiasis[Title/Abstract] AND (allchild[Filter]))) OR (schistosoma[Title/Abstract] AND (allchild[Filter]))) OR (haematobium[Title/Abstract] AND (allchild[Filter]))) OR (schistosoma haematobium[Title/Abstract] AND	((("schistosomiasis"[Title/Abstract] AND ("infant"[MeSH Terms] OR "child"[MeSH Terms] OR "adolescent"[MeSH Terms])) OR ("bilharziasis"[Title/Abstract] AND ("infant"[MeSH Terms] OR "child"[MeSH Terms] OR "adolescent"[MeSH Terms])) OR ("katayama fever"[Title/Abstract] AND ("infant"[MeSH Terms] OR "child"[MeSH Terms] OR "adolescent"[MeSH Terms])) OR ("snail fever"[Title/Abstract] AND ("infant"[MeSH Terms] OR "child"[MeSH Terms] OR "adolescent"[MeSH Terms])) OR	2,178

	(allchild[Filter]))) OR (s. haematobium[Title/Abstract] AND (allchild[Filter]))) OR (schistosoma mansoni[Title/Abstract] AND (allchild[Filter]))) OR (s. mansoni[Title/Abstract] AND (allchild[Filter]))) OR (mansoni[Title/Abstract] AND (allchild[Filter]))) OR (intestinal schistosomiasis[Title/Abstract] AND (allchild[Filter]))) OR (s. japonicum[Title/Abstract] AND (allchild[Filter]))) OR (japonicum[Title/Abstract] AND (allchild[Filter]))) OR (s. mekongi[Title/Abstract] AND (allchild[Filter]))) OR (mekongi[Title/Abstract] AND (allchild[Filter]))) OR (s. intercalatum[Title/Abstract] AND (allchild[Filter]))) OR (intercalatum[Title/Abstract] AND (allchild[Filter]))) OR (s. guineensis[Title/Abstract] AND (allchild[Filter]))) OR (guineensis[Title/Abstract] AND (allchild[Filter])) AND (allchild[Filter])) AND ((((((((((((preventive[Title/Abstract] AND (allchild[Filter])) OR (chemotherapy[Title/Abstract] AND (allchild[Filter]))) OR (control[Title/Abstract] AND (allchild[Filter]))) OR (elimination[Title/Abstract] AND (allchild[Filter]))) OR (wash[Title/Abstract] AND (allchild[Filter]))) OR (sanitation[Title/Abstract] AND (allchild[Filter]))) OR (hygiene[Title/Abstract] AND (allchild[Filter]))) OR (education[Title/Abstract] AND (allchild[Filter]))) OR (mass drug administration[Title/Abstract] AND (allchild[Filter]))) OR (mda[Title/Abstract] AND (allchild[Filter]))) OR (snail control[Title/Abstract] AND (allchild[Filter]))) OR (molluscicides[Title/Abstract] AND (allchild[Filter]))) OR	("bilharzia"[Title/Abstract] AND ("infant"[MeSH Terms] OR "child"[MeSH Terms] OR "adolescent"[MeSH Terms])) OR ("urogenital schistosomiasis"[Title/Abstract] AND ("infant"[MeSH Terms] OR "child"[MeSH Terms] OR "adolescent"[MeSH Terms])) OR ("urinary schistosomiasis"[Title/Abstract] AND ("infant"[MeSH Terms] OR "child"[MeSH Terms] OR "adolescent"[MeSH Terms])) OR ("genital schistosomiasis"[Title/Abstract] AND ("infant"[MeSH Terms] OR "child"[MeSH Terms] OR "adolescent"[MeSH Terms])) OR ("schistosoma"[Title/Abstract] AND ("infant"[MeSH Terms] OR "child"[MeSH Terms] OR "adolescent"[MeSH Terms])) OR ("haematobium"[Title/Abstract] AND ("infant"[MeSH Terms] OR "child"[MeSH Terms] OR "adolescent"[MeSH Terms])) OR ("schistosoma haematobium"[Title/Abstract] AND ("infant"[MeSH Terms] OR "child"[MeSH Terms] OR "adolescent"[MeSH Terms])) OR ("s haematobium"[Title/Abstract] AND ("infant"[MeSH Terms] OR "child"[MeSH Terms] OR "adolescent"[MeSH Terms])) OR ("schistosoma mansoni"[Title/Abstract] AND ("infant"[MeSH Terms] OR "child"[MeSH Terms] OR "adolescent"[MeSH Terms])) OR ("s	

	(molluscicidal[Title/Abstract] AND (allchild[Filter])) AND (allchild[Filter]))	mansoni"[Title/Abstract] AND ("infant"[MeSH Terms] OR "child"[MeSH Terms] OR "adolescent"[MeSH Terms])) OR ("mansoni"[Title/Abstract] AND ("infant"[MeSH Terms] OR "child"[MeSH Terms] OR "adolescent"[MeSH Terms])) OR ("intestinal schistosomiasis"[Title/Abstract] AND ("infant"[MeSH Terms] OR "child"[MeSH Terms] OR "adolescent"[MeSH Terms])) OR ("s japonicum"[Title/Abstract] AND ("infant"[MeSH Terms] OR "child"[MeSH Terms] OR "adolescent"[MeSH Terms])) OR ("japonicum"[Title/Abstract] AND ("infant"[MeSH Terms] OR "child"[MeSH Terms] OR "adolescent"[MeSH Terms])) OR ("s mekongi"[Title/Abstract] AND ("infant"[MeSH Terms] OR "child"[MeSH Terms] OR "adolescent"[MeSH Terms])) OR ("mekongi"[Title/Abstract] AND ("infant"[MeSH Terms] OR "child"[MeSH Terms] OR "adolescent"[MeSH Terms])) OR ("s intercalatum"[Title/Abstract] AND ("infant"[MeSH Terms] OR "child"[MeSH Terms] OR "adolescent"[MeSH Terms])) OR ("intercalatum"[Title/Abstract] AND ("infant"[MeSH Terms] OR "child"[MeSH Terms] OR "adolescent"[MeSH Terms])) OR ("s guineensis"[Title/Abstract] AND ("infant"[MeSH Terms] OR "child"[MeSH Terms] OR	

		"adolescent"[MeSH Terms])) OR ("guineensis"[Title/Abstract] AND ("infant"[MeSH Terms] OR "child"[MeSH Terms] OR "adolescent"[MeSH Terms]))) AND ("infant"[MeSH Terms] OR "child"[MeSH Terms] OR "adolescent"[MeSH Terms]) AND ((("preventive"[Title/Abstract] AND ("infant"[MeSH Terms] OR "child"[MeSH Terms] OR "adolescent"[MeSH Terms])) OR ("chemotherapy"[Title/Abstract] AND ("infant"[MeSH Terms] OR "child"[MeSH Terms] OR "adolescent"[MeSH Terms])) OR ("control"[Title/Abstract] AND ("infant"[MeSH Terms] OR "child"[MeSH Terms] OR "adolescent"[MeSH Terms])) OR ("elimination"[Title/Abstract] AND ("infant"[MeSH Terms] OR "child"[MeSH Terms] OR "adolescent"[MeSH Terms])) OR ("wash"[Title/Abstract] AND ("infant"[MeSH Terms] OR "child"[MeSH Terms] OR "adolescent"[MeSH Terms])) OR ("sanitation"[Title/Abstract] AND ("infant"[MeSH Terms] OR "child"[MeSH Terms] OR "adolescent"[MeSH Terms])) OR ("hygiene"[Title/Abstract] AND ("infant"[MeSH Terms] OR "child"[MeSH Terms] OR "adolescent"[MeSH Terms])) OR ("education"[Title/Abstract] AND ("infant"[MeSH Terms] OR "child"[MeSH Terms] OR "adolescent"[MeSH Terms])) OR ("mass drug administration"[Title/Abstract	

		] AND ("infant"[MeSH Terms] OR "child"[MeSH Terms] OR "adolescent"[MeSH Terms])) OR ("mda"[Title/Abstract] AND ("infant"[MeSH Terms] OR "child"[MeSH Terms] OR "adolescent"[MeSH Terms])) OR ("snail control"[Title/Abstract] AND ("infant"[MeSH Terms] OR "child"[MeSH Terms] OR "adolescent"[MeSH Terms])) OR ("molluscicides"[Title/Abstract] AND ("infant"[MeSH Terms] OR "child"[MeSH Terms] OR "adolescent"[MeSH Terms])) OR ("molluscicidal"[Title/Abstract] AND ("infant"[MeSH Terms] OR "child"[MeSH Terms] OR "adolescent"[MeSH Terms]))) AND ("infant"[MeSH Terms] OR "child"[MeSH Terms] OR "adolescent"[MeSH Terms]))) AND (allchild[Filter])	
39	((((((((((((preventive[Title/Abstract] AND (allchild[Filter])) OR (chemotherapy[Title/Abstract] AND (allchild[Filter]))) OR (control[Title/Abstract] AND (allchild[Filter]))) OR (elimination[Title/Abstract] AND (allchild[Filter]))) OR (wash[Title/Abstract] AND (allchild[Filter]))) OR (sanitation[Title/Abstract] AND (allchild[Filter]))) OR (hygiene[Title/Abstract] AND (allchild[Filter]))) OR (education[Title/Abstract] AND (allchild[Filter]))) OR (mass drug administration[Title/Abstract] AND (allchild[Filter]))) OR (mda[Title/Abstract] AND (allchild[Filter]))) OR (snail control[Title/Abstract] AND	(("preventive"[Title/Abstract] AND ("infant"[MeSH Terms] OR "child"[MeSH Terms] OR "adolescent"[MeSH Terms])) OR ("chemotherapy"[Title/Abstract] AND ("infant"[MeSH Terms] OR "child"[MeSH Terms] OR "adolescent"[MeSH Terms])) OR ("control"[Title/Abstract] AND ("infant"[MeSH Terms] OR "child"[MeSH Terms] OR "adolescent"[MeSH Terms])) OR ("elimination"[Title/Abstract] AND ("infant"[MeSH Terms] OR "child"[MeSH Terms] OR "adolescent"[MeSH Terms])) OR ("wash"[Title/Abstract]	548,478

	(allchild[Filter]))) OR (molluscicides[Title/Abstract] AND (allchild[Filter]))) OR (molluscicidal[Title/Abstract] AND (allchild[Filter]))	AND ("infant"[MeSH Terms] OR "child"[MeSH Terms] OR "adolescent"[MeSH Terms])) OR ("sanitation"[Title/Abstract] AND ("infant"[MeSH Terms] OR "child"[MeSH Terms] OR "adolescent"[MeSH Terms])) OR ("hygiene"[Title/Abstract] AND ("infant"[MeSH Terms] OR "child"[MeSH Terms] OR "adolescent"[MeSH Terms])) OR ("education"[Title/Abstract] AND ("infant"[MeSH Terms] OR "child"[MeSH Terms] OR "adolescent"[MeSH Terms])) OR ("mass drug administration"[Title/Abstract] AND ("infant"[MeSH Terms] OR "child"[MeSH Terms] OR "adolescent"[MeSH Terms])) OR ("mda"[Title/Abstract] AND ("infant"[MeSH Terms] OR "child"[MeSH Terms] OR "adolescent"[MeSH Terms])) OR ("snail control"[Title/Abstract] AND ("infant"[MeSH Terms] OR "child"[MeSH Terms] OR "adolescent"[MeSH Terms])) OR ("molluscicides"[Title/Abstract] AND ("infant"[MeSH Terms] OR "child"[MeSH Terms] OR "adolescent"[MeSH Terms])) OR ("molluscicidal"[Title/Abstract] AND ("infant"[MeSH Terms] OR "child"[MeSH Terms] OR "adolescent"[MeSH Terms]))) AND (allchild[Filter])	
38	((((((((((((((((((((schistosomiasis[Title/Abstract] AND (allchild[Filter])) OR (bilharziasis[Title/Abstract] AND (allchild[Filter]))) OR (katayama	(("schistosomiasis"[Title/Abstract] AND ("infant"[MeSH Terms] OR "child"[MeSH Terms] OR	6,156

	fever[Title/Abstract] AND (allchild[Filter]))) OR (snail fever[Title/Abstract] AND (allchild[Filter]))) OR (bilharzia[Title/Abstract] AND (allchild[Filter]))) OR (urogenital schistosomiasis[Title/Abstract] AND (allchild[Filter]))) OR (urinary schistosomiasis[Title/Abstract] AND (allchild[Filter]))) OR (genital schistosomiasis[Title/Abstract] AND (allchild[Filter]))) OR (schistosoma[Title/Abstract] AND (allchild[Filter]))) OR (haematobium[Title/Abstract] AND (allchild[Filter]))) OR (schistosoma haematobium[Title/Abstract] AND (allchild[Filter]))) OR (s. haematobium[Title/Abstract] AND (allchild[Filter]))) OR (schistosoma mansoni[Title/Abstract] AND (allchild[Filter]))) OR (s. mansoni[Title/Abstract] AND (allchild[Filter]))) OR (mansoni[Title/Abstract] AND (allchild[Filter]))) OR (intestinal schistosomiasis[Title/Abstract] AND (allchild[Filter]))) OR (s. japonicum[Title/Abstract] AND (allchild[Filter]))) OR (japonicum[Title/Abstract] AND (allchild[Filter]))) OR (s. mekongi[Title/Abstract] AND (allchild[Filter]))) OR (mekongi[Title/Abstract] AND (allchild[Filter]))) OR (s. intercalatum[Title/Abstract] AND (allchild[Filter]))) OR (intercalatum[Title/Abstract] AND (allchild[Filter]))) OR (s. guineensis[Title/Abstract] AND (allchild[Filter]))) OR (guineensis[Title/Abstract] AND (allchild[Filter]))	"adolescent"[MeSH Terms])) OR ("bilharziasis"[Title/Abstract] AND ("infant"[MeSH Terms] OR "child"[MeSH Terms] OR "adolescent"[MeSH Terms])) OR ("katayama fever"[Title/Abstract] AND ("infant"[MeSH Terms] OR "child"[MeSH Terms] OR "adolescent"[MeSH Terms])) OR ("snail fever"[Title/Abstract] AND ("infant"[MeSH Terms] OR "child"[MeSH Terms] OR "adolescent"[MeSH Terms])) OR ("bilharzia"[Title/Abstract] AND ("infant"[MeSH Terms] OR "child"[MeSH Terms] OR "adolescent"[MeSH Terms])) OR ("urogenital schistosomiasis"[Title/Abstract] AND ("infant"[MeSH Terms] OR "child"[MeSH Terms] OR "adolescent"[MeSH Terms])) OR ("urinary schistosomiasis"[Title/Abstract] AND ("infant"[MeSH Terms] OR "child"[MeSH Terms] OR "adolescent"[MeSH Terms])) OR ("genital schistosomiasis"[Title/Abstract] AND ("infant"[MeSH Terms] OR "child"[MeSH Terms] OR "adolescent"[MeSH Terms])) OR ("schistosoma"[Title/Abstract] AND ("infant"[MeSH Terms] OR "child"[MeSH Terms] OR "adolescent"[MeSH Terms])) OR ("haematobium"[Title/Abstract] AND ("infant"[MeSH Terms] OR "child"[MeSH Terms] OR	

		"adolescent"[MeSH Terms])) OR ("schistosoma haematobium"[Title/Abstract] AND ("infant"[MeSH Terms] OR "child"[MeSH Terms] OR "adolescent"[MeSH Terms])) OR ("s haematobium"[Title/Abstract] AND ("infant"[MeSH Terms] OR "child"[MeSH Terms] OR "adolescent"[MeSH Terms])) OR ("schistosoma mansoni"[Title/Abstract] AND ("infant"[MeSH Terms] OR "child"[MeSH Terms] OR "adolescent"[MeSH Terms])) OR ("s mansoni"[Title/Abstract] AND ("infant"[MeSH Terms] OR "child"[MeSH Terms] OR "adolescent"[MeSH Terms])) OR ("mansoni"[Title/Abstract] AND ("infant"[MeSH Terms] OR "child"[MeSH Terms] OR "adolescent"[MeSH Terms])) OR ("intestinal schistosomiasis"[Title/Abstract] AND ("infant"[MeSH Terms] OR "child"[MeSH Terms] OR "adolescent"[MeSH Terms])) OR ("s japonicum"[Title/Abstract] AND ("infant"[MeSH Terms] OR "child"[MeSH Terms] OR "adolescent"[MeSH Terms])) OR ("japonicum"[Title/Abstract] AND ("infant"[MeSH Terms] OR "child"[MeSH Terms] OR "adolescent"[MeSH Terms])) OR ("s mekongi"[Title/Abstract] AND ("infant"[MeSH Terms] OR "child"[MeSH Terms] OR "adolescent"[MeSH Terms])) OR ("mekongi"[Title/Abstract] AND ("infant"[MeSH Terms]	

		OR "child"[MeSH Terms] OR "adolescent"[MeSH Terms])) OR ("s intercalatum"[Title/Abstract] AND ("infant"[MeSH Terms] OR "child"[MeSH Terms] OR "adolescent"[MeSH Terms])) OR ("intercalatum"[Title/Abstract] AND ("infant"[MeSH Terms] OR "child"[MeSH Terms] OR "adolescent"[MeSH Terms])) OR ("s guineensis"[Title/Abstract] AND ("infant"[MeSH Terms] OR "child"[MeSH Terms] OR "adolescent"[MeSH Terms])) OR ("guineensis"[Title/Abstract] AND ("infant"[MeSH Terms] OR "child"[MeSH Terms] OR "adolescent"[MeSH Terms]))) AND (allchild[Filter])	
37	molluscicidal[Title/Abstract]	("molluscicidal"[Title/Abstract]) AND (allchild[Filter])	4
36	molluscicides[Title/Abstract]	("molluscicides"[Title/Abstract]) AND (allchild[Filter])	16
35	snail control[Title/Abstract]	("snail control"[Title/Abstract]) AND (allchild[Filter])	79
34	mda[Title/Abstract]	("mda"[Title/Abstract]) AND (allchild[Filter])	2,151
33	mass drug administration[Title/Abstract]	("mass drug administration"[Title/Abstract]) AND (allchild[Filter])	910
32	education[Title/Abstract]	("education"[Title/Abstract]) AND (allchild[Filter])	124,373
31	hygiene[Title/Abstract]	("hygiene"[Title/Abstract]) AND (allchild[Filter])	14,560
30	sanitation[Title/Abstract]	("sanitation"[Title/Abstract]) AND (allchild[Filter])	3,556

29	wash[Title/Abstract]	("wash"[Title/Abstract]) AND (allchild[Filter])	2,421
28	elimination[Title/Abstract]	("elimination"[Title/Abstract]) AND (allchild[Filter])	16,537
27	control[Title/Abstract]	("control"[Title/Abstract]) AND (allchild[Filter])	343,241
26	chemotherapy[Title/Abstract]	("chemotherapy"[Title/Abstract]) AND (allchild[Filter])	53,426
25	preventive[Title/Abstract]	("preventive"[Title/Abstract]) AND (allchild[Filter])	35,917
24	guineensis[Title/Abstract]	("guineensis"[Title/Abstract]) AND (allchild[Filter])	10
23	s. guineensis[Title/Abstract]	("s guineensis"[Title/Abstract]) AND (allchild[Filter])	2
22	intercalatum[Title/Abstract]	("intercalatum"[Title/Abstract]) AND (allchild[Filter])	69
21	s. intercalatum[Title/Abstract]	("s intercalatum"[Title/Abstract]) AND (allchild[Filter])	43
20	mekongi[Title/Abstract]	("mekongi"[Title/Abstract]) AND (allchild[Filter])	42
19	s. mekongi[Title/Abstract]	("s mekongi"[Title/Abstract]) AND (allchild[Filter])	27
18	japonicum[Title/Abstract]	("japonicum"[Title/Abstract]) AND (allchild[Filter])	358
17	s. japonicum[Title/Abstract]	("s japonicum"[Title/Abstract]) AND (allchild[Filter])	189
16	intestinal schistosomiasis[Title/Abstract]	("intestinal schistosomiasis"[Title/Abstract]) AND (allchild[Filter])	259
15	mansoni[Title/Abstract]	("mansoni"[Title/Abstract]) AND (allchild[Filter])	2,550
14	s. mansoni[Title/Abstract]	("s mansoni"[Title/Abstract]) AND (allchild[Filter])	1,285

13	schistosoma mansoni[Title/Abstract]	("schistosoma mansoni"[Title/Abstract]) AND (allchild[Filter])	1,795
12	s. haematobium[Title/Abstract]	("s haematobium"[Title/Abstract]) AND (allchild[Filter])	983
11	schistosoma haematobium[Title/Abstract]	("schistosoma haematobium"[Title/Abstract]) AND (allchild[Filter])	1,213
10	haematobium[Title/Abstract]	("haematobium"[Title/Abstract]) AND (allchild[Filter])	1,574
9	schistosoma[Title/Abstract]	("schistosoma"[Title/Abstract]) AND (allchild[Filter])	3,378
8	genital schistosomiasis[Title/Abstract]	("genital schistosomiasis"[Title/Abstract]) AND (allchild[Filter])	68
7	urinary schistosomiasis[Title/Abstract]	("urinary schistosomiasis"[Title/Abstract]) AND (allchild[Filter])	522
6	urogenital schistosomiasis[Title/Abstract]	("urogenital schistosomiasis"[Title/Abstract]) AND (allchild[Filter])	153
5	bilharzia[Title/Abstract]	("bilharzia"[Title/Abstract]) AND (allchild[Filter])	91
4	snail fever[Title/Abstract]	("snail fever"[Title/Abstract]) AND (allchild[Filter])	0
3	katayama fever[Title/Abstract]	("katayama fever"[Title/Abstract]) AND (allchild[Filter])	4
2	bilharziasis[Title/Abstract]	("bilharziasis"[Title/Abstract]) AND (allchild[Filter])	378
1	schistosomiasis[Title/Abstract]	("schistosomiasis"[Title/Abstract]) AND (allchild[Filter])	4,511

Embase (23 April 2023)

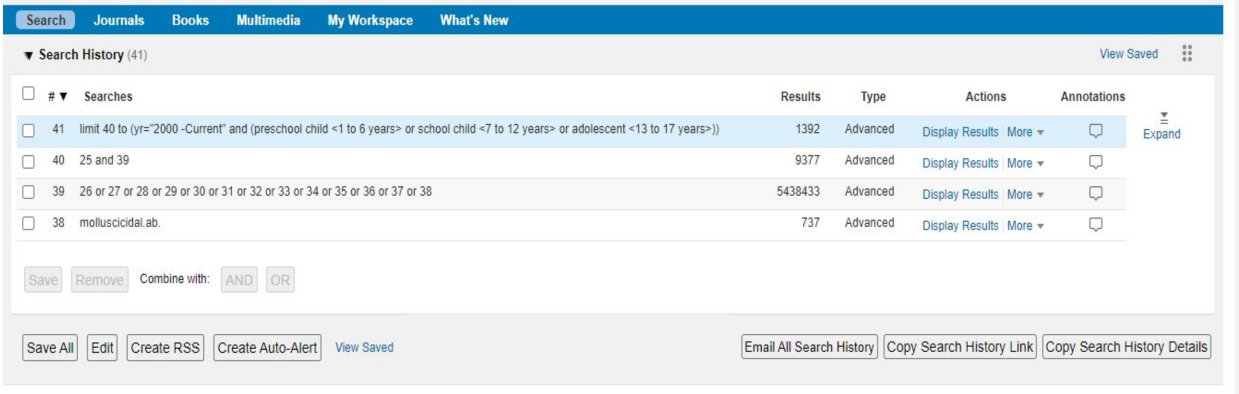

Search | Journals | Books | Multimedia | My Workspace | What's New

Search History (41)

#	Searches	Results	Type	Actions
41	limit 40 to (yr="2000 -Current" and (preschool child <1 to 6 years> or school child <7 to 12 years> or adolescent <13 to 17 years>))	1392	Advanced	Display Results More
40	25 and 39	9377	Advanced	Display Results More
39	26 or 27 or 28 or 29 or 30 or 31 or 32 or 33 or 34 or 35 or 36 or 37 or 38	5438433	Advanced	Display Results More
38	molluscicidal.ab.	737	Advanced	Display Results More

#	Query	Results from 25 Apr 2023
1	schistosomiasis.ab.	15,709
2	bilharzia.ab.	287
3	bilharziasis.ab.	459
4	katayama fever.ab.	39
5	snail fever.ab.	18
6	urogenital schistosomiasis.ab.	395
7	urinary schistosomiasis.ab.	766
8	genital schistosomiasis.ab.	193
9	schistosoma.ab.	17,183
10	haematobium.ab.	3,556
11	schistosoma haematobium.ab.	2,355
12	s haematobium.ab.	2,297
13	schistosoma mansoni.ab.	10,110
14	s mansoni.ab.	6,535
15	mansoni.ab.	12,796
16	intestinal schistosomiasis.ab.	619

17	s japonicum.ab.	2,344
18	japonicum.ab.	6,545
19	s mekongi.ab.	135
20	mekongi.ab.	187
21	s intercalatum.ab.	136
22	intercalatum.ab.	190
23	s guineensis.ab.	12
24	guineensis.ab.	590
25	1 or 2 or 3 or 4 or 5 or 6 or 7 or 8 or 9 or 10 or 11 or 12 or 13 or 14 or 15 or 16 or 17 or 18 or 19 or 20 or 21 or 22 or 23 or 24	29,694
26	preventive.ab.	193,700
27	chemotherapy.ab.	653,330
28	control.ab.	3,849,327
29	elimination.ab.	187,679
30	wash.ab.	29,832
31	sanitation.ab.	14,134
32	hygiene.ab.	72,497
33	education.ab.	668,836
34	mass drug aministration.ab.	1
35	mda.ab.	105,595
36	snail control.ab.	462
37	molluscicides.ab.	343
38	molluscicidal.ab.	737
39	26 or 27 or 28 or 29 or 30 or 31 or 32 or 33 or 34 or 35 or 36 or 37 or 38	5,438,433
40	25 and 39	9,377
41	limit 40 to (yr="2000 -Current" and (preschool child <1 to 6 years> or school child <7 to 12 years> or adolescent <13 to 17 years>))	1,392

MEDLINE Complete (26 April 2023)

EBSCOhost

Tuesday, April 25, 2023 9:59:25 PM

#	Query	Limiters/Expanders	Last Run Via	Results
S38	((S1 OR S2 OR S3 OR S4 OR S5 OR S6 OR S7 OR S8 OR S9 OR S10 OR S11 OR S12 OR S13 OR S14 OR S15 OR S16 OR S17 OR S18 OR S19 OR S20 OR S21 OR S22 OR S23 OR S24) AND (S25 OR S26 OR S27 OR S28 OR S29 OR S30 OR S31 OR S32 OR S33 OR S34 OR S35)) AND (S36 AND S37)	Search modes - Find all my search terms	Interface - EBSCOhost Research Databases<br>Search Screen - Advanced Search<br>Database - MEDLINE Complete	1,456
S37	(S1 OR S2 OR S3 OR S4 OR S5 OR S6 OR S7 OR S8 OR S9 OR S10 OR S11 OR S12 OR S13 OR S14 OR S15 OR S16 OR S17 OR S18 OR S19 OR S20 OR S21 OR S22 OR S23 OR S24) AND (S25 OR S26 OR S27 OR S28 OR S29 OR S30 OR S31 OR S32 OR S33 OR S34 OR S35)	Search modes - Find all my search terms	Interface - EBSCOhost Research Databases<br>Search Screen - Advanced Search<br>Database - MEDLINE Complete	1,456

#	Query	Limiters/Expanders	Last Run Via	Results
S38	((S1 OR S2 OR S3 OR S4 OR S5 OR S6 OR S7 OR S8 OR S9 OR S10 OR S11 OR S12 OR S13 OR S14 OR S15 OR S16 OR S17 OR S18 OR S19 OR S20 OR S21 OR S22 OR S23 OR S24) AND (S25 OR S26 OR S27 OR S28 OR S29 OR S30 OR S31 OR S32 OR S33 OR S34 OR S35)) AND (S36 AND S37)	Search modes - Find all my search terms	Interface - EBSCOhost Research Databases<br>Search Screen - Advanced Search<br>Database - MEDLINE Complete	1,456
S37	(S1 OR S2 OR S3 OR S4 OR S5 OR S6 OR S7 OR S8 OR S9 OR S10 OR S11 OR S12 OR S13 OR S14 OR S15 OR S16 OR S17 OR S18 OR S19 OR S20 OR S21 OR S22 OR S23 OR S24) AND (S25 OR S26 OR S27 OR S28 OR S29 OR S30 OR S31 OR S32 OR S33 OR S34 OR S35)	Search modes - Find all my search terms	Interface - EBSCOhost Research Databases<br>Search Screen - Advanced Search<br>Database - MEDLINE Complete	1,456

S36	S1 OR S2 OR S3 OR S4 OR S5 OR S6 OR S7 OR S8 OR S9 OR S10 OR S11 OR S12 OR S13 OR S14 OR S15 OR S16 OR S17 OR S18 OR S19 OR S20 OR S21 OR S22 OR S23 OR S24	Search modes - Find all my search terms	Interface - EBSCOhost Research Databases<br>Search Screen - Advanced Search<br>Database - MEDLINE Complete	2,793
S35	AB molluscicides	Limiters - Date of Publication: 20000101-20230531; Age Related: All Child: 0-18 years<br>Search modes - Find all my search terms	Interface - EBSCOhost Research Databases<br>Search Screen - Advanced Search<br>Database - MEDLINE Complete	15
S34	AB snail control	Limiters - Date of Publication: 20000101-20230531; Age Related: All Child: 0-18 years<br>Search modes - Find all my search terms	Interface - EBSCOhost Research Databases<br>Search Screen - Advanced Search<br>Database - MEDLINE Complete	200
S33	AB mda	Limiters - Date of Publication: 20000101-20230531; Age Related: All Child: 0-18 years<br>Search modes - Find all my search terms	Interface - EBSCOhost Research Databases<br>Search Screen - Advanced Search<br>Database - MEDLINE Complete	1,898
S32	AB mass drug administration	Limiters - Date of Publication: 20000101-20230531; Age Related: All Child: 0-18 years<br>Search modes - Find all my search terms	Interface - EBSCOhost Research Databases<br>Search Screen - Advanced Search<br>Database - MEDLINE Complete	1,366
S31	AB education	Limiters - Date of Publication: 20000101-20230531; Age Related: All Child: 0-18 years<br>Search modes - Find all my search terms	Interface - EBSCOhost Research Databases<br>Search Screen - Advanced Search<br>Database - MEDLINE Complete	94,083
S30	AB hygiene	Limiters - Date of Publication: 20000101-20230531; Age Related: All Child: 0-18 years	Interface - EBSCOhost Research Databases<br>Search Screen - Advanced Search	10,023

		Search modes - Find all my search terms	Database - MEDLINE Complete	
S29	AB wash	Limiters - Date of Publication: 20000101-20230531; Age Related: All Child: 0-18 years Search modes - Find all my search terms	Interface - EBSCOhost Research Databases Search Screen - Advanced Search Database - MEDLINE Complete	1,935
S28	AB elimination	Limiters - Date of Publication: 20000101-20230531; Age Related: All Child: 0-18 years Search modes - Find all my search terms	Interface - EBSCOhost Research Databases Search Screen - Advanced Search Database - MEDLINE Complete	10,047
S27	AB control	Limiters - Date of Publication: 20000101-20230531; Age Related: All Child: 0-18 years Search modes - Find all my search terms	Interface - EBSCOhost Research Databases Search Screen - Advanced Search Database - MEDLINE Complete	310,833
S26	AB chemotherapy	Limiters - Date of Publication: 20000101-20230531; Age Related: All Child: 0-18 years Search modes - Find all my search terms	Interface - EBSCOhost Research Databases Search Screen - Advanced Search Database - MEDLINE Complete	32,677
S25	AB preventive	Limiters - Date of Publication: 20000101-20230531; Age Related: All Child: 0-18 years Search modes - Find all my search terms	Interface - EBSCOhost Research Databases Search Screen - Advanced Search Database - MEDLINE Complete	24,833
S24	AB guineensis	Limiters - Date of Publication: 20000101-20230531; Age Related: All Child: 0-18 years Search modes - Find all my search terms	Interface - EBSCOhost Research Databases Search Screen - Advanced Search Database - MEDLINE Complete	9
S23	AB s. guineensis	Limiters - Date of Publication: 20000101-20230531; Age Related: All Child: 0-18 years	Interface - EBSCOhost Research Databases Search Screen - Advanced Search	3

		Search modes - Find all my search terms	Database - MEDLINE Complete	
S22	AB intercalatum	Limiters - Date of Publication: 20000101-20230531; Age Related: All Child: 0-18 years Search modes - Find all my search terms	Interface - EBSCOhost Research Databases Search Screen - Advanced Search Database - MEDLINE Complete	12
S21	AB s. intercalatum	Limiters - Date of Publication: 20000101-20230531; Age Related: All Child: 0-18 years Search modes - Find all my search terms	Interface - EBSCOhost Research Databases Search Screen - Advanced Search Database - MEDLINE Complete	8
S20	AB mekongi	Limiters - Date of Publication: 20000101-20230531; Age Related: All Child: 0-18 years Search modes - Find all my search terms	Interface - EBSCOhost Research Databases Search Screen - Advanced Search Database - MEDLINE Complete	25
S19	AB s. mekongi	Limiters - Date of Publication: 20000101-20230531; Age Related: All Child: 0-18 years Search modes - Find all my search terms	Interface - EBSCOhost Research Databases Search Screen - Advanced Search Database - MEDLINE Complete	20
S18	AB japonicum	Limiters - Date of Publication: 20000101-20230531; Age Related: All Child: 0-18 years Search modes - Find all my search terms	Interface - EBSCOhost Research Databases Search Screen - Advanced Search Database - MEDLINE Complete	203
S17	AB s. japonicum	Limiters - Date of Publication: 20000101-20230531; Age Related: All Child: 0-18 years Search modes - Find all my search terms	Interface - EBSCOhost Research Databases Search Screen - Advanced Search Database - MEDLINE Complete	127
S16	AB intestinal schistosomiasis	Limiters - Date of Publication: 20000101-20230531; Age Related: All Child: 0-18 years	Interface - EBSCOhost Research Databases Search Screen - Advanced Search	268

		Search modes - Find all my search terms	Database - MEDLINE Complete	
S15	AB mansoni	Limiters - Date of Publication: 20000101-20230531; Age Related: All Child: 0-18 years<br>Search modes - Find all my search terms	Interface - EBSCOhost Research Databases<br>Search Screen - Advanced Search<br>Database - MEDLINE Complete	1,193
S14	AB s. mansoni	Limiters - Date of Publication: 20000101-20230531; Age Related: All Child: 0-18 years<br>Search modes - Find all my search terms	Interface - EBSCOhost Research Databases<br>Search Screen - Advanced Search<br>Database - MEDLINE Complete	799
S13	AB schistosoma mansoni	Limiters - Date of Publication: 20000101-20230531; Age Related: All Child: 0-18 years<br>Search modes - Find all my search terms	Interface - EBSCOhost Research Databases<br>Search Screen - Advanced Search<br>Database - MEDLINE Complete	1,044
S12	AB s. haematobium	Limiters - Date of Publication: 20000101-20230531; Age Related: All Child: 0-18 years<br>Search modes - Find all my search terms	Interface - EBSCOhost Research Databases<br>Search Screen - Advanced Search<br>Database - MEDLINE Complete	603
S11	AB schistosoma haematobium	Limiters - Date of Publication: 20000101-20230531; Age Related: All Child: 0-18 years<br>Search modes - Find all my search terms	Interface - EBSCOhost Research Databases<br>Search Screen - Advanced Search<br>Database - MEDLINE Complete	731
S10	AB haematobium	Limiters - Date of Publication: 20000101-20230531; Age Related: All Child: 0-18 years<br>Search modes - Find all my search terms	Interface - EBSCOhost Research Databases<br>Search Screen - Advanced Search<br>Database - MEDLINE Complete	806
S9	AB schistosoma	Limiters - Date of Publication: 20000101-20230531; Age Related: All Child: 0-18 years	Interface - EBSCOhost Research Databases<br>Search Screen - Advanced Search	1,785

		Search modes - Find all my search terms	Database - MEDLINE Complete	
S8	AB genital schistosomiasis	Limiters - Date of Publication: 20000101-20230531; Age Related: All Child: 0-18 years Search modes - Find all my search terms	Interface - EBSCOhost Research Databases Search Screen - Advanced Search Database - MEDLINE Complete	62
S7	AB urinary schistosomiasis	Limiters - Date of Publication: 20000101-20230531; Age Related: All Child: 0-18 years Search modes - Find all my search terms	Interface - EBSCOhost Research Databases Search Screen - Advanced Search Database - MEDLINE Complete	279
S6	AB urogenital schistosomiasis	Limiters - Date of Publication: 20000101-20230531; Age Related: All Child: 0-18 years Search modes - Find all my search terms	Interface - EBSCOhost Research Databases Search Screen - Advanced Search Database - MEDLINE Complete	135
S5	AB snail fever	Limiters - Date of Publication: 20000101-20230531; Age Related: All Child: 0-18 years Search modes - Find all my search terms	Interface - EBSCOhost Research Databases Search Screen - Advanced Search Database - MEDLINE Complete	9
S4	AB katayama fever	Limiters - Date of Publication: 20000101-20230531; Age Related: All Child: 0-18 years Search modes - Find all my search terms	Interface - EBSCOhost Research Databases Search Screen - Advanced Search Database - MEDLINE Complete	5
S3	AB bilharzia	Limiters - Date of Publication: 20000101-20230531; Age Related: All Child: 0-18 years Search modes - Find all my search terms	Interface - EBSCOhost Research Databases Search Screen - Advanced Search Database - MEDLINE Complete	29
S2	AB bilharziasis	Limiters - Date of Publication: 20000101-20230531; Age Related: All Child: 0-18 years	Interface - EBSCOhost Research Databases Search Screen - Advanced Search	22

		Search modes - Find all my search terms	Database - MEDLINE Complete	
S1	AB schistosomiasis	Limiters - Date of Publication: 20000101-20230531; Age Related: All Child: 0-18 years Search modes - Find all my search terms	Interface - EBSCOhost Research Databases Search Screen - Advanced Search Database - MEDLINE Complete	2,088

CINAHL (26 April 2023)

EBSCOhost

Tuesday, April 25, 2023 10:16:10 PM

#	Query	Limiters/Expanders	Last Run Via	Results
S39	((S1 OR S2 OR S3 OR S4 OR S5 OR S6 OR S7 OR S8 OR S9 OR S10 OR S11 OR S12 OR S13 OR S14 OR S15 OR S16 OR S17 OR S18 OR S19 OR S20 OR S21 OR S22 OR S23 OR S24) AND (S25 OR S26 OR S27 OR S28 OR S29 OR S30 OR S31 OR S32 OR S33 OR S34 OR S35)) AND (S36 AND S37)	Limiters - Published Date: 20000101-20230531 Search modes - Find all my search terms	Interface - EBSCOhost Research Databases Search Screen - Advanced Search Database - CINAHL	412

#	Query	Limiters/Expanders	Last Run Via	Results
S39	((S1 OR S2 OR S3 OR S4 OR S5 OR S6 OR S7 OR S8 OR S9 OR S10 OR S11 OR S12 OR S13 OR S14 OR S15 OR S16 OR S17 OR S18 OR S19 OR S20 OR S21 OR S22 OR S23 OR S24) AND (S25 OR S26 OR S27 OR S28 OR S29 OR S30 OR S31 OR S32 OR S33 OR S34 OR S35)) AND (S36 AND S37)	Limiters - Published Date: 20000101-20230531 Search modes - Find all my search terms	Interface - EBSCOhost Research Databases Search Screen - Advanced Search Database - CINAHL	412

| S38 | ((S1 OR S2 OR S3 OR S4 OR S5 OR S6 OR S7 OR S8 OR S9 OR S10 OR S11 OR S12 OR S13 OR S14 OR S15 OR S16 OR S17 OR S18 OR S19 OR S20 OR S21 OR S22 OR S23 OR S24) AND (S25 | Search modes - Find all my search terms | Interface - EBSCOhost Research Databases Search Screen - Advanced Search Database - MEDLINE Complete | 1,456 |

	OR S26 OR S27 OR S28 OR S29 OR S30 OR S31 OR S32 OR S33 OR S34 OR S35)) AND (S36 AND S37)			
S37	(S1 OR S2 OR S3 OR S4 OR S5 OR S6 OR S7 OR S8 OR S9 OR S10 OR S11 OR S12 OR S13 OR S14 OR S15 OR S16 OR S17 OR S18 OR S19 OR S20 OR S21 OR S22 OR S23 OR S24) AND (S25 OR S26 OR S27 OR S28 OR S29 OR S30 OR S31 OR S32 OR S33 OR S34 OR S35)	Search modes - Find all my search terms	Interface - EBSCOhost Research Databases Search Screen - Advanced Search Database - MEDLINE Complete	1,456
S36	S1 OR S2 OR S3 OR S4 OR S5 OR S6 OR S7 OR S8 OR S9 OR S10 OR S11 OR S12 OR S13 OR S14 OR S15 OR S16 OR S17 OR S18 OR S19 OR S20 OR S21 OR S22 OR S23 OR S24	Search modes - Find all my search terms	Interface - EBSCOhost Research Databases Search Screen - Advanced Search Database - MEDLINE Complete	2,793
S35	AB molluscicides	Limiters - Date of Publication: 20000101-20230531; Age Related: All Child: 0-18 years<br>Search modes - Find all my search terms	Interface - EBSCOhost Research Databases Search Screen - Advanced Search Database - MEDLINE Complete	15
S34	AB snail control	Limiters - Date of Publication: 20000101-20230531; Age Related: All Child: 0-18 years<br>Search modes - Find all my search terms	Interface - EBSCOhost Research Databases Search Screen - Advanced Search Database -	200

			MEDLINE Complete	
S33	AB mda	Limiters - Date of Publication: 20000101-20230531; Age Related: All Child: 0-18 years<br>Search modes - Find all my search terms	Interface - EBSCOhost Research Databases<br>Search Screen - Advanced Search<br>Database - MEDLINE Complete	1,898
S32	AB mass drug administration	Limiters - Date of Publication: 20000101-20230531; Age Related: All Child: 0-18 years<br>Search modes - Find all my search terms	Interface - EBSCOhost Research Databases<br>Search Screen - Advanced Search<br>Database - MEDLINE Complete	1,366
S31	AB education	Limiters - Date of Publication: 20000101-20230531; Age Related: All Child: 0-18 years<br>Search modes - Find all my search terms	Interface - EBSCOhost Research Databases<br>Search Screen - Advanced Search<br>Database - MEDLINE Complete	94,083
S30	AB hygiene	Limiters - Date of Publication: 20000101-20230531; Age Related: All Child: 0-18 years<br>Search modes - Find all my search terms	Interface - EBSCOhost Research Databases<br>Search Screen - Advanced Search<br>Database - MEDLINE Complete	10,023
S29	AB wash	Limiters - Date of Publication: 20000101-20230531; Age Related: All Child: 0-18 years<br>Search modes - Find all my search terms	Interface - EBSCOhost Research Databases<br>Search Screen - Advanced Search<br>Database -	1,935

			MEDLINE Complete	
S28	AB elimination	Limiters - Date of Publication: 20000101-20230531; Age Related: All Child: 0-18 years<br>Search modes - Find all my search terms	Interface - EBSCOhost Research Databases<br>Search Screen - Advanced Search<br>Database - MEDLINE Complete	10,047
S27	AB control	Limiters - Date of Publication: 20000101-20230531; Age Related: All Child: 0-18 years<br>Search modes - Find all my search terms	Interface - EBSCOhost Research Databases<br>Search Screen - Advanced Search<br>Database - MEDLINE Complete	310,833
S26	AB chemotherapy	Limiters - Date of Publication: 20000101-20230531; Age Related: All Child: 0-18 years<br>Search modes - Find all my search terms	Interface - EBSCOhost Research Databases<br>Search Screen - Advanced Search<br>Database - MEDLINE Complete	32,677
S25	AB preventive	Limiters - Date of Publication: 20000101-20230531; Age Related: All Child: 0-18 years<br>Search modes - Find all my search terms	Interface - EBSCOhost Research Databases<br>Search Screen - Advanced Search<br>Database - MEDLINE Complete	24,833
S24	AB guineensis	Limiters - Date of Publication: 20000101-20230531; Age Related: All Child: 0-18 years<br>Search modes - Find all my search terms	Interface - EBSCOhost Research Databases<br>Search Screen - Advanced Search<br>Database -	9

			MEDLINE Complete	
S23	AB s. guineensis	Limiters - Date of Publication: 20000101-20230531; Age Related: All Child: 0-18 years<br>Search modes - Find all my search terms	Interface - EBSCOhost Research Databases<br>Search Screen - Advanced Search<br>Database - MEDLINE Complete	3
S22	AB intercalatum	Limiters - Date of Publication: 20000101-20230531; Age Related: All Child: 0-18 years<br>Search modes - Find all my search terms	Interface - EBSCOhost Research Databases<br>Search Screen - Advanced Search<br>Database - MEDLINE Complete	12
S21	AB s. intercalatum	Limiters - Date of Publication: 20000101-20230531; Age Related: All Child: 0-18 years<br>Search modes - Find all my search terms	Interface - EBSCOhost Research Databases<br>Search Screen - Advanced Search<br>Database - MEDLINE Complete	8
S20	AB mekongi	Limiters - Date of Publication: 20000101-20230531; Age Related: All Child: 0-18 years<br>Search modes - Find all my search terms	Interface - EBSCOhost Research Databases<br>Search Screen - Advanced Search<br>Database - MEDLINE Complete	25
S19	AB s. mekongi	Limiters - Date of Publication: 20000101-20230531; Age Related: All Child: 0-18 years<br>Search modes - Find all my search terms	Interface - EBSCOhost Research Databases<br>Search Screen - Advanced Search<br>Database -	20

			MEDLINE Complete	
S18	AB japonicum	Limiters - Date of Publication: 20000101-20230531; Age Related: All Child: 0-18 years<br>Search modes - Find all my search terms	Interface - EBSCOhost Research Databases<br>Search Screen - Advanced Search<br>Database - MEDLINE Complete	203
S17	AB s. japonicum	Limiters - Date of Publication: 20000101-20230531; Age Related: All Child: 0-18 years<br>Search modes - Find all my search terms	Interface - EBSCOhost Research Databases<br>Search Screen - Advanced Search<br>Database - MEDLINE Complete	127
S16	AB intestinal schistosomiasis	Limiters - Date of Publication: 20000101-20230531; Age Related: All Child: 0-18 years<br>Search modes - Find all my search terms	Interface - EBSCOhost Research Databases<br>Search Screen - Advanced Search<br>Database - MEDLINE Complete	268
S15	AB mansoni	Limiters - Date of Publication: 20000101-20230531; Age Related: All Child: 0-18 years<br>Search modes - Find all my search terms	Interface - EBSCOhost Research Databases<br>Search Screen - Advanced Search<br>Database - MEDLINE Complete	1,193
S14	AB s. mansoni	Limiters - Date of Publication: 20000101-20230531; Age Related: All Child: 0-18 years<br>Search modes - Find all my search terms	Interface - EBSCOhost Research Databases<br>Search Screen - Advanced Search<br>Database -	799

			MEDLINE Complete	
S13	AB schistosoma mansoni	Limiters - Date of Publication: 20000101-20230531; Age Related: All Child: 0-18 years<br>Search modes - Find all my search terms	Interface - EBSCOhost Research Databases<br>Search Screen - Advanced Search<br>Database - MEDLINE Complete	1,044
S12	AB s. haematobium	Limiters - Date of Publication: 20000101-20230531; Age Related: All Child: 0-18 years<br>Search modes - Find all my search terms	Interface - EBSCOhost Research Databases<br>Search Screen - Advanced Search<br>Database - MEDLINE Complete	603
S11	AB schistosoma haematobium	Limiters - Date of Publication: 20000101-20230531; Age Related: All Child: 0-18 years<br>Search modes - Find all my search terms	Interface - EBSCOhost Research Databases<br>Search Screen - Advanced Search<br>Database - MEDLINE Complete	731
S10	AB haematobium	Limiters - Date of Publication: 20000101-20230531; Age Related: All Child: 0-18 years<br>Search modes - Find all my search terms	Interface - EBSCOhost Research Databases<br>Search Screen - Advanced Search<br>Database - MEDLINE Complete	806
S9	AB schistosoma	Limiters - Date of Publication: 20000101-20230531; Age Related: All Child: 0-18 years<br>Search modes - Find all my search terms	Interface - EBSCOhost Research Databases<br>Search Screen - Advanced Search<br>Database -	1,785

			MEDLINE Complete	
S8	AB genital schistosomiasis	Limiters - Date of Publication: 20000101-20230531; Age Related: All Child: 0-18 years<br>Search modes - Find all my search terms	Interface - EBSCOhost Research Databases<br>Search Screen - Advanced Search<br>Database - MEDLINE Complete	62
S7	AB urinary schistosomiasis	Limiters - Date of Publication: 20000101-20230531; Age Related: All Child: 0-18 years<br>Search modes - Find all my search terms	Interface - EBSCOhost Research Databases<br>Search Screen - Advanced Search<br>Database - MEDLINE Complete	279
S6	AB urogenital schistosomiasis	Limiters - Date of Publication: 20000101-20230531; Age Related: All Child: 0-18 years<br>Search modes - Find all my search terms	Interface - EBSCOhost Research Databases<br>Search Screen - Advanced Search<br>Database - MEDLINE Complete	135
S5	AB snail fever	Limiters - Date of Publication: 20000101-20230531; Age Related: All Child: 0-18 years<br>Search modes - Find all my search terms	Interface - EBSCOhost Research Databases<br>Search Screen - Advanced Search<br>Database - MEDLINE Complete	9
S4	AB katayama fever	Limiters - Date of Publication: 20000101-20230531; Age Related: All Child: 0-18 years<br>Search modes - Find all my search terms	Interface - EBSCOhost Research Databases<br>Search Screen - Advanced Search<br>Database -	5

			MEDLINE Complete	
S3	AB bilharzia	Limiters - Date of Publication: 20000101-20230531; Age Related: All Child: 0-18 years<br>Search modes - Find all my search terms	Interface - EBSCOhost Research Databases<br>Search Screen - Advanced Search<br>Database - MEDLINE Complete	29
S2	AB bilharziasis	Limiters - Date of Publication: 20000101-20230531; Age Related: All Child: 0-18 years<br>Search modes - Find all my search terms	Interface - EBSCOhost Research Databases<br>Search Screen - Advanced Search<br>Database - MEDLINE Complete	22
S1	AB schistosomiasis	Limiters - Date of Publication: 20000101-20230531; Age Related: All Child: 0-18 years<br>Search modes - Find all my search terms	Interface - EBSCOhost Research Databases<br>Search Screen - Advanced Search<br>Database - MEDLINE Complete	2,088

Cochrane library (26 April 2023)

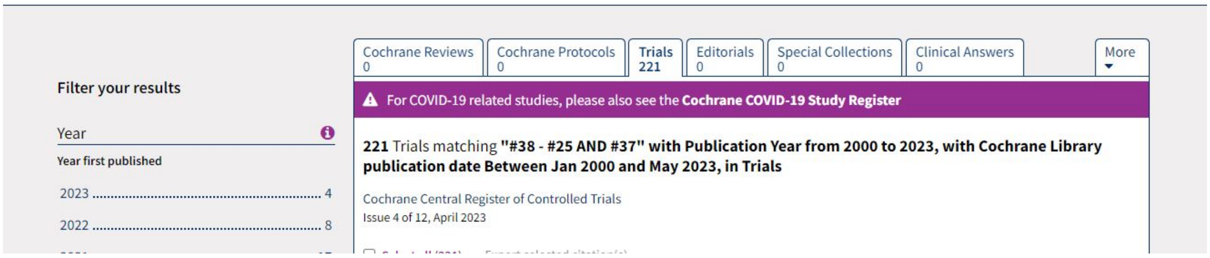

Date Run: 26/04/2023 01:17:28
Comment: cochrane search

ID	Search	Hits
#1	schistosomiasis	716

#2	bilharziasis	38
#3	bilharzia	15
#4	katayama fever	10
#5	snail fever	5
#6	urogenital schistosomiasis	28
#7	urinary schistosomiasis	81
#8	genital schistosomiasis	10
#9	schistosoma haematobium	175
#10	schistosoma	454
#11	haematobium	195
#12	s. haematobium	160
#13	schistosoma mansoni	269
#14	mansoni	336
#15	s. mansoni	220
#16	intestinal schistosomiasis	95
#17	s. japonicum	47
#18	japonicum	93
#19	s. mekongi	6
#20	mekongi	7
#21	s. intercalatum	8
#22	intercalatum	10
#23	s. guineensis	2
#24	guineensis	10
#25	#1 or #2 or #3 or #4 or #5 or #6 or #7 or #8 or #9 or #10 or #11 or #12 or #13 or #14 or #15 or #16 or #17 or #18 or #19 or #20 or #21 or #22 or #23 or #24862	
#26	preventive	25988
#27	chemotherapy	95209
#28	elimination	14090
#29	wash	9555
#30	sanitation	863
#31	hygiene	16479
#32	mass drug administration	16693
#33	mda	4062
#34	snail control	45
#35	molluscicides	2
#36	molluscicidal	2
#37	#26 or #27 or #29 or #30 or #31 or #32 or #33 or #34 or #35 or #36162765	
#38	#25 AND #37 with Publication Year from 2000 to 2023, with Cochrane Library publication date Between Jan 2000 and May 2023, in Trials	221

Other databases

MedRxiv (29 April 2023)

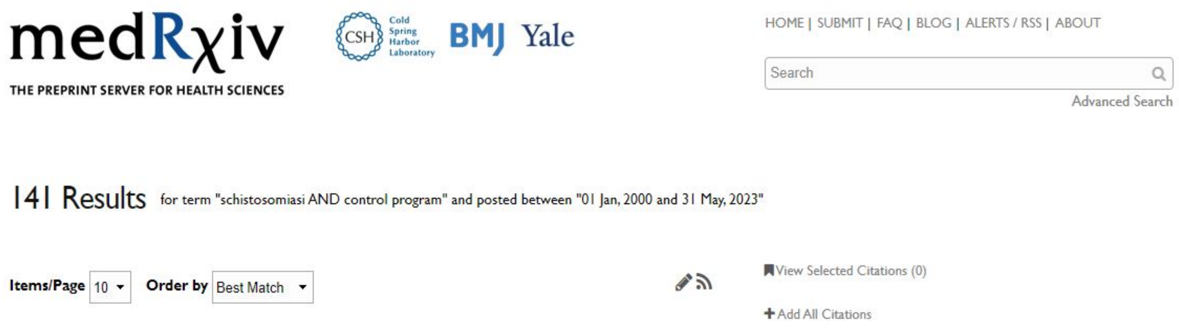

BioRxiv (29 April 2023)

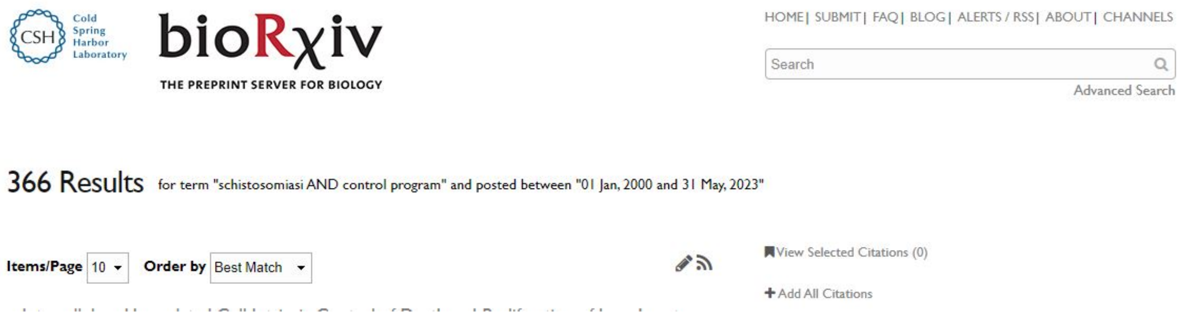

Supplement: S4 File — This is the search strategy that was used in review’s search process for articles relevant to the study. (PDF) [file pone.0301464.s006.pdf]
