# Supplementary material for: Effects of paediatric schistosomiasis control programmes in sub-Saharan Africa: A systematic review
Source: PLoS One. 2024 May 2;19(5):e0301464. doi: 10.1371/journal.pone.0301464 (PMC11065241; doi:10.1371/journal.pone.0301464)
Supplement: Attachment — Submitted filename: PONE 2 09 JAN.docx [file pone.0301464.s008.docx]

Dear, authors. Thank you for submitting a revised version of your manuscript. However, I have raised three remaining concerns about the manuscript. Please address and provide the respective considerations, if possible.

2) Line 66 -- I also suggest mentioning interventions to improve access to water and infrastructure for its use (such as piped water, tap water and other domestic structures), as these are those most associated with schistosomiasis.

6) Ok, behavioral information was explained in the results summary table. But it needs to be indicated that it was information extracted from the articles by the authors. Please add the extracted data to the list. Item 5, line 155.

12) Ok. But how many articles were excluded because they dealt with control programs outside Sub-Saharan Africa? How many articles were excluded because they dealt with adults? How many excluded because they were treating other diseases? How many due to the effectiveness of prazinquentel? This information is important for the replicability of the systematic review and recommended by the PRISMA method. At least some of these could be contained in the flowchart in Figure 1.
